# B_12_‐Catalyzed Carbonylation of Carbon Tetrahalides: Using a Broad Range of Visible Light to Access Diverse Carbonyl Compounds

**DOI:** 10.1002/chem.202403663

**Published:** 2024-11-18

**Authors:** Keita Shichijo, Miho Tanaka, Yohei Kametani, Yoshihito Shiota, Mamoru Fujitsuka, Hisashi Shimakoshi

**Affiliations:** ^1^ Department of Chemistry and Biochemistry Graduate School of Engineering Kyushu University Nishi-ku, Motooka Fukuoka 744, 819–0395 Japan; ^2^ Institute for Materials Chemistry and Engineering Kyushu University Nishi-ku, Motooka Fukuoka 744, 819–0395 Japan; ^3^ SANKEN (The Institute of Scientific and Industrial Research) Osaka University,Mihogaoka 8–1 Ibaraki, Osaka 567-0047 Japan

**Keywords:** Carbonylation, Visible Light, Vitamin B_12_, Photocatalyst, Dual Catalysis

## Abstract

Visible‐light‐driven organic synthesis is a green and sustainable method for producing fine chemicals and is highly desirable at both laboratory and industrial scales. In this study, we developed a broad‐range (including the red region) visible‐light‐driven carbonylation of CCl_4_, CBr_4_, and CBr_3_F with nucleophiles, such as amines and alcohols, using a B_12_−Mg^2+^/TiO_2_ hybrid catalyst. Carbonyl molecules such as ureas, carbamates, carbonate esters, and carbamoyl fluorides were synthesized with high selectivity and efficiency under mild conditions. Diffuse reflectance UV‐vis spectroscopy, femtosecond time‐resolved diffuse reflectance spectroscopy, and density functional theory calculations revealed the reaction mechanism is a combination of *S_N_2* and single‐electron transfer. This is a rare example of a low‐energy, red‐light‐driven photocatalysis, which has been a highly desired organic reaction in recent years. We believe that this study provides a general platform to access diverse carbonyl molecules and could promote photocatalytic carbonylation reactions on a pilot scale.

## Graphical abstract

A visible‐light‐driven carbonylation of CCl_4_, CBr_4_, and CBr_3_F have been developed using a B_12_−Mg^2+^/TiO_2_ hybrid catalyst. This method enables the selective synthesis of carbonyl molecules, including ureas, carbamates, carbonate esters, and carbamoyl fluorides, under mild conditions. The reaction mechanism involves *S_N_2* and single‐electron transfer, driven by broad‐range visible light. This study highlights a general platform for green and sustainable photocatalytic carbonylation reactions, extending to low‐energy red‐light applications.

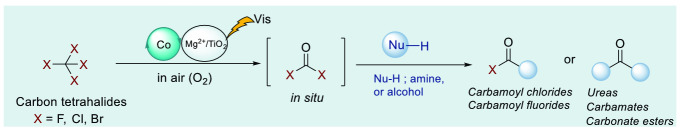

## Introduction

Carbonyl molecules, such as ureas, carbamates, and carbonate esters, are valuable building blocks for the synthesis of fine chemicals and functional polymers.[^1^, ^2^] They are also used as drug molecules in the field of pharmacology.[^3^, ^4^] These carbonyl molecules are classically synthesized from phosgene (COCl_2_), a useful C_1_ building block, which is produced annually at over 8 million tons.^[5]^ However, carbonylation reactions with COCl_2_ are highly toxic and are thus avoided. Therefore, various alternative carbonylation reactions using triphosgene,[^6^, ^7^] oxamic acids,[^8^, ^9^] CO,[^10^, ^11^] and CO_2_[^12^, ^13^, ^14^, ^15^] have been reported over the past two decades. Furthermore, photoinduced carbonylation reactions have recently attracted considerable attention. In these reactions, carbonylation of chloroform is achieved under UV (254 nm)[^16^, ^17^, ^18^] or visible (405 nm)^[19]^ light irradiation via a photoradical pathway. Carbamoyl fluorides are another important class of carbonyl molecules due to their remarkable stability and distinct chemical properties. These molecules are used as potent covalent inhibitors of protease and esterase or competent electrophiles in cross‐coupling reactions.[^20^, ^21^] They can be directly synthesized from fluorophosgene (COF_2_) and amines; however, commercial sources of COF_2_ are unavailable.[^22^, ^23^] In recent years, alternative methods have been developed using excellent COF_2_ reservoirs,^[24]^ such as trifluoromethyl triflate,^[25]^ AgOCF_3_,^[26]^ organic trifluoromethoxide salt,^[27]^ and oxamic acids with fluoride salt.^[28]^ However, to the best of our knowledge, the photocatalytic synthesis of carbamoyl fluorides has never been reported. Therefore, a general platform for accessing diverse carbonyl molecules, including ureas, carbamates, carbonate esters, and carbamoyl fluorides, via photoradical pathways is highly desired from the viewpoint of green and sustainable chemistry.

Metal complex‐mediated radical organic synthesis is a powerful method for fine chemical synthesis with high selectivity and efficiency.[^29^, ^30^, ^31^, ^32^] In particular, cobalt complexes (especially vitamin B_12_) provide unique reactivity for a variety of radical organic synthesis.[^33^, ^34^, ^35^, ^36^] The “supernucleophilic” Co(I) reacts with an electrophile, such as alkyl halides, alkyl tosylates, and alkyl epoxides, producing an organometallic complex (Co(III)‐C complex).^[37]^ This Co(III)‐C bond dissociates under controlled conditions, such as electrolysis, photolysis, and thermolysis, to afford alkyl radicals, which can then participate in unique radical organic transformations including Giese‐type reactions,^[38]^ cross‐coupling reactions,^[39]^ olefin formation,^[40]^ radical cyclization,[^41^, ^42^] and CO_2_ reduction.^[43]^ Furthermore, in 2023, an atom transfer radical polymerization reaction mediated by B_12_ derivative^[44]^ was developed, which brought B_12_‐catalysis for organic synthesis back into the spotlight again.

Dual catalysis of B_12_ derivatives and photosensitizers, such as organic dyes,[^45^, ^46^] metal complexes,^[47]^ TiO_2_,^[48]^ and metal–organic frameworks^[49]^ facilitates green and sustainable photoradical organic synthesis. Photoexcited photosensitizers efficiently reduce the central cobalt to generate Co(I) species, which produces a Co(III)‐C complex. Hence, light‐driven organic synthesis reactions using B_12_ dual catalysis are a significant area of research. Although B_12_ dual catalysis are ideal for the development of photoradical organic synthesis because of the excellent radical reservoir of the Co(III)‐C complex, a general platform to access diverse carbonyl molecules via the photoradical pathway has not yet been established.

**Figure 1:**
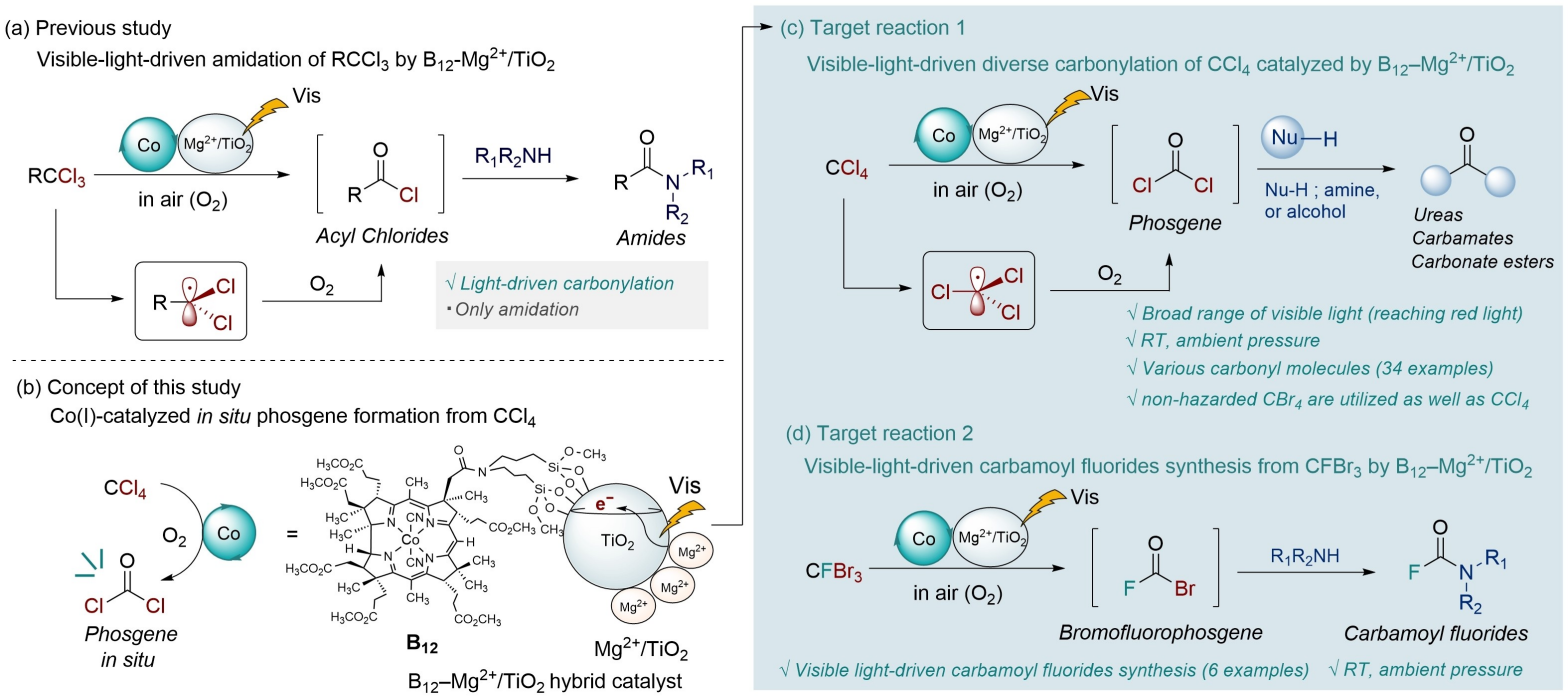
Previous study: visible light‐driven amidation of RCCl_3_ by B_12_−Mg^2+^/TiO_2_; (b) Concept of this study; (c) Target reaction 1: visible light‐driven diverse carbonylation of CCl_4_ catalyzed by B_12_−Mg^2+^/TiO_2_; (d) Target reaction 2: visible light‐driven synthesis of carbamoyl fluorides from CFBr_3_ by B_12_−Mg^2+^/TiO_2_.

Based on our previous study on B_12_ dual catalysis,^[50]^ we believe that the B_12_ hybrid catalyst (B_12_−Mg^2+^/TiO_2_), consisting of a B_12_ derivative (B_12_) and a magnesium ion‐modified TiO_2_ (Mg^2+^/TiO_2_), is a potential general platform for photocatalytic carbonylation reactions (Figure 1 a).^[51]^ B_12_−Mg^2+^/TiO_2_ can produce Co(I) over a broad range of visible light, and Co(I) reacts with RCCl_3_ in the presence of O_2_ to produce acyl chlorides (RCOCl). Finally, RCOCl reacts with amines to yield amides. Therefore, we hypothesized that B_12_−Mg^2+^/TiO_2_ would convert CCl_4_ to COCl_2_ in situ in the presence of the Co(III)‐CCl_3_ complex, affording diverse carbonyl molecules under visible‐light irradiation (Figure 1 b). This B_12_ hybrid catalyst may also facilitate a low‐energy, red‐light‐driven carbonylation reaction.

In this study, we developed a novel visible‐light‐driven carbonylation reaction with CCl_4_ catalyzed by the B_12_−Mg^2+^/TiO_2_ hybrid catalyst (Figure 1 c). This photocatalytic reaction can provide access to diverse carbonyl molecules, including symmetric and unsymmetric ureas, carbamates, and carbonate esters, in air at room temperature and ambient pressure over a broad range of visible light. Spectroscopy, electrochemistry, and density functional theory (DFT) calculations were used to elucidate the reaction mechanism. Other carbon tetrahalides (CX_4_), such as CBr_4_, which is non‐hazardous, was also applied to this reaction in place of CCl_4_ (Figure 1 c). Interestingly, the reaction of CBr_3_F with amines produced valuable carbamoyl fluoride, which was one of the serendipitous findings of this study (Figure 1 d).

## Results and Discussion

### Synthesis of Symmetric Carbonyl Molecules from CCl_4_

**Figure 2:**
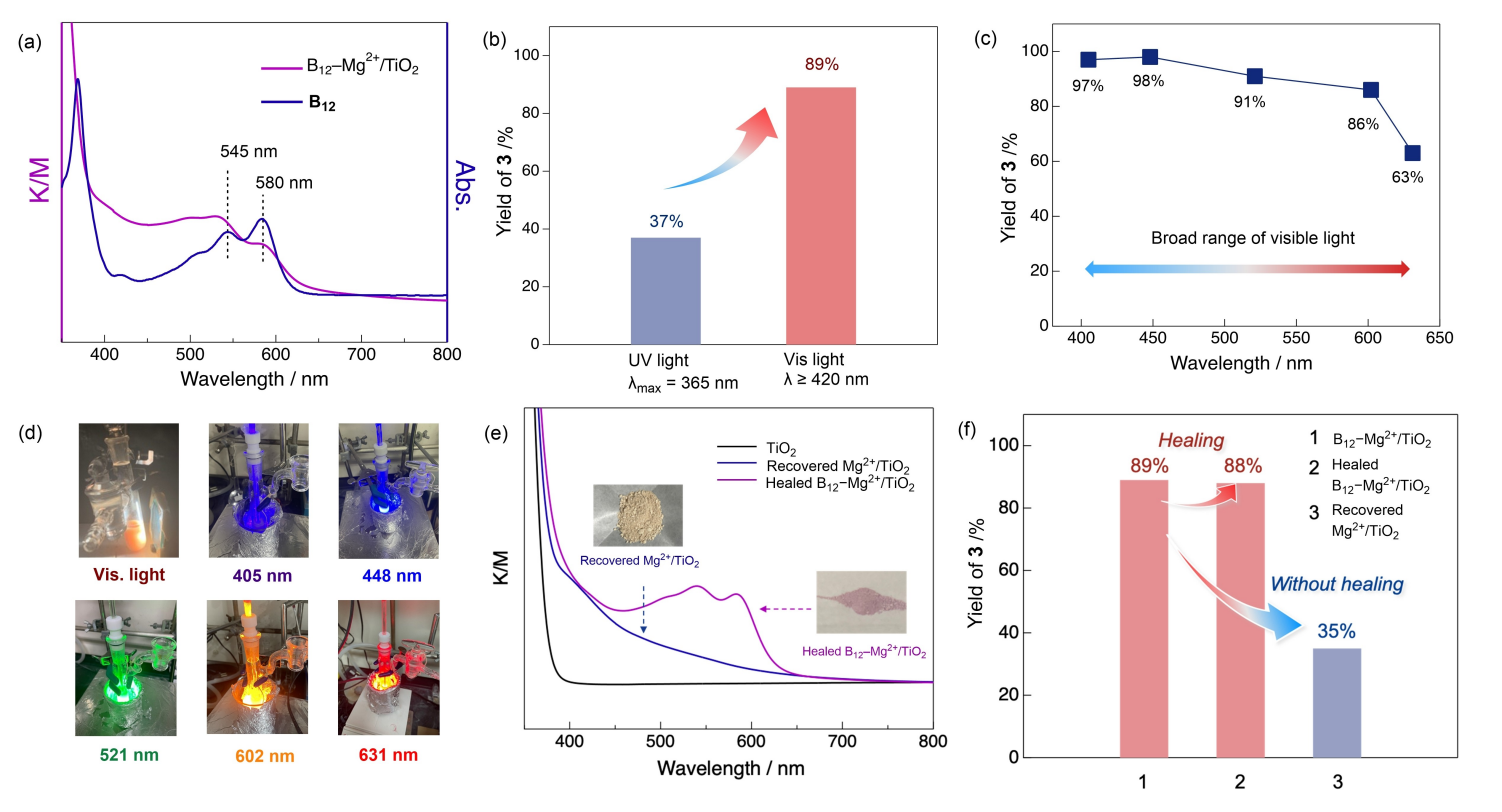
Effect of light irradiation on B_12_−Mg^2+^/TiO_2_ for desired reactivity and effect of healing treatment on the recyclability of B_12_−Mg^2+^/TiO_2_. (a) DR‐UV‐vis spectrum of B_12_−Mg^2+^/TiO_2_ and UV‐vis spectrum of B_12_ in CH_3_OH; (b) Comparison of the reactivity of B_12_−Mg^2+^/TiO_2_ under UV and visible light irradiation; (c) Limitation of visible light irradiation during 24 h; (d) Reaction setups; (e) DR‐UV‐vis spectra of TiO_2_, Recovered Mg^2+^/TiO_2_, and Healed B_12_−Mg^2+^/TiO_2_; and (e) Recycling test using Healed B_12_−Mg^2+^/TiO_2_ and Recovered Mg^2+^/TiO_2_.

B_12_−Mg^2+^/TiO_2_ was synthesized according to a previously described procedure.^[51]^ The diffuse reflectance (DR)‐UV‐vis spectrum of B_12_−Mg^2+^/TiO_2_ shows characteristic peaks at 580 and 545 nm (Figure 2 a), which is consistent with the Q‐band absorption of B_12_. Other characterizations of this photocatalyst are described in the Supporting Information.

A visible light‐driven synthesis of 1,1,3,3‐tetraethylurea (**3**) was performed in air at room temperature using CCl_4_ (**1**), diethylamine (**2**), *N,N*‐diisopropylethylamine (DIPEA), and B_12_−Mg^2+^/TiO_2_ as the substrate, nucleophile, reductant, and photocatalyst, respectively. The optimized amounts of B_12_−Mg^2+^/TiO_2_, nucleophile **2**, DIPEA at the optimal reaction time (Tables S4‐1‐S4‐3) afforded the desired compound **3** in 89 % GC‐MS yield with a TON of 438 based on B_12_ (entry 1, Table 1). Control experiments (entries 2–4, Table 1) indicated that B_12_−Mg^2+^/TiO_2_, visible‐light irradiation, and DIPEA were essential for promoting this reaction. Investigation into the individual photocatalysts Mg^2+^/TiO_2_ revealed that Mg^2+^/TiO_2_ was less effective than B_12_−Mg^2+^/TiO_2_ in affording **3** (entry 5 Table 1). This could be attributed to the insufficient redox potential of C.B. (*E*_C.B._) of Mg^2+^/TiO_2_ for the desired one‐electron reduction of **1**. This demonstrates that B_12_ on the Mg^2+^/TiO_2_ surface promotes the desired reactivity. The use of B_12_−TiO_2_ as the catalyst drastically decreased the yield of **3** to 10 % (entry 6, Table 1). This confirms that the immobilization of Mg^2+^ ions on TiO_2_ plays a significant role in responding to visible‐light irradiation. The reaction catalyzed by a B_12_ derivative, **C1**, (Figure S2‐1) and Mg^2+^/TiO_2_ without immobilization demonstrated that the immobilization of B_12_ on the Mg^2+^/TiO_2_ surface enhanced the desired catalytic activity (entry 7, Table 1).

**Table 1:** Visible‐light‐driven symmetric urea synthesis from CCl_4_.^[a]^

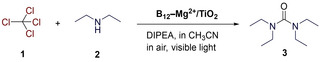			
**Entry**	**change from standard condition**	**yield (%)** ^***b***^	**TON** ^***c***^
1	None	89	438
2	no irradiation	0	0
3	no B_12_−Mg^2+^/TiO_2_	0	–
4	no DIPEA	14	69
5^*d*^	Mg^2+^/TiO_2_ as catalyst	35	–
6^*e*^	B_12_–TiO_2_ as catalyst	10	50
7^*f*^	B_12_ (**C1**)+Mg^2+^/TiO_2_ as catalyst	66	325
8^*g*^	solar simulator	78	384

^[a]^ Reaction conditions: [**1**]=6.0×10^−3^ M (0.06 mmol), [DIPEA]=1.0×10^−1^ M (17 equiv), [**2**]=6.0×10^−2^ M (10 equiv), B_12_−Mg^2+^/TiO_2_=20 mg ([B_12_] =1.2×10^−5^ M (0.2 mol %)), solvent: 10 mL of CH_3_CN, light source: 200 W tungsten lamp with 42 L cut‐off filter (*λ* ≥420 nm) and a heat cut‐off filter (Sigma Koki, 30 H), reaction time: 6 hours. [b]Yield of products were based on the initial concentration of the substrate. [c]TON based on B_12_. [d]Mg^2+^/TiO_2_ (20 mg) was used as catalyst. [e]B_12_−Mg^2+^/TiO_2_ (20 mg) was used as catalyst. [f][B_12_ complex, dicyano heptamethyl cobyrinate (**C1**)]=1.2×10^−5^ M (0.2 mol %), Mg^2+^/TiO_2_=20 mg. [g]Sunlight simulator.

The effect of various light sources on the reactivity of the catalyst to synthesize compound **3** was investigated. UV light irradiation (λ_max_=365 nm) for TiO_2_ excitation drastically decreased the yield of **3** (37 %, Figure 2 b). This is because high‐energy UV light decomposes the COCl_2_ intermediate. We also investigated the limitations of the wavelengths available for this reaction. LEDs with wavelengths shorter than 602 nm afforded **3** in over 85 % (Figure 2 c). Importantly, the yield of **3** remained at 63 % with a red LED at 631 nm (Figure 2 c). Thus, B_12_−Mg^2+^/TiO_2_ is operable over a broad range of visible light, even in the red region. This is consistent with the DR‐UV‐vis spectrum of Mg^2+^/TiO_2_ (Figure S3‐3, blue line). The development of photocatalytic organic syntheses using red light, which has excellent penetration depth, has been strongly desired but it is still challenging due to its weak energy. In recent year, a few innovative examples have been achieved.[^52^, ^53^, ^54^] This photocatalytic carbonylation catalyzed by B_12_−Mg^2+^/TiO_2_ under irradiation of red LED at 631 nm, also serves as a promising candidate to promote the advancement of red light‐driven photocatalytic organic synthesis. Furthermore, a solar simulator effectively accelerated the reaction, producing **3** in 78 % yield (entry 8, Table 1). These experimental results suggest the generality of the B_12_−Mg^2+^/TiO_2_‐catalyzed visible‐light‐driven carbonylation of CCl_4_ via the photoradical pathway.

The recyclability of B_12_−Mg^2+^/TiO_2_ was investigated using the process shown in Figure S4‐3. After the first reaction, the photocatalyst was separated from the suspension by centrifugation and characterized using DR‐UV‐vis spectroscopy. While B_12_ derivatives exhibit high stability and reactivity in an N_2_ atmosphere,^[50]^ they decompose in the presence of reactive oxygen species under aerobic conditions. Thus, the characteristic absorption of the B_12_‐derivatives disappeared after the reaction, indicating they were unmodified on the recovery photocatalyst (Figure 2 e, blue line). However, visible light absorption from 400 to 650 nm was retained on the recovered photocatalyst (Figure 2 e, blue line). This suggests that the modification of Mg^2+^ persisted after the photocatalytic reaction in air. Thus, the photocatalytic activity can be recovered after the “healing treatment,” in which the B_12_ is again modified on the surface of Mg^2+^/TiO_2_. The healing treatment of B_12_−Mg^2+^/TiO_2_ involved modifying B_12_ on the recovered Mg^2+^/TiO_2_. The DR‐UV‐vis spectrum of the healed B_12_−Mg^2+^/TiO_2_ showed the characteristic Q‐band of B_12_, suggesting that B_12_ was again modified on the TiO_2_ surface (Figure 2 e, magenta line). The use of the healed B_12_−Mg^2+^/TiO_2_ produced **3** in 88 % yield, similar to the reaction that used unmodified B_12_−Mg^2+^/TiO_2_ (Figure 2 f). However, the recovered Mg^2+^/TiO_2_ without the healing treatment was less effective than the healed B_12_−Mg^2+^/TiO_2_ (Figure 2 f) for the reaction. This indicates the importance of the healing treatment, which remodifies B_12_ on the recovered Mg^2+^/TiO_2_, for recovering the desired reactivity. The gram‐scale synthesis of **3** (Scheme S4‐1) was conducted using 1.52 g of **1** and produced 1.05 g of **3** in 61 % isolated yield (67 % GC‐MS yield). Thus, the photocatalytic synthesis of **3** by B_12_−Mg^2+^/TiO_2_ proved to be a highly productive system.

The substrate scope for visible‐light‐driven symmetric urea synthesis from **1** catalyzed by B_12_−Mg^2+^/TiO_2_, is summarized in Table 2 a. The characterization of the products is described in the Supporting Information. Symmetric urea **3** was obtained in 89 % yield via the reactions of **1** and **2**. Secondary amines with various substituents, including linear alkyl groups (**4**–**6**) and aryl groups (**7**–**8**), were tolerated under the optimized conditions, producing the corresponding symmetric ureas in high yields. Ureas bearing cyclic amine substituents (**9**–**12**) were successfully produced in good yields. The primary amine also afforded the desired symmetric ureas (**13**–**14**) in moderate yields. Moreover, the use of phenols in place of amines yielded carbonate esters. Diphenyl carbonate (DPC; **15**), which is a valuable chemical,[^55^, ^56^] was obtained in 43 % yield.

**Table 2:**
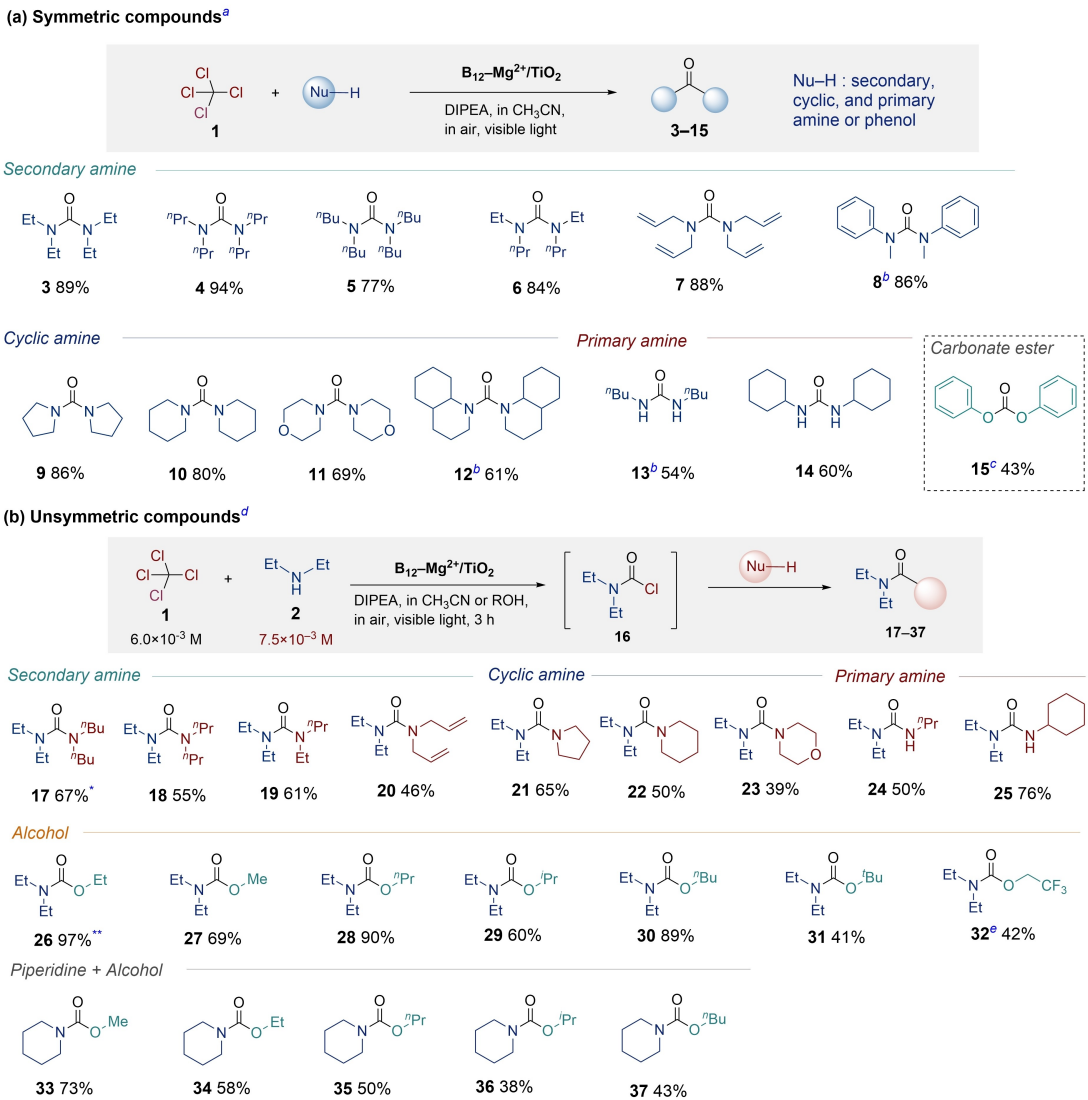
Substrate scope of visible light‐driven carbonylation catalyzed by B_12_−Mg^2+^/TiO_2_.

^[a]^ Reaction conditions: [**1**]=6.0×10^−3^ M (0.06 mmol), [DIPEA]=1.0×10^−1^ M (17 equiv), [Amine]=6.0×10^−2^ M (10 equiv), B_12_−Mg^2+^/TiO_2_=20 mg ([B_12_]=1.2×10^−5^ M (0.2 mol %)), solvent: 10 mL of CH_3_CN, light source: 200 W tungsten lamp with 42 L cut‐off filter (*λ* ≥420 nm) and a heat cut‐off filter (Sigma Koki, 30 H), reaction time: 6 h. [b][Amine]=7.5×10^−3^ M (1.3 equiv), [c][Phenol]=6.0×10^−1^ M (100 equiv), [d]Reaction conditions (**17**–**25**): [**1**]=6.0×10^−3^ M (0.06 mmol), [DIPEA]=1.0×10^−1^ M (17 equiv), [**2**]=7.5×10^−3^ M (1.3 equiv), [Amine]=6.0×10^−2^ M (10 equiv), B_12_−Mg^2+^/TiO_2_=20 mg ([B_12_]=1.2×10^−5^ M (0.2 mol %)), solvent: 10 mL of CH_3_CN. Reaction conditions (**26**–**37**): [**1**]=6.0×10^−3^ M (0.06 mmol), [DIPEA]=1.0×10^−1^ M (17 equiv), [Amine]=7.5×10^−3^ M(1.3 equiv), B_12_−Mg^2+^/TiO_2_=20 mg ([B_12_]=1.2×10^−5^ M (0.2 mol %)), solvent: 10 mL of alcohol. [e]Solvent: CF_3_CH_2_OH : CH_3_CN=1 : 1. ^*^Symmetric urea **3** was observed in ca.1 % by GC. ^**^Symmetric urea **3** was not observed by GC.

Synthesis of unsymmetric carbonyl compounds from CCl_4_. We investigated visible light‐driven unsymmetric urea synthesis from **1** catalyzed by B_12_−Mg^2+^/TiO_2_. The selective synthesis of carbamoyl chlorides is a key procedure in this synthesis (Figure S4‐1). Thus, the optimized reaction conditions and reaction time were determined for the highly selective diethylcarbamoyl chloride (**16**) synthesis (Table S4‐4). 1,1,3,3‐Dibutyldiethylurea (**17**) was synthesized under these optimized conditions as a standard reaction. After performing the photocatalytic reaction between **1** and **2** for 3 h, dibutylamine was added to the suspension, and the reaction suspension was stirred for 21 h in the dark (Figure S4‐1), which yielded unsymmetric urea **17** in 67 % yield. This demonstrates that visible‐light‐driven B_12_−Mg^2+^/TiO_2_ photocatalysis successfully afforded both unsymmetric and symmetric urea. The substrate scope for the synthesis of unsymmetric urea from **1** is summarized in Table 2 b. Various amines such as secondary amines (**18**–**20**), cyclic amines (**21**–**23**), and primary amines (**24**–**25**) were tolerated under the optimized conditions, affording the corresponding unsymmetric ureas in moderate yields. Overall, visible‐light‐driven unsymmetric urea synthesis was achieved in a two‐step one‐pot process using B_12_−Mg^2+^/TiO_2_.

Carbamates are also significant unsymmetric carbonyl compounds in the fields of materials chemistry and pharmacology. Interestingly, ethyl *N,N*‐diethylcarbamate (**26**) was formed as the main product in 97 % yield instead of **3** when the reaction was performed using **1** and **2** in ethanol instead of acetonitrile. However, no diethyl carbonate was obtained despite the use of ethanol as a solvent, confirming the highly selective one‐pot synthesis of **26**. To understand this high selectivity, DFT calculations for the reaction of COCl_2_ and ethanol (Figure S5‐1) and the reaction of COCl_2_ and **2** (Figure S5‐2) were performed. The activation energy of the reaction between COCl_2_ and ethanol was estimated to be 26.4 kcal/mol, which is almost 10 times higher than that of the reaction between COCl_2_ and **2** (2.89 kcal/mol). This suggests that COCl_2_ reacts only with **2** and not with ethanol, affording carbamoyl chloride (**16**). DFT calculations were also performed for the reaction of **16** and ethanol (Figure S5‐3). The activation energy of this step was estimated to be 21.4 kcal/mol, demonstrating that this step proceeds satisfactorily at room temperature. Although the activation energy of the reaction between **16** and **2** was 6.2 kcal/mol lower than that between **16** and ethanol (Figure S5‐3, S5‐4), symmetric urea would not be produced under the optimized conditions. This is because the amount of nucleophile **2** is insufficient (entry 5, Table S4‐4). Therefore, **16** reacts only with ethanol to afford **26** with high selectivity. The substrate scope for the carbamate synthesis is summarized in Table 2 b. The reactions between **1** and **2** in various alcohols produced the corresponding carbamates (**26**–**32**) in excellent to moderate yields. In particular, *tert*‐butylalcohol yielded **31**, which is a novel method for the synthesis of Boc‐protected amines. Fluorine‐containing carbamate **32** was successfully synthesized via this reaction. Moreover, the reaction of **1** and piperidine performed in various alcohols afforded corresponding carbamates (**33**–**37**) in moderate yields.

### Mechanistic Study

**Figure 3:**
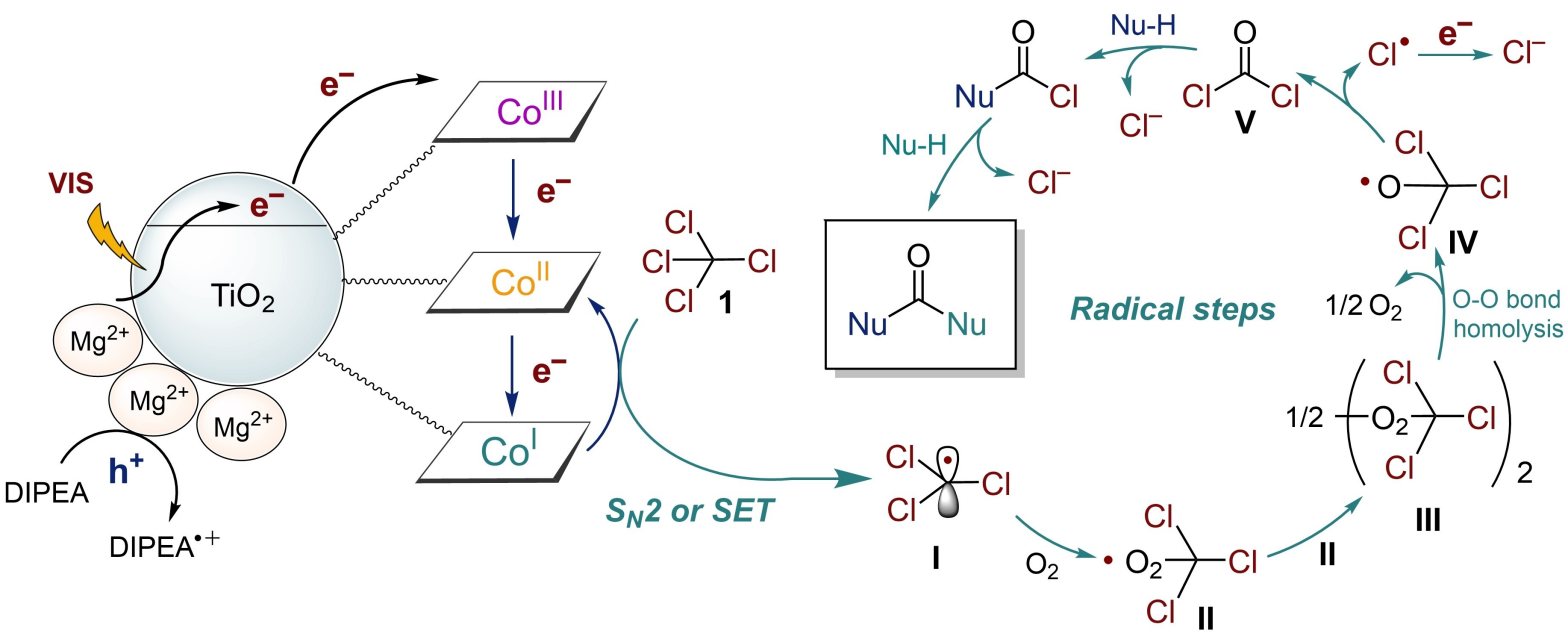
Catalytic mechanism for visible light‐driven carbonylation of CCl_4_ with nucleophiles.

**Figure 4:**
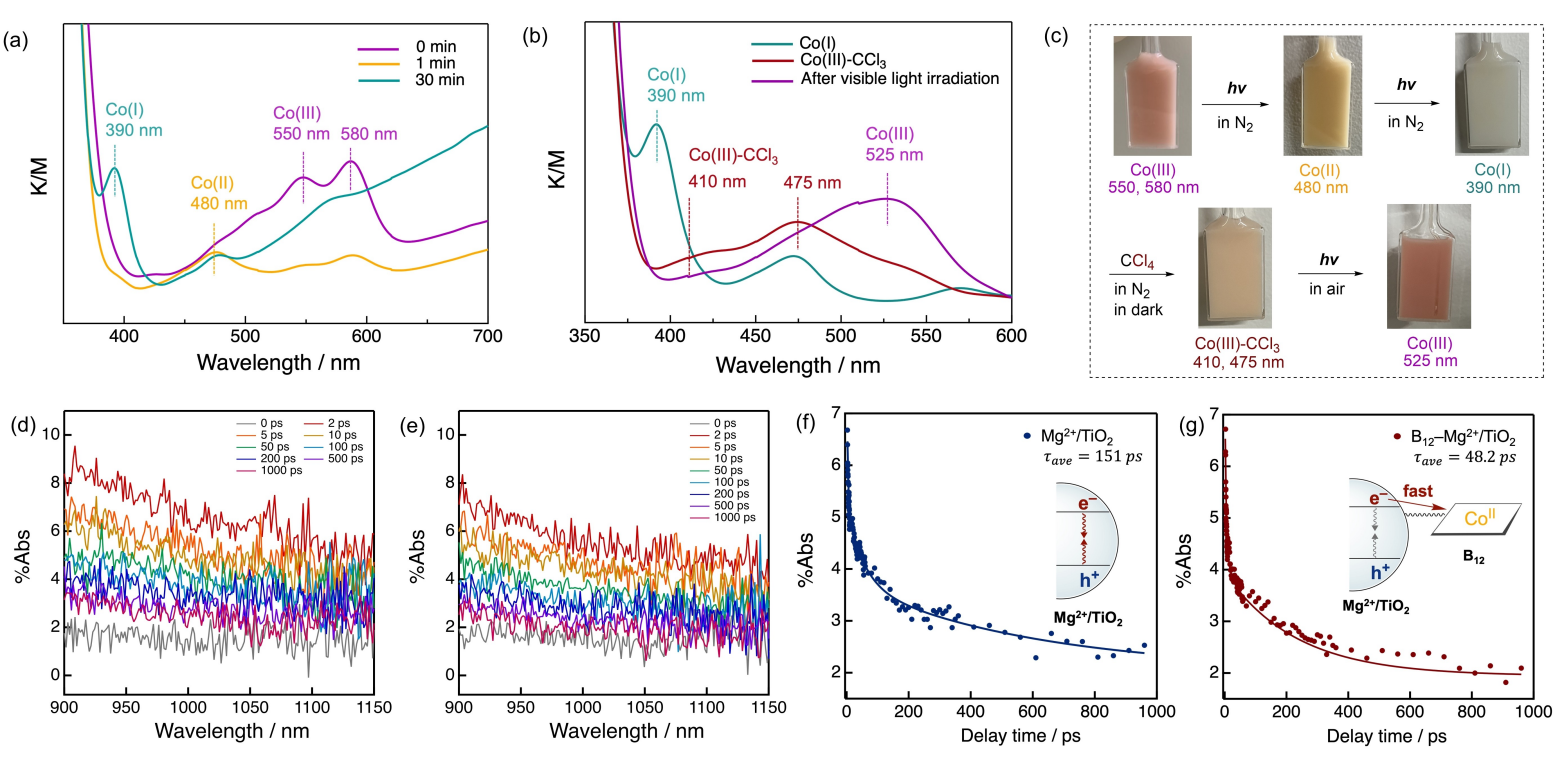
Reactivity of Co(I) toward CCl_4_ and electron transfer in B_12_−Mg^2+^/TiO_2_. (a) DR‐UV‐vis spectral changes of B_12_−Mg^2+^/TiO_2_ in visible light irradiation of 0, 1, and 30 min. (b) DR‐UV‐vis spectral changes of B_12_−Mg^2+^/TiO_2_ after generating Co(I), adding CCl_4_ in the dark, and irradiating visible light under aerobic conditions. (d) Femtosecond time‐resolved diffuse reflectance (fs‐TDR) spectrum of (d) Mg^2+^/TiO_2_ and (e) B_12_−Mg^2+^/TiO_2_ during laser flash photolysis using a 405‐nm femtosecond pulse for excitation. (f) Kinetic trace of (f) Mg^2+^/TiO_2_ and (g) B_12_−Mg^2+^/TiO_2_ at %Abs at ~1000–1050 nm during the laser flash photolysis. The solid lines are the fitted curves using a multi‐exponential function.

The reaction mechanism was studied through various experiments and DFT calculations. The proposed reaction mechanism is illustrated in Figure 3. Co(I) formation on the Mg^2+^/TiO_2_ surface was monitored by DR‐UV‐vis spectroscopy under visible‐light irradiation (Figure 4 a). B_12_−Mg^2+^/TiO_2_ suspended in acetonitrile containing triethylamine showed the characteristic absorption maxima of Co(II) and Co(I) at 480 nm and 390 nm after 1 min and 30 min, respectively, of visible‐light irradiation (Figure 4 a). This suggests that B_12_−Mg^2+^/TiO_2_ responds readily to visible light and effectively produces Co(I). The reactivity of Co(I) toward **1** was investigated by DR‐UV‐vis spectroscopy (Figure 4 b). When **1** was added to the suspension containing Co(I), new peaks quickly appeared while the Co(I) peaks disappeared (Figure 4 b). This spectrum is consistent with that of the Co(III)‐CCl_3_ complex reported previously.^[57]^ This confirms that Co(I) effectively reacted with **1** to form a Co(III)‐CCl_3_ complex. The suspension was continuously irradiated with visible light for a few minutes under aerobic conditions. The resultant spectrum indicated the presence of Co(III) (Figure 4 b), which demonstrates that the Co−C bond was homolytically cleaved by visible light irradiation, and Co(II) was readily oxidized by oxygen. In the CV curves of the B_12_ derivative (heptamethyl cobyrinate, **C2**; Figure S2‐1) in the presence of **1**, a reductive current of Co(III)‐CCl_3_ was observed at −1.34 V *vs*. Ag/AgCl (Figure S5‐5, S5‐6). These results suggest that an *S_N_2* reaction occurred between Co(I) and CCl_4_.

The electron transfer behavior from Mg^2+^/TiO_2_ to B_12_ on Mg^2+^/TiO_2_ was determined by analyzing Mg^2+^/TiO_2_ and B_12_−Mg^2+^/TiO_2_ via femtosecond time‐resolved diffuse reflectance (fs‐TDR) spectroscopy.^[58]^ Upon a 405‐nm pump light excitation, these samples showed broad absorptions of excited electrons at 950–1050 nm (Figure 4 c,d). The weighted average lifetime of the excited electron of B_12_−Mg^2+^/TiO_2_ was 48.2 ps, with time constants of *τ_1_=*1.22 ps (67 %), *τ_2_* =9.71 ps (13 %), and *τ_3_=*230 ps (20 %). The weighted average lifetime of Mg^2+^/TiO_2_ was 151 ps, with time constants of *τ_1_* =2.33 ps (57 %), *τ_2_* =52.6 ps (20 %), and *τ_3_* =599 ps (23 %) (Figure 4 e,f). The fs‐TDR spectra of these samples showed a 3.1 times faster excited electron lifetime for B_12_−Mg^2+^/TiO_2_ than for Mg^2+^/TiO_2_. This confirms charge migration from Mg^2+^/TiO_2_ to B_12_.

Next, the radical steps following Co−C bond homolysis were examined. The use of the spin‐trapping reagent 5,5‐dimethyl‐1‐pyrroline *N*‐oxide (DMPO) afforded **3** in poor yield (4 %, Scheme S4‐2 a), indicating that this reaction is mediated by alkyl radicals. When the reaction was performed in an N_2_ atmosphere (Scheme S4‐2 b), the yield of **3** dramatically decreased to <1 %. When the reaction was performed under an ^18^O_2_ atmosphere, compound **3** with ^18^O_2_ (*m/z*=174) was clearly observed instead of that with ^16^O_2_ (*m/z*=172) (Scheme S4‐2 c,d). This suggests that the O‐atom on the carbonyl group in **3** was supplied by molecular oxygen in air. Moreover, all Cl atoms were removed as Cl^−^ ions. The number of Cl^−^ ions in the reaction solution was quantified using X‐ray fluorescence (XRF) measurements, wherein 0.74 wt % (4 equiv.) of Cl^−^ ions were detected (Scheme S4‐2 e).

**Figure 5:**
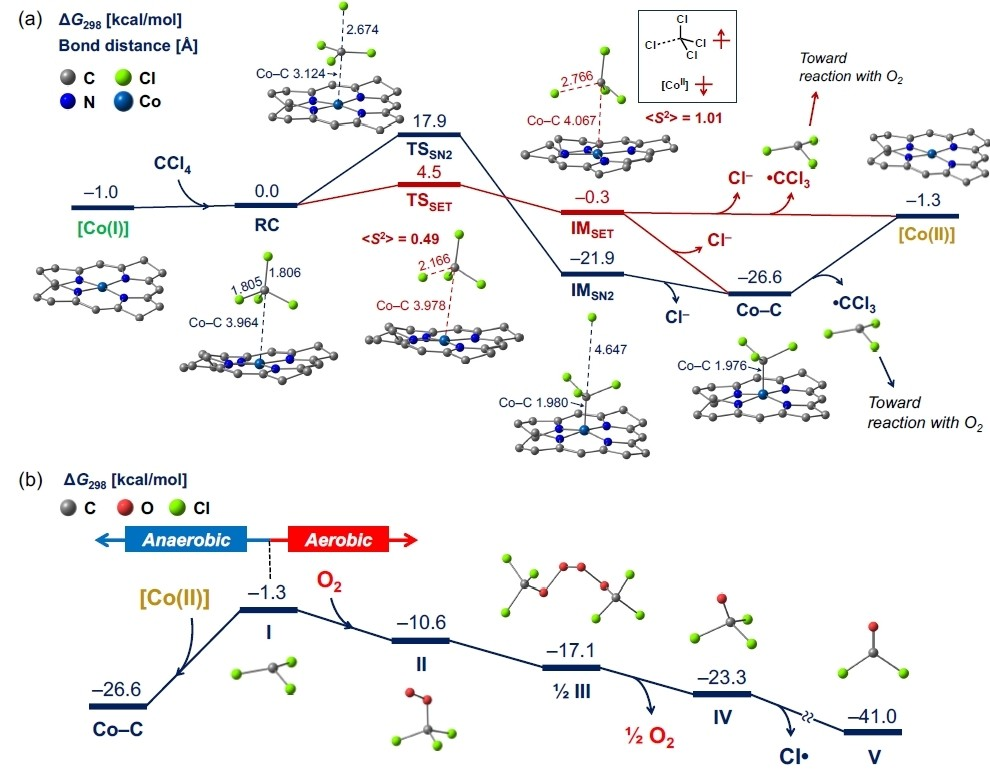
Free energy profiles of (a) the dechlorination of CCl_4_ into ⋅CCl_3_ by a nucleophilic Co(I) species and (b) the reaction of ⋅CCl_3_ with O_2_. Free energies at 298.15 K and bond distances are shown in kcal/mol and Å, respectively. All H‐atoms are omitted for clarity.

Finally, DFT calculations were performed to elucidate the reaction mechanism. We have previously reported the reaction mechanisms of alkylated complex formation and acyl chloride formation.[^59^, ^60^] Based on those reports, we hypothesized a reaction pathway and obtained an energy profile based on DFT calculations (Figure 5 a). After the formation of a reactant complex (**RC**), two reaction mechanisms can be allowed: *S_N_2* mechanism and SET mechanism. In the *S_N_2* mechanism, the cleavage of the C−Cl bond and the formation of the Co−C bond proceed simultaneously to form an alkylated complex with Cl^−^ (**IM_SN2_**) via the transition state **TS_SN2_**. The *S_N_2* reaction is exothermic at 21.9 kcal/mol with an activation energy of 17.9 kcal/mol. The release of Cl^−^ from **IM_SN2_** leads to the formation of an alkylated complex (Co−C). The endothermic bond dissociation of Co−C (25.3 kcal/mol) indicates that it is a catalytically metastable state. Continuous light irradiation can induce homolytic cleavage of the Co−C bond, yielding Co(II) and ⋅CCl_3_. In the SET mechanism, electron transfer from Co(I) to CCl_4_ results in a radical intermediate (**IM_SET_**; Co(II) ⋅⋅⋅⋅CCl_3_⋅⋅⋅Cl^−^) via the transition state **TS_SET_**. The SET reaction is exothermic (0.3 kcal/mol) with an activation energy of 4.5 kcal/mol. Subsequently, the spontaneous release of Cl^−^ and ⋅CCl_3_ generates the Co(II) species. Thus, the activation energies for the *S_N_2* mechanism and the SET mechanism were calculated to be 17.9 and 4.5 kcal/mol, respectively. According to these results, both mechanisms can proceed under thermal condition while the SET mechanism is kinetically preferable for the dechlorination of CCl_4_.

⋅CCl_3_, which is produced by the dechlorination of CCl_4_, reacts with the O_2_ molecule from the air, as shown in Figure 5 b. The association of ⋅CCl_3_ (**I**) with O_2_ leads to the peroxy radical species ⋅O_2_CCl_3_ (**II**). The coupling of II affords the tetraoxide species (CCl_3_O_2_)_2_ (**III**). The cleavage of the two O−O bonds of **III** results in O_2_ and the radical species ⋅OCCl_3_ (**IV**) and the subsequent elimination of Cl⋅ occurs to form phosgene (**V**). The formation of phosgene from ⋅CCl_3_ and O_2_ is exothermic at 39.7 kcal/mol. Under aerobic conditions, the produced ⋅CCl_3_ rapidly reacts with O_2_ as described above. Conversely, under anaerobic conditions, ⋅CCl_3_ may bind to the Co(II) species, forming the thermo‐dynamically stable Co–CCl_3_ complex (**Co−C**). In fact, **Co−C** was detected in the DR‐UV‐vis spectra in an N_2_ atmosphere (Figure 4 b).

### Carbonylation of CBr_4_

CBr_4_ is commercially available and non‐hazardous. It is widely used in reactions such as radical bromination, alkene functionalization, and heterocycle synthesis.[^61^, ^62^, ^63^] The annual growth rate of CBr_4_ is estimated to be 3.70 % during the forecast period of 2023–2032.

**Scheme 1:**
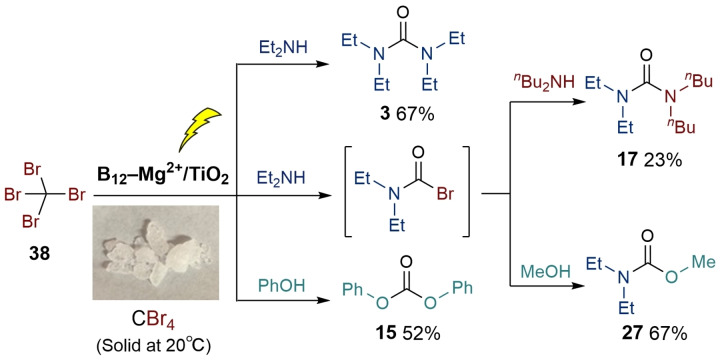
Visible light‐driven carbonylation with CBr_4_ catalyzed by B_12_−Mg^2+^/TiO_2_.

Similar to the photocatalytic carbonylation of CCl_4_, CBr_4_ should be converted to bromophosgene (COBr_2_) using B_12_−Mg^2+^/TiO_2_ under visible‐light irradiation in air, affording diverse carbonyl molecules. Hence, we applied CBr_4_ (**38**) to our photocatalytic carbonylation (Scheme 1).

The reaction amounts of **38**, B_12_−Mg^2+^/TiO_2_, **2**, and DIPEA were optimized for the reaction of **38** and **2** (Tables S4‐5, S4‐6). Compound **3** was obtained in 67 % yield under the optimized conditions (Scheme 1). Other carbonyl compounds such as DPC (**15**), unsymmetric urea (**17**), and carbamate (**27**) were also formed in moderate‐to‐low yields (Scheme 1). This suggests that the non‐hazardous CBr_4_ can be used to produce the target ureas, carbamates, and carbonate esters instead of CCl_4_.

### Carbamoyl Fluoride Formation from CBr_3_F

We developed a visible light‐driven carbamoyl fluoride synthesis with tribromofluoromethane (CBr_3_F) using B_12_−Mg^2+^/TiO_2_. CBr_3_F is converted into bromofluorophosgene (COBrF) as an intermediate. Generally, C−Br bond activation toward nucleophilic substitution is easier than C−F bond activation because Br^−^ is a better leaving group than F^−^. Thus, we hypothesized that COBrF, generated from CBr_3_F, could afford carbamoyl fluorides with high selectivity and efficiency.

Visible light‐driven synthesis of dibutylcarbamic fluoride (**40**) was realized using tribromofluoromethane (CFBr_3_; **39**), dibutylamine, DIPEA, and B_12_−Mg^2+^/TiO_2_ as the substrates, nucleophiles, reductants, and photocatalysts, respectively, in air at room temperature. After 6 h of visible‐light irradiation, **40** was produced in 94 % yield under optimized conditions (Table 3). Control experiments demonstrated that B_12_−Mg^2+^/TiO_2_ and visible‐light irradiation were necessary for this reaction (Table S4‐7). Various secondary amines, including linear alkyl groups (**40**–**42**) and aryl groups (**43**–**45**), were tolerated under the optimized conditions, producing the corresponding carbamoyl fluorides in excellent yields (Table 3). These results demonstrate that B_12_−Mg^2+^/TiO2 is a powerful tool for photocatalytic carbamoyl fluoride synthesis.

**Table 3:**
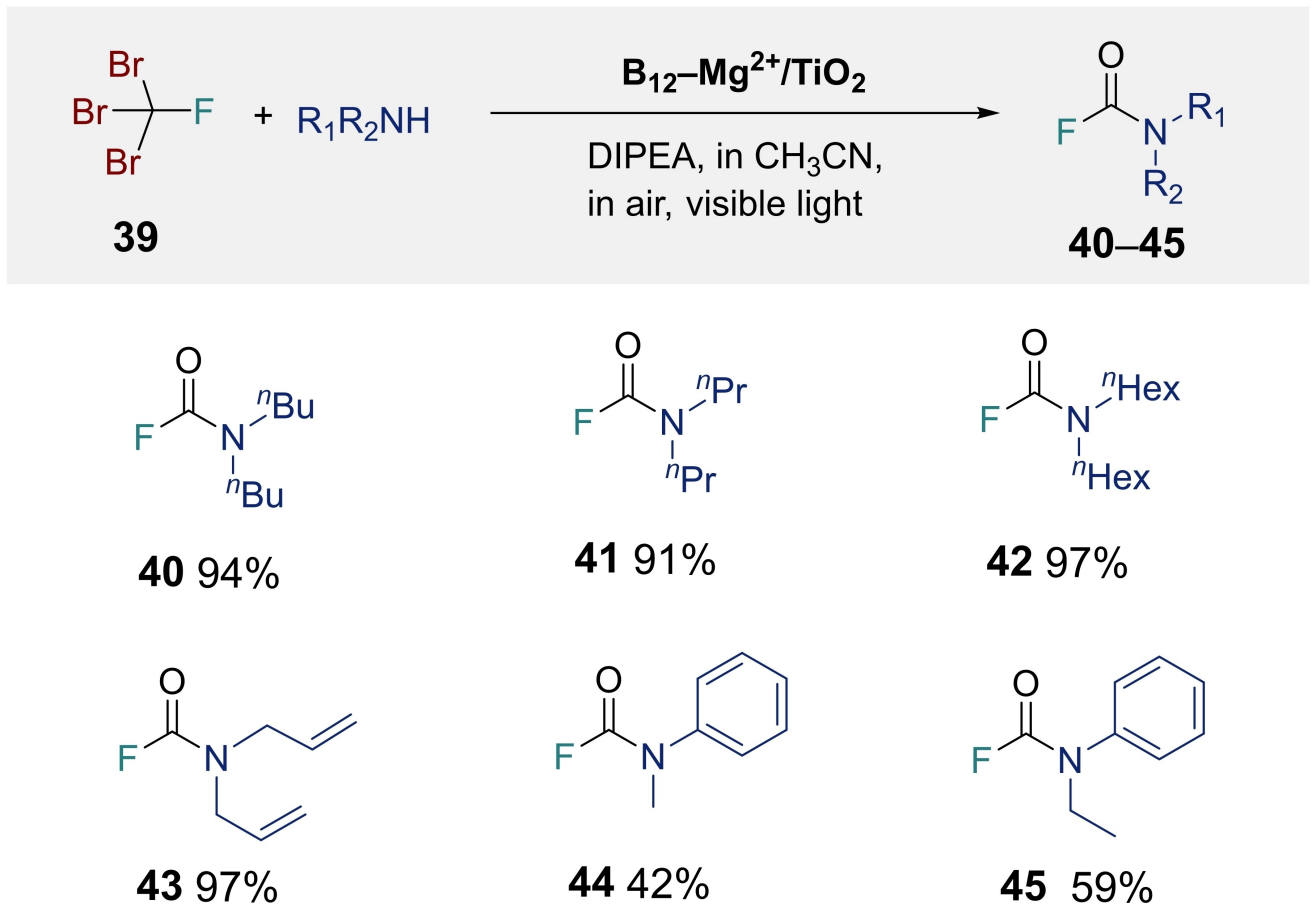
Visible light‐driven carbamoyl fluoride formation from CBr_3_F catalyzed by B_12_−Mg^2+^/TiO_2_.^[a]^

^[a]^ Reaction conditions: [**39**]=6.0×10^−3^ M (0.06 mmol), [DIPEA]=1.0×10^−1^ M (17 equiv), [Amine]=6.0×10^−2^ M (10 equiv), B_12_−Mg^2+^/TiO_2_=20 mg ([B_12_]=1.2×10^−5^ M (0.2 mol %)), solvent: 10 mL of CH_3_CN, light source: 200 W tungsten lamp with 42 L cut‐off filter (*λ* ≥420 nm) and a heat cut‐off filter (Sigma Koki, 30 H), reaction time: 6 h.

## Conclusions

We developed a photocatalytic carbonylation of the carbon tetrahalides CCl_4_, CBr_4_, and CFBr_3_ using the hybrid B_12_−Mg^2+^/TiO_2_ catalyst under visible‐light irradiation to generate diverse carbonyl compounds, such as symmetric and unsymmetric ureas, carbamates, and carbamoyl fluorides, in air at ambient pressure and room temperature.

The light‐induced Co(I) species reacts with CCl_4_ to produce COCl_2_ as the intermediate, which reacts in situ with nucleophiles such as amines and phenols to produce symmetric ureas and carbonate esters, respectively. The optimized amount of amines and reaction time afforded carbamoyl chlorides with high selectivity. Unsymmetric ureas and carbamates were also produced via this reaction with high selectivity and efficiency. In addition, this reaction was successfully performed under low‐energy visible‐light (red‐light) irradiation. Moreover, gram‐scale synthesis of tetraethyl urea was achieved, which suggests that this reaction can be adapted to a pilot scale.

The reaction mechanism was elucidated via spectroscopy and DFT calculations. fs‐TDR spectra showed 3.1 times faster excited electron lifetime for B_12_−Mg^2+^/TiO_2_ than for Mg^2+^/TiO_2_. This indicated that excited electrons were transferred to B_12_ from the conduction band on Mg^2+^/TiO_2_ and reduced the central cobalt to the supernucleophilic Co(I) species. DR‐UV‐vis spectra confirmed the Co(III)‐CCl_3_ complex as the intermediate in this reaction. In contrast, the DFT calculations indicated that the SET mechanism is kinetically preferred for the dechlorination of CCl_4_ by the Co(I) species. This suggested a combination of *S_N_2* and SET mechanisms. These radical steps were confirmed by radical trapping, ^18^O_2_ labeling, and XRF experiments.

Notably, the commercially available and non‐hazardous CBr_4_ afforded various carbonyl compounds, whereas CBr_3_F afforded carbamoyl fluorides through this reaction. This novel photocatalytic method using different CX_4_ molecules provides a green and sustainable strategy to realize carbonylation reactions.

## Supporting Information Summary

Experimental details of the photocatalyst preparation, reaction optimization, mechanistic experiments, and product characterization data.

## Conflict of Interests

The authors declare no conflict of interest.

1

## Supporting information

As a service to our authors and readers, this journal provides supporting information supplied by the authors. Such materials are peer reviewed and may be re‐organized for online delivery, but are not copy‐edited or typeset. Technical support issues arising from supporting information (other than missing files) should be addressed to the authors.

Supporting Information

## Data Availability

The data that support the findings of this study are available from the corresponding author upon reasonable request.
