# Supplementary material for: B12‐Catalyzed Carbonylation of Carbon Tetrahalides: Using a Broad Range of Visible Light to Access Diverse Carbonyl Compounds
Source: Chemistry. 2024 Nov 18;31(2):e202403663. doi: 10.1002/chem.202403663 (PMC11724252; doi:10.1002/chem.202403663)
Supplement: Supplementary file 1 — Supporting Information [file CHEM-31-e202403663-s001.pdf]

Chemistry–A European Journal

Supporting Information

B_{12}-Catalyzed Carbonylation of Carbon Tetrahalides: Using a Broad Range of Visible Light to Access Diverse Carbonyl Compounds

Keita Shichijo, Miho Tanaka, Yohei Kametani, Yoshihito Shiota, Mamoru Fujitsuka, and Hisashi Shimakoshi*

B_{12}-Catalyzed Carbonylation of Carbon Tetrahalides: Using a Broad Range of Visible Light to Access Diverse Carbonyl Compounds

(Supporting Information)

Keita Shichijo,[a] Miho Tanaka,[a] Yohei Kametani,[b] Yoshihito Shiota,[b] Mamoru Fujitsuka,[c] Hisashi Shimakoshi*[a]

[a]Department of Chemistry and Biochemistry, Graduate School of Engineering, Kyushu University, Nishi-ku, Motooka, Fukuoka, 744, 819-0395, Japan. [b]Institute for Materials Chemistry and Engineering, Kyushu University, Nishi-ku, Motooka, Fukuoka, 744, 819-0395, Japan. [c]SANKEN (The Institute of Scientific and Industrial Research), Osaka University, Mihogaoka, 8-1, Ibaraki, Osaka, 567-0047, Japan.

E-mail: shimakoshi@mail.cstm.kyushu-u.ac.jp

Table of Contents

1. General Information 1

2. Experimental details 2

3. Characterization of Hybrid Catalysts 7

4. Photocatalytic reactions 10

4-1. Reaction optimizations 10

4-2. Gram-scale synthesis of 1,1,3,3-tetrathylurea from CCl_4. 12

4-3. Healing treatment and recyclability test of the hybrid photocatalyst. 13

4-4. Mechanistic studies. 14

4-5. Optimization of photocatalytic carbonylation with CBr_4. 15

5. Mechanistic Studies 17

6. Products characterizations 21

7. DFT Calculations 69

8. References 91

1. General Information

Reagents. All the solvents and chemicals used in this study were reagent grade. Commercial reagents were purchased from TCI, Sigma-Aldrich, Wako, and other commercial suppliers, and were used as received unless otherwise noted. The TiO_2 (anatase type, AMT-600, surface area = 52 m^2g^{-1}, diameter size = ca. 30 nm) was supplied from Tayca Co., Ltd. The B_{12} complex, $(CN)_2Cob(III)6C_1esterCON\{(CH_2)_3Si(OMe)_3\}_2$ (**B_{12}**) (Figure S2-1), was synthesized by a reported method.[1] The B_{12}–Mg^{2+}/TiO_2 hybrid photocatalyst were prepared according to the literature (Scheme S2-1, S2-2). The products were isolated by chromatography (Silica Gel 60 N, spherical, neutral) using n-hexane and ethyl acetate as eluent, and identified by ^1H NMR, ^{13}C NMR, IR, and GC-MS. Authentic samples of the products were purchased from TCI.

Equipment. The NMR spectra were recorded by Bruker Avance NEO 400 spectrometer. The UV-vis absorption spectra were measured by Hitachi U-3300 spectrophotometer at room temperature. The infrared (IR) spectra measurements were carried out by a Perkin-Elmer Spectrum Two using the ATR method. The diffuse reflectance (DR)-UV–vis spectra were measured by a JASCO V-770 spectrophotometer equipped with a Φ 60 integrating sphere at room temperature. The X-ray diffraction (XRD) measurements were analyzed by a Rigaku SmartLab. The X-ray fluorescence (XRF) measurements were recorded by Shimadzu EDX-700. The Energy dispersive X-ray spectroscopy analyses (EDX) were measured by a Hitachi SU1500. The SEM image was acquired using a Hitachi SU8000 (acceleration voltage of 15 kV). The cyclic voltammograms were obtained using a BAS CV 50W electrochemical analyzer. A three-electrode cell equipped with a 3.0-mm diameter glassy carbon rod and 1.6-mm diameter platinum wire as the working and counter electrode were used, respectively. An Ag/AgCl (3.0 M NaCl) was used as a reference electrode. The $E_{1/2}$ value of ferrocene/ferrocenium (Fc/Fc^+) was +0.44 V *vs.* Ag/AgCl with this setup. The GC-MS spectra were obtained using a Shimadzu GC-QP5050A equipped with a J&W Scientific DB-1 column (length 30 m; ID 0.25 mm, film 0.25 μm). The 200W tungsten lamp with a 420 nm cut-off filter (Sigma Koki, 42L) and a heat cut-off filter (Sigma Koki, 30H) were used as the light source for the visible light irradiation experiments. A black light, λ_{max} = 365 nm and 1.5 $mWcm^{-2}$, was used for the UV light irradiation. Various wavelength of LEDs (Techno Sigma, PER-AMP) was used as light source. An ASAHI SPECTRA HAL-320 W with a compact xenon light source was utilized as the solar simulator.

2. Experimental details

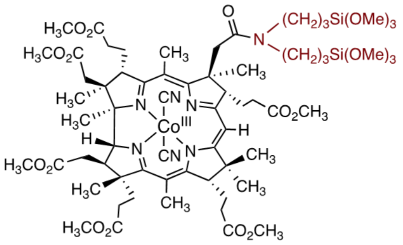

B_{12}

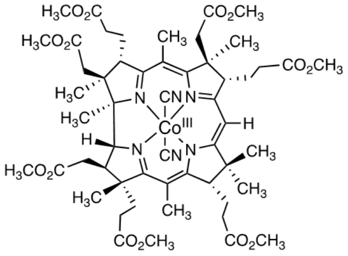

C1

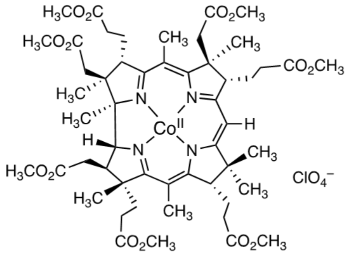

C2

Figure S2-1. Structures of B_{12} complexes.

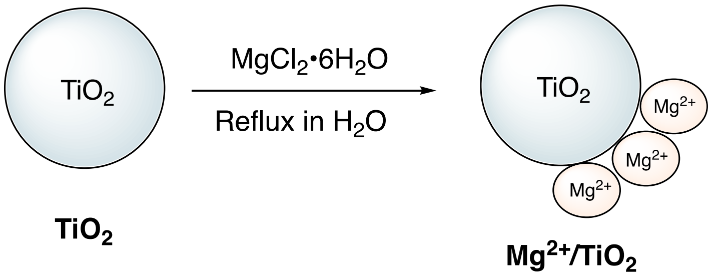

TiO_2

Mg^{2+}/TiO_2

Scheme S2-1. Preparation of Mg^{2+}/TiO_2.

Preparation of Mg^{2+}/TiO_2 photocatalyst. The magnesium ions modified TiO_2 (Mg^{2+}/TiO_2) was prepared according to previous papers.[1] 1.0 g of anatase type TiO_2 was added to 10 mL of a $MgCl_2 \cdot 6H_2O$ (0.16 mmol) aqueous solution and the suspension was refluxed for 2 hours. The suspension was filtered and washed three times with water to remove the chloride ions. The prepared powder was dried at 100℃ degrees for 24 hours, then the powder was calcinated in a muffle furnace at 200℃ degrees for 2 hours. Yield is 901 mg. The Mg^{2+}/TiO_2 was characterized by DR-UV-vis spectroscopy (Figure S3-3).

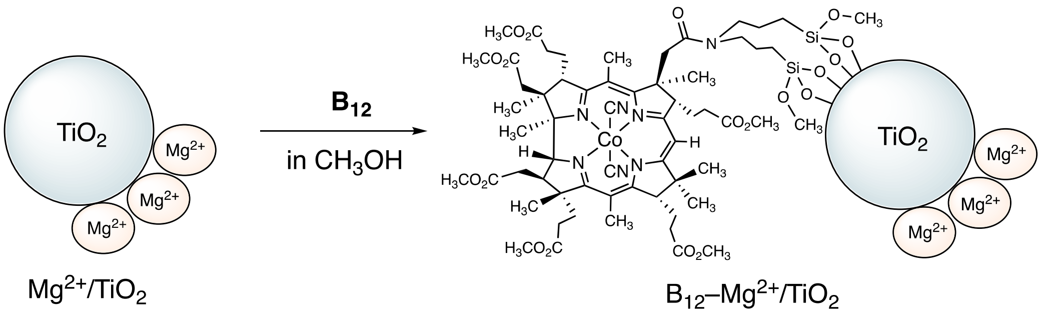

Scheme S2-2. Preparation of B_{12}–Mg^{2+}/TiO_2.

Preparation of B_{12}–Mg^{2+}/TiO_2 hybrid photocatalyst. The B_{12}–Mg^{2+}/TiO_2 hybrid photocatalyst was prepared based on previous papers.[1] 150 mg of the Mg^{2+}/TiO_2 powder was suspended in 1.5 mL of the methanol solution containing the B_{12} complex (5.5 mg 3.9×10^{-3} mmol) and the suspension was stirred for 24 hours in dark. The mixture was centrifuged and washed three times with methanol. The obtained violet powder was dried for 3 hours under vacuum at room temperature. Yield is 114 mg. The loading amount of the B_{12} complex and the Mg^{2+} ions on the surface of the TiO_2 was determined from the UV-vis spectroscopy and EDX analyses, respectively. The B_{12}-Mg^{2+}/TiO_2 was characterized by DR-UV-vis spectroscopy, IR, XRD, and SEM measurements.

Visible light-driven symmetric urea synthesis from CCl_4. A mixture of carbon tetrachloride (9.2 mg, 6.0×10^{-3} M), diethylamine (44 mg, 6.0×10^{-2} M), *N,N*-diisopropylethylamine (129 mg, 1.0×10^{-2} M) and B_{12}–Mg^{2+}/TiO_2 (20 mg, [**B_{12}**] = 6.1×10^{-6} M) was stirred in 10 mL of CH_3CN under air at room temperature. The suspension was stirred for 6 hours during visible light irradiation ($\lambda \geqq 420$ nm) and then the B_{12}–Mg^{2+}/TiO_2 was separated by a filter. The resulting solution was analyzed by GC-MS and yields of the products were calculated by comparison to the ratio of the peak area using diphenyl as an internal standard.

Visible light-driven unsymmetric urea synthesis from CCl_4. A reaction procedure is illustrated in Figure S4-1. A mixture of carbon tetrachloride (9.2 mg, 6.0×10^{-3} M), diethylamine (5.5 mg, 7.5×10^{-3} M), *N,N*-diisopropylethylamine (129 mg, 1.0×10^{-2} M) and B_{12}–Mg^{2+}/TiO_2 (20 mg, [**B_{12}**] = 6.1×10^{-6} M) was stirred in 10 mL of CH_3CN under air at room temperature. After 3 hours light irradiation for this suspension, dibutylamine (78 mg, 6.0×10^{-2} M) was added to the suspension. The suspension was stirred for 21 hours in dark under air

at room temperature and then the B_{12}–Mg^{2+}/TiO_2 was separated by a filter. The resulting solution was analyzed by GC-MS and yields of the products were calculated by comparison to the ratio of the peak area using diphenyl as an internal standard.

Visible light-driven carbamate synthesis from CCl_4. A mixture of carbon tetrachloride (9.2 mg, 6.0×10^{-3} M), diethylamine (5.5 mg, 7.5×10^{-3} M), *N,N*-diisopropylethylamine (129 mg, 1.0×10^{-2} M) and B_{12}–Mg^{2+}/TiO_2 (20 mg, [**B_{12}**] = 6.1×10^{-6} M) was stirred in 10 mL of CH_3OH under air at room temperature. The suspension was stirred for 24 hours during visible light irradiation ($\lambda \geqq 420$ nm) and then the B_{12}–Mg^{2+}/TiO_2 was separated by a filter. The resulting solution was analyzed by GC-MS and yields of the products were calculated by comparison to the ratio of the peak area using diphenyl as an internal standard.

Gram-scale synthesis of 1,1,3,3-tetrathylurea (3) from CCl_4. A mixture of carbon tetrachloride (1.5 g, 1.0×10^{-1} M), diethylamine (7.3 g, 1.0 M), *N,N*-diisopropylethylamine (17 g, 1.7 M) and B_{12}–Mg^{2+}/TiO_2 (500 mg, [B_{12}] = 6.1×10^{-6} M) was stirred in 100 mL of CH_3CN under air at room temperature. The suspension was stirred for 80 h during visible light irradiation by blue LED (λ= 405 nm). The reaction progress was monitored by GC-MS. After saturating the **1b** formation, light irradiation was stopped and then the B_{12}–Mg^{2+}/TiO_2 was separated by a filter. The resulting solution was extracted by 100 mL of CH_2Cl_2, and organic layer was washed with 10% citric acid aq solution, saturated sodium bicarbonate aq solution, and distilled water. After drying over anhydrous sodium sulfate, the solution was concentrated by an evaporator. The product was isolated by chromatography (Silica Gel 60 N, spherical, neutral) using *n*-hexane and ethyl acetate as eluent. The solution was concentrated by an evaporator to obtain light-yellow liquid. The product was characterized by ^{1}H NMR, ^{13}C NMR and GC-MS.

Healing treatment and recyclability test of the hybrid photocatalyst. The recyclability test of B_{12}–Mg^{2+}/TiO_2 is performed based on illustrated procedure in Figure S4-3. After first **3** synthesis, the suspension was centrifuged to obtain yellow powder. This powder, recycling Mg^{2+}/TiO_2, was washed with CH_3OH for 3 times and dried under vacuum. 150 mg of the recycling Mg^{2+}/TiO_2 was suspended in 1.5 mL of the methanol solution containing the B_{12} complex (5.5 mg 3.9×10^{-3} mmol) and the suspension was stirred for 24 hours in dark. The mixture was centrifuged and washed three times with CH_3OH. The obtained violet powder was dried for 3 hours under vacuum at room temperature. Yield is 139 mg. The second **3** synthesis was carried out using the violet powder, healed B_{12}–Mg^{2+}/TiO_2 as photocatalyst under the optimized conditions.

Visible light-driven carbamoyl fluoride synthesis from CBr_3F. A mixture of tribromofluoromethane (16 mg, 6.0×10^{-3} M), dibutylamine (78 mg, 6.0×10^{-2} M), *N,N*-diisopropylethylamine (129 mg, 1.0×10^{-2} M) and B_{12}–Mg^{2+}/TiO_2 (20 mg, [$\mathbf{B_{12}}$] = 6.1×10^{-6} M) was stirred in 10 mL of CH_3CN under air at room temperature. The suspension was stirred for 6 hours during visible light irradiation ($\lambda \geqq 420$ nm) and then the B_{12}–Mg^{2+}/TiO_2 was separated by a filter. The resulting solution was analyzed by GC-MS and yields of the products were calculated by comparison to the ratio of the peak area using diphenyl as an internal standard.

Reductive formation of the Co(I) species of the B_{12} complex on the TiO_2 surface under visible light irradiation. The reductive formation of the Co(I) species of the B_{12} complex on the TiO_2 surface under visible light irradiation was investigated by DR-UV-vis spectroscopy. A 5.0 mg of B_{12}–Mg^{2+}/TiO_2 was suspended in 5 mL of a CH_3CN solution in the presence of triethylamine (51 mg 1.0×10^{-1} M) as the sacrificial reductant. The suspension was monitored by DR-UV-vis spectroscopy during visible light irradiation by a 200 W tungsten lamp equipped with a 420 nm cut-off filter and a heat cut-off filter under a N_2 atmosphere.

Reactivity of Co(I) species toward CCl_4. The reactivity of Co(I) species of the B_{12} complex was investigated by DR-UV-vis spectroscopy. After reductive formation of the Co(I) species on the TiO2 surface under visible light irradiation, excess CCl_4 was added to this suspension at room temperature under completely dark condition. This suspension was carefully measured by DR-UV-vis spectroscopy. The suspension was also measured by DR-UV-vis spectroscopy after visible light irradiation at room temperature under air.

CVs. CVs were obtained using a BAS CV 50 W electrochemical analyzer. A three-electrode cell equipped with a 3.0-mm diameter glassy carbon rod and 1.6-mm diameter platinum wire as the working and counter electrode were used, respectively. An Ag/AgCl (3.0 M NaCl) was used as a reference electrode. Nonaqueous acetonitrile solutions containing **[Cob(II)7C_1ester]ClO_4** (**C2**) (1.0×10^{-3} M) and *n*-Bu_4NClO_4 (1.0×10^{-1} M) were deaerated prior to any measurement, and the inside of the cell was maintained under N_2 throughout the measurement. The CVs of CCl_4 and **C2** with CCl_4 in CH_3CN were also measured under N_2 with same conditions. The $E_{1/2}$ value of ferrocene/ferrocenium (Fc/Fc^+) was +0.44 V *vs.* Ag/AgCl with this setup.

Femtosecond time-resolved diffuse reflectance spectroscopy (fs-TDRs). The femtosecond time-resolved diffuse reflectance spectra were measured by the pump and

probe method using a regeneratively amplified titanium sapphire laser (Spectra-Physics, Spitfire Pro F, 1 kHz) pumped by a Nd:YLF laser (Spectra-Physics, Empower 15). The seed pulse was generated by a titanium sapphire laser (Spectra-Physics, Mai Tai VFSJW; fwhm 80 fs). The second harmonic generation of the fundamental light (405 nm, 3 μJ pulse-1, Spectra-Physics, OPA-800CF-1) was used as the excitation pulse. A white light continuum pulse, which was generated by focusing the residual of the fundamental light on a sapphire crystal after the computer controlled optical delay, was divided into two parts and used as the probe and the reference lights, of which the latter was used to compensate the laser fluctuation. The powder samples were dispersed into ethanol to be ink and then spread on cleaned glass cover slip. Both probe and reference lights were directed to the sample powder coated on the glass substrate, and the reflected lights were detected by a linear InGaAs array detector equipped with the polychromator (Solar, MS3504). The pump pulse was chopped by the mechanical chopper synchronized to one-half of the laser repetition rate, resulting in a pair of spectra with and without the pump, from which the absorption change (% absorption) induced by the pump pulse was estimated.

Computational Method. All density-functional-theory (DFT) calculations were performed using the Gaussian 16 program package.[2] For the calculations of the reactions involving with the Co complex (Figure 5), the TPSS functional was used.[3] We employed the (15s11p6d) primitive set of Wachters–Hay supplemented with one polarization f-function (α = 1.17)[4] for the Co atoms while employing the D95** basis sets[5] for the H, C, N, O, and Cl atoms, respectively. Additionally, we incorporated the Gibbs free energy correction (T = 298.15 K) and Grimme's dispersion correction (D3).[6] To account for implicit solvent effects, acetonitrile (ε = 35.688) was included using the polarizable continuum model (PCM).[7] For the other organic reactions in Figure S5, geometry optimization and energy calculations were performed with the B3LYP/6-31G* level of theory under vacuum condition.[8,9]

Vibration frequencies were systematically calculated to validate that each optimized geometry corresponded either to a local minimum, characterized by the absence of imaginary frequencies, or to a saddle point with only one imaginary frequency on the potential energy surface. The open-shell singlet, doublet, triplet, and quartet states were calculated by broken-symmetry approach.

3. Characterization of Hybrid Catalysts

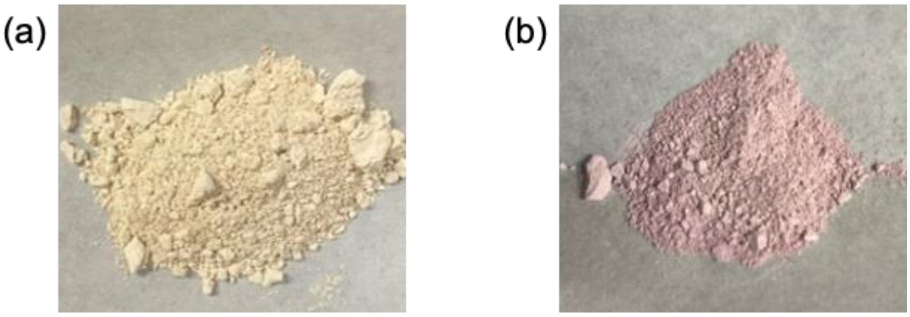

Figure S3-1. (a) Mg^{2+}/TiO_2, (b) B_{12}–Mg^{2+}/TiO_2.

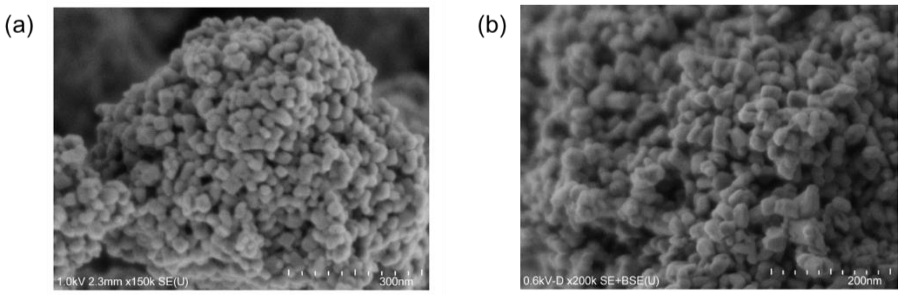

Figure S3-2. SEM images of (a) Mg^{2+}/TiO_2 and (b) B_{12}–Mg^{2+}/TiO_2.

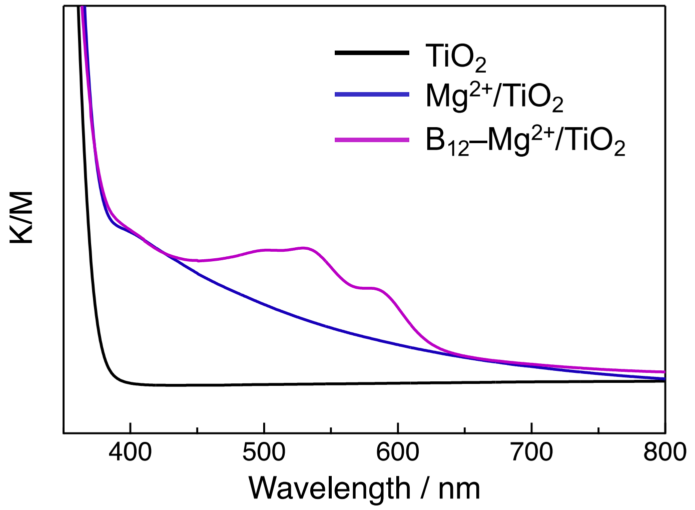

Figure S3-3. DR-UV-vis spectra of bare TiO_2 (black line), Mg^{2+}/TiO_2 (blue line), and B_{12}–Mg^{2+}/TiO_2 (magenta line).

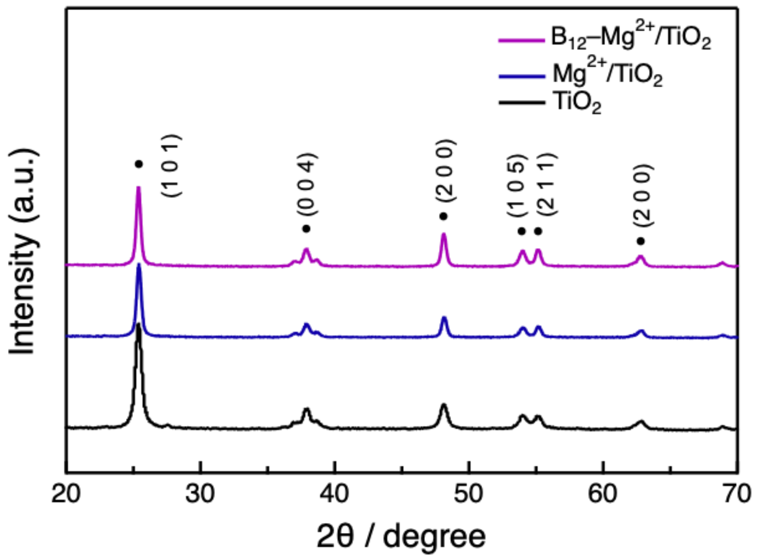

Figure S3-4. PXRD patterns of (a) bare TiO_2 (black line), Mg^{2+}/TiO_2 (blue line), and B_{12}–Mg^{2+}/TiO_2 (magenta line).

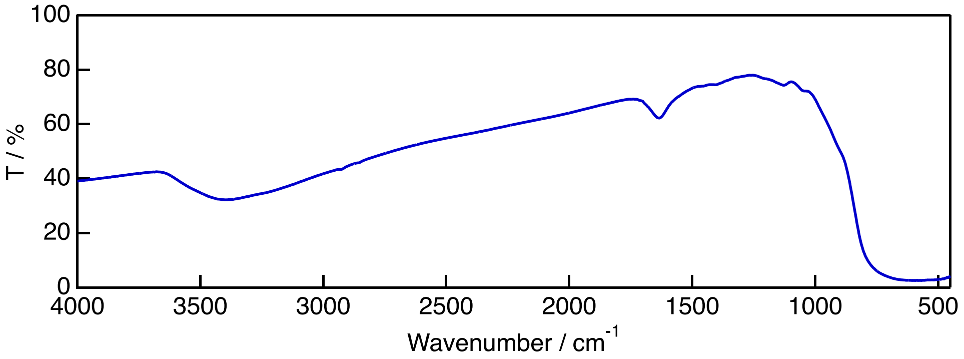

Figure S3-5. IR spectrum of Mg^{2+}/TiO_2 (KBr disk).

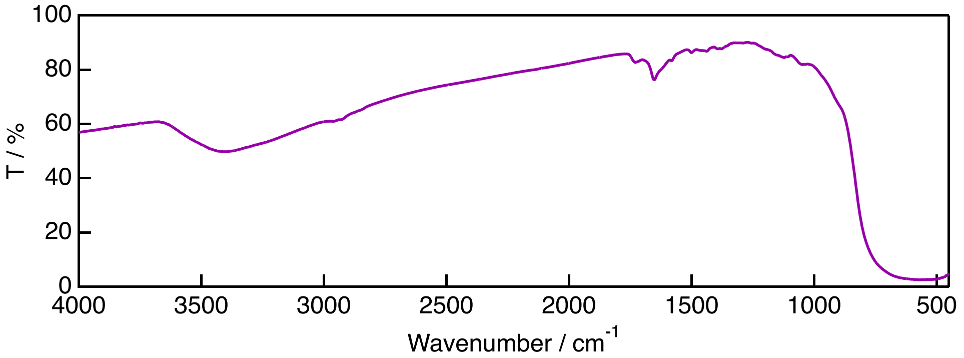

Figure S3-6. IR spectrum of B_{12}–Mg^{2+}/TiO_2 (KBr disk).

4. Photocatalytic reactions

4-1. Reaction optimizations

Table S4-1. Optimization of B_{12}–Mg^{2+}/TiO_2.

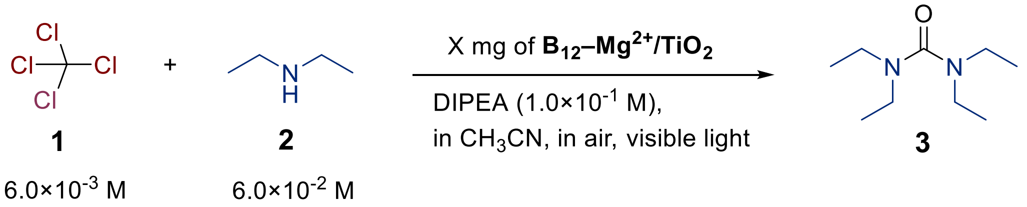

Entry	B_{12}–Mg^{2+}/TiO_2 Amount (mg)	Yield (%)
1	5	33
2	10	45
3	20	89
4	40	68

Reaction conditions: [**1**] = 6.0×10^{-3} M, [DIPEA] = 1.0×10^{-1} M, [**2**] = 6.0×10^{-2} M, B_{12}–Mg^{2+}/TiO_2 = X mg, solvent: 10 mL of CH_3CN, light source: 200W tungsten lamp with 42L cut-off filter ($\lambda \geq 420$ nm) and a heat cut-off filter (Sigma Koki, 30 H), reaction time: 6 hours.

Table S4-2. Optimization of **2** and DIPEA.

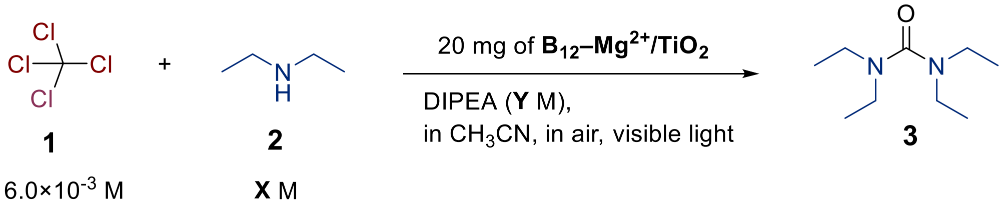

Entry	**2** (M)	DIPEA (M)	Yield (%)
1	1.2×10^{-1}	1.0×10^{-1}	75
2	6.0×10^{-2}	1.0×10^{-1}	89
3	3.0×10^{-2}	1.0×10^{-1}	30
4	6.0×10^{-2}	5.0×10^{-2}	71

Reaction conditions: [**1**] = 6.0×10^{-3} M, [DIPEA] = Y M, [**2**] = X M, B_{12}–Mg^{2+}/TiO_2 = 20 mg ([B_{12}] = 1.2×10^{-5} M), solvent: 10 mL of CH_3CN, light source: 200W tungsten lamp with 42L cut-off filter ($\lambda \geq 420$ nm) and a heat cut-off filter (Sigma Koki, 30 H), reaction time: 6 hours.

Table S4-3. Optimization of reaction time.

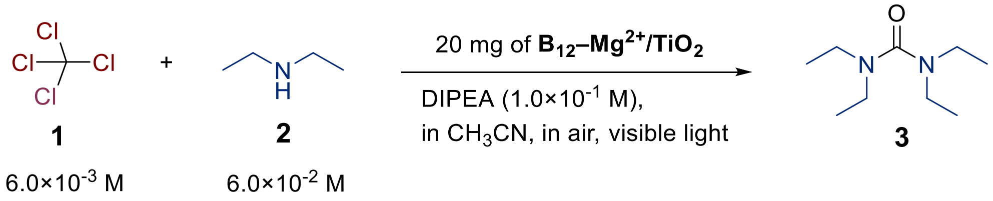

Entry	Reaction time (h)	Yield (%)
1	0	0
2	2	18
3	4	58
4	6	89

Reaction conditions: [**1**] = 6.0×10^{-3} M, [DIPEA] = 1.0×10^{-1} M, [**2**] = 6.0×10^{-2} M, B_{12}–Mg^{2+}/TiO_2 = 20 mg ([B_{12}] = 1.2×10^{-5} M), solvent: 10 mL of CH_3CN, light source: 200W tungsten lamp with 42L cut-off filter ($\lambda \geq 420$ nm) and a heat cut-off filter (Sigma Koki, 30 H), reaction time: X hours.

Table S4-4. Optimization of preferable reaction condition for high selective **16** synthesis.

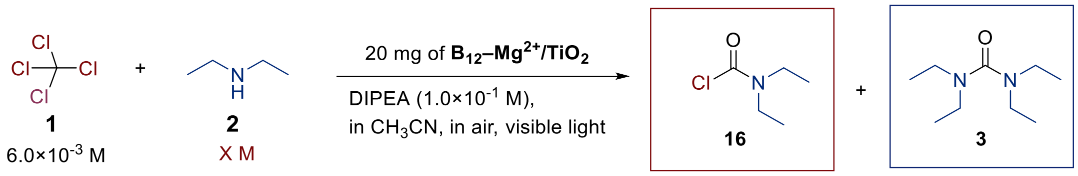

Entry	[$NHEt_2$] / M	Time / h	Yield of **16** /%	Yield of **3** /%
1	6.0×10^{-2}	6	9	89
2	3.0×10^{-2}	6	48	39
3	1.5×10^{-2}	6	72	24
4	7.5×10^{-3}	6	53	1
5	7.5×10^{-3}	3	68	< 1

[a]Reaction conditions: [**1**] = 6.0×10^{-3} M, [DIPEA] = 1.0×10^{-1} M, [**2**] = X M, B_{12}–Mg^{2+}/TiO_2 = 20 mg ([B_{12}] = 1.2×10^{-5} M), solvent: 10 mL of CH_3CN, light source: 200W tungsten lamp with 42L cut-off filter ($\lambda \geq 420$ nm) and a heat cut-off filter (Sigma Koki, 30 H), reaction time: Y hours.

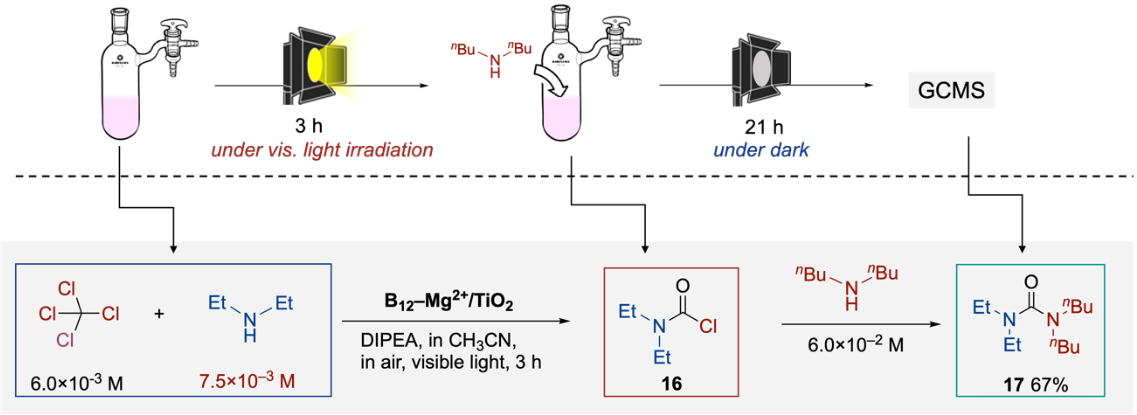

Figure S4-1. The unsymmetric urea (**17**) synthesis.

4-2. Gram-scale synthesis of 1,1,3,3-tetrathylurea from CCl_4.

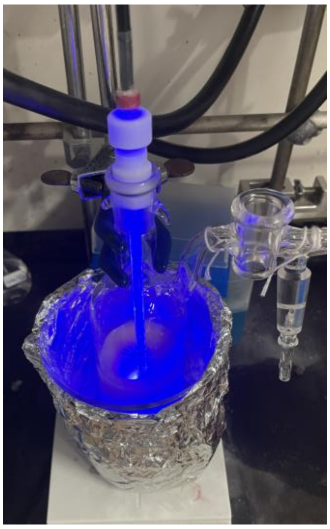

Figure S4-2. Gram-scale synthesis of **3**.

Scheme S4-1. Gram-scale synthesis of **3**.

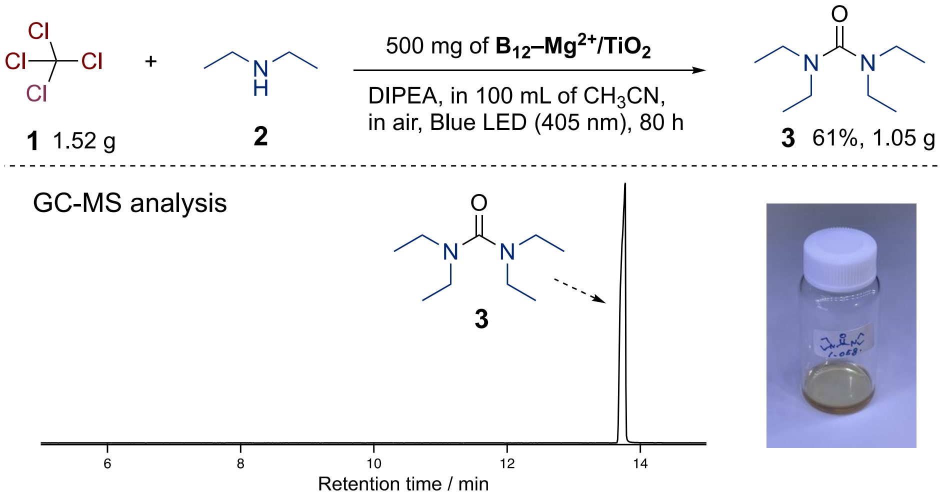

4-3. Healing treatment and recyclability test of the hybrid photocatalyst.

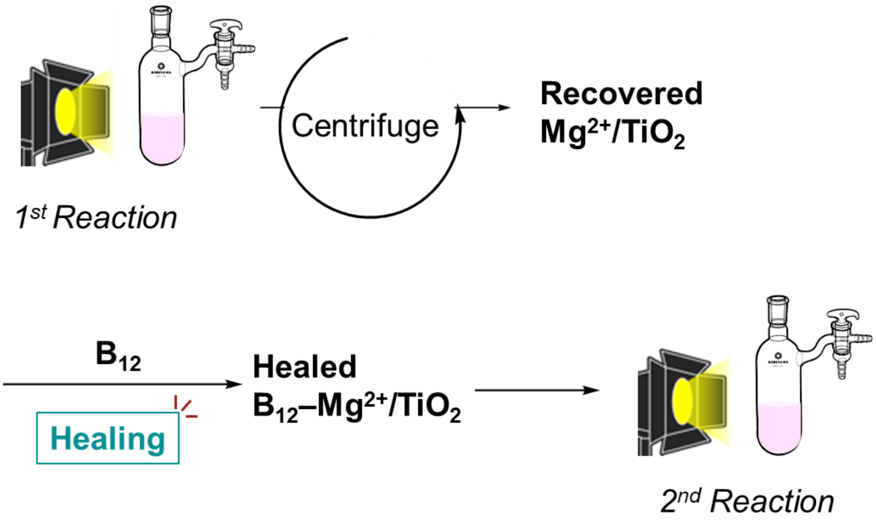

Figure S4-3. Process of recycling test.

4-4. Mechanistic studies.

Scheme 4-2. Mechanistic studies.

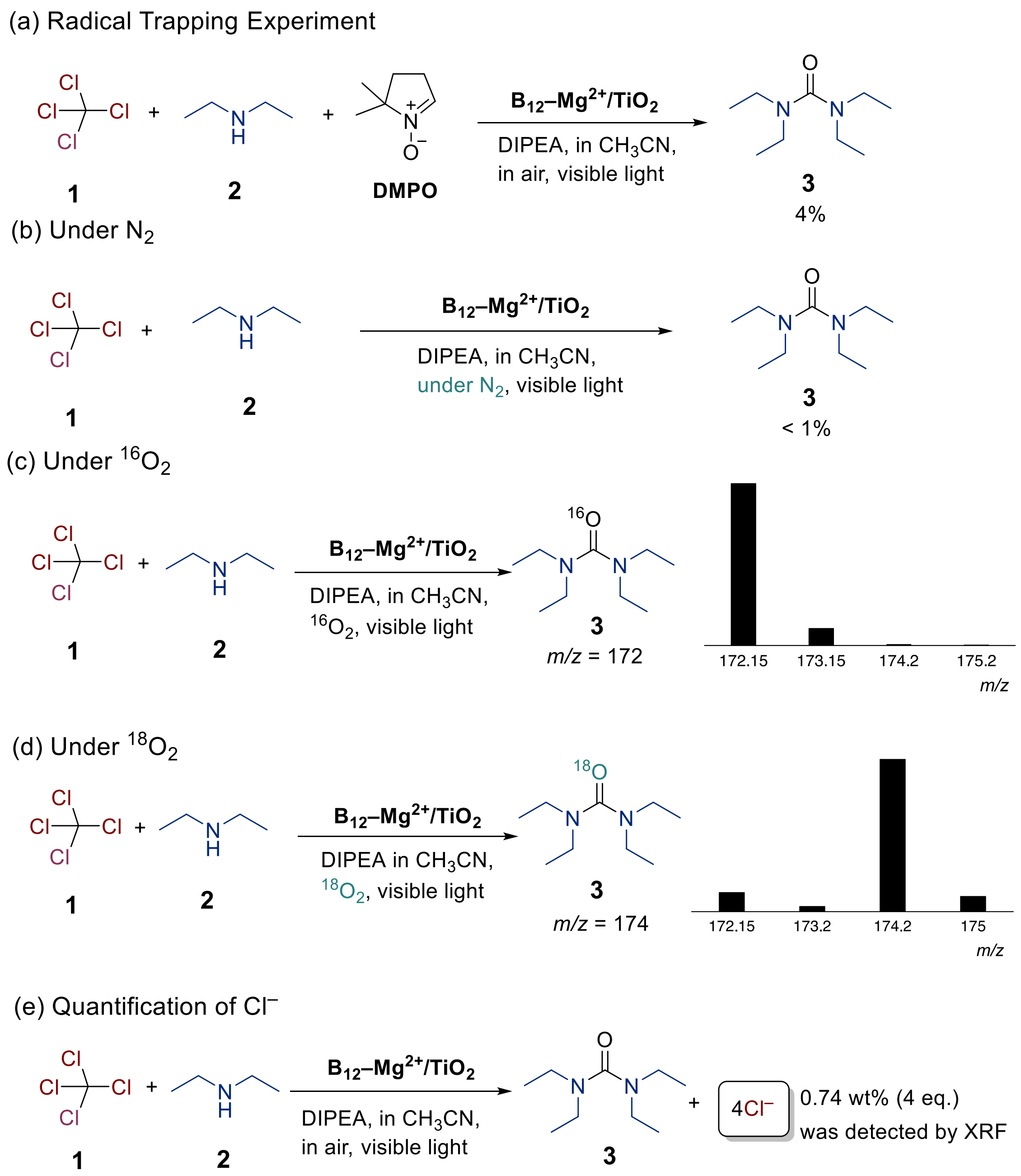

4-5. Optimization of photocatalytic carbonylation with CBr_4.

Table S4-5. Optimization of CBr_4 and B_{12}–Mg^{2+}/TiO_2.

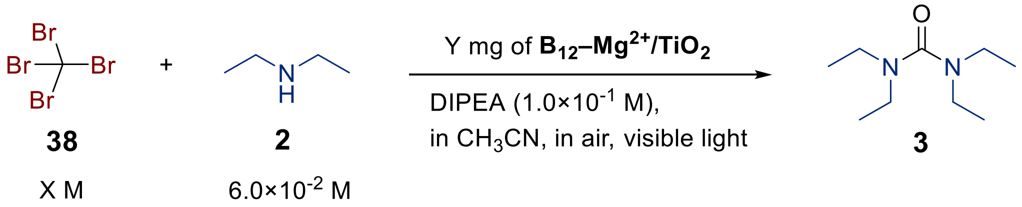

Entry	CBr_4 (M)	B_{12}–Mg^{2+}/TiO_2 (mg)	Yield (%)
1	6.0×10^{-3}	20	40
2	3.0×10^{-3}	20	55
3	1.5×10^{-3}	20	57
4	1.5×10^{-3}	10	52

Reaction conditions: [**38**] = X M, [DIPEA] = 1.0×10^{-1} M, [**2**] = 6.0×10^{-2} M, B_{12}–Mg^{2+}/TiO_2 = Y mg, solvent: 10 mL of CH_3CN, light source: 200W tungsten lamp with 42L cut-off filter ($\lambda \geq$ 420 nm) and a heat cut-off filter (Sigma Koki, 30 H), reaction time: 2 hours.

Table S4-6. Optimization of **2** and DIPEA.

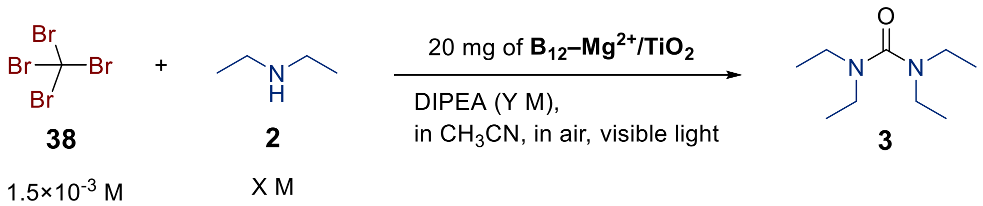

Entry	**2** (M)	DIPEA (M)	Yield (%)
1	6.0×10^{-2}	1.0×10^{-1}	57
2	3.0×10^{-2}	1.0×10^{-1}	59
3	1.5×10^{-2}	1.0×10^{-1}	60
4	6.0×10^{-3}	1.0×10^{-1}	42
6	1.5×10^{-2}	5.0×10^{-2}	66
7	1.5×10^{-2}	1.0×10^{-2}	67

Reaction conditions: [**38**] = 1.5×10^{-3} M, [DIPEA] = Y M, [**2**] = X M, B_{12}–Mg^{2+}/TiO_2 = Y mg, solvent: 10 mL of CH_3CN, light source: 200W tungsten lamp with 42L cut-off filter ($\lambda \geq$ 420 nm) and a heat cut-off filter (Sigma Koki, 30 H), reaction time: 2 hours.

Table S4-7. Control experiments for visible light-driven carbamoyl fluoride synthesis from $CFBr_3$.[a]

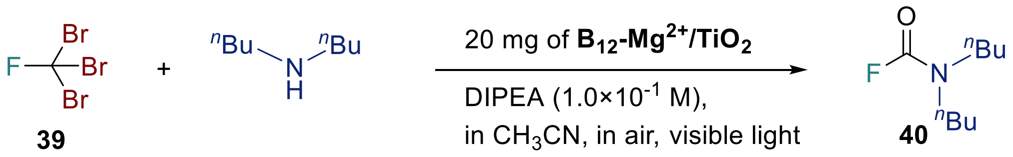

Entry	Change from standard condition	Yield (%)[b]	TON[c]
1	None	94	468
2	In dark	0	0
3	Without catalyst	6	–
4	Without DIPEA	13	63

[a]Reaction conditions: [**39**] = 6.0×10^{-3} M, [DIPEA] = 1.0×10^{-1} M, [$NHBu_2$] = 6.0×10^{-2} M, B_{12}–Mg^{2+}/TiO_2 = 20 mg ([B_{12}] = 1.2×10^{-5} M), solvent: 10 mL of CH_3CN, light source: 200W tungsten lamp with 42L cut-off filter (λ ≥ 420 nm) and a heat cut-off filter (Sigma Koki, 30 H). Reaction time: 6 hours.
[b]Yields based on the initial concentration of the substrate.
[c]TON based on B_{12} catalyst.

5. Mechanistic Studies

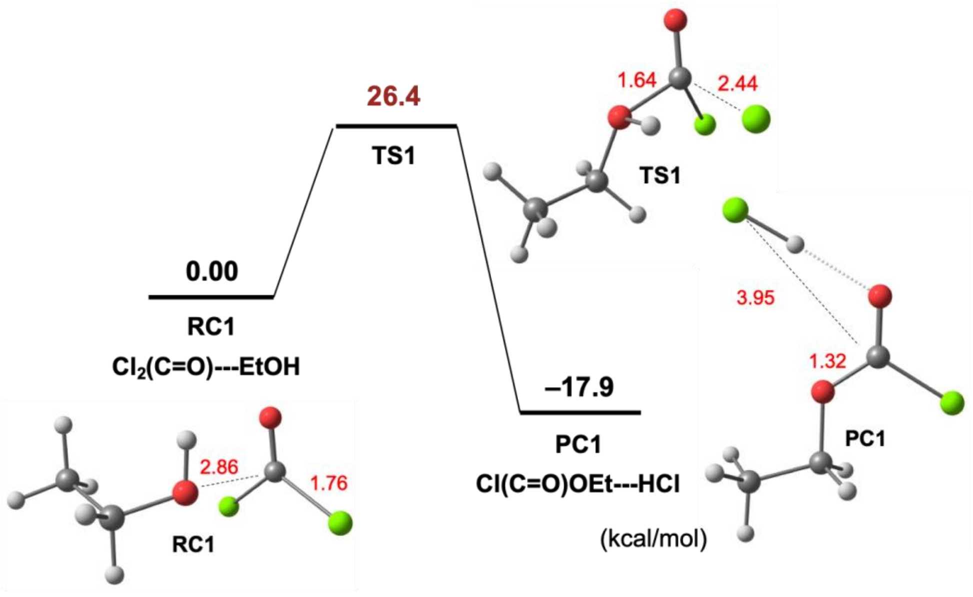

Figure S5-1. Energy diagram for the reaction of phosgene and ethanol.

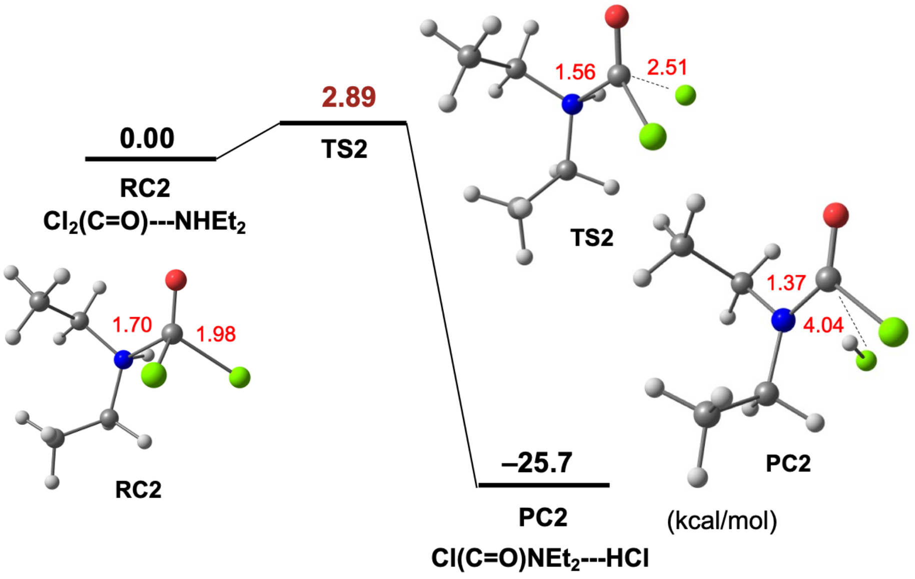

Figure S5-2. Energy diagram for the reaction of phosgene and diethylamine.

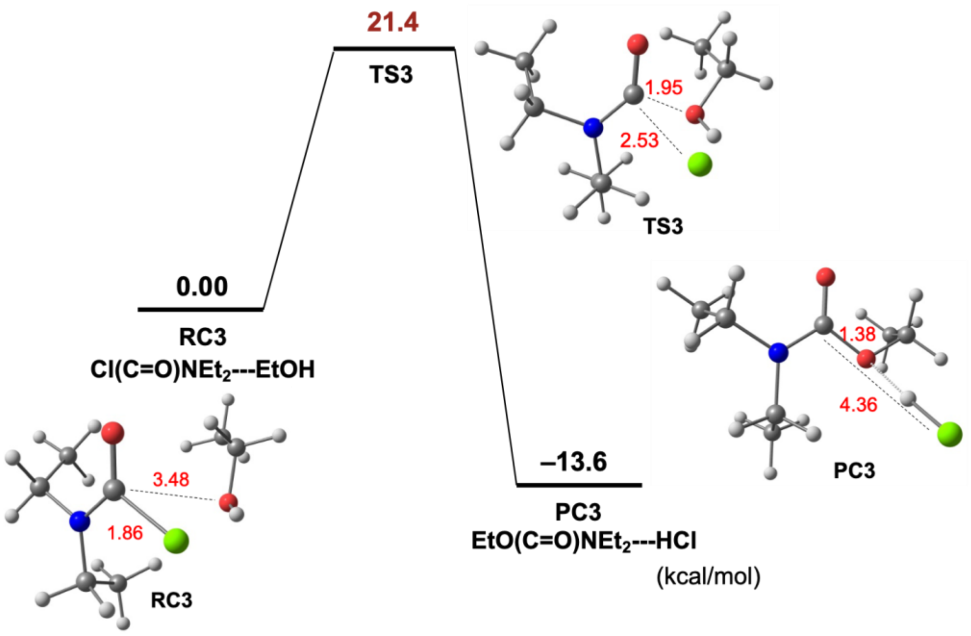

Figure S5-3. Energy diagram for the reaction of compound **16** and ethanol.

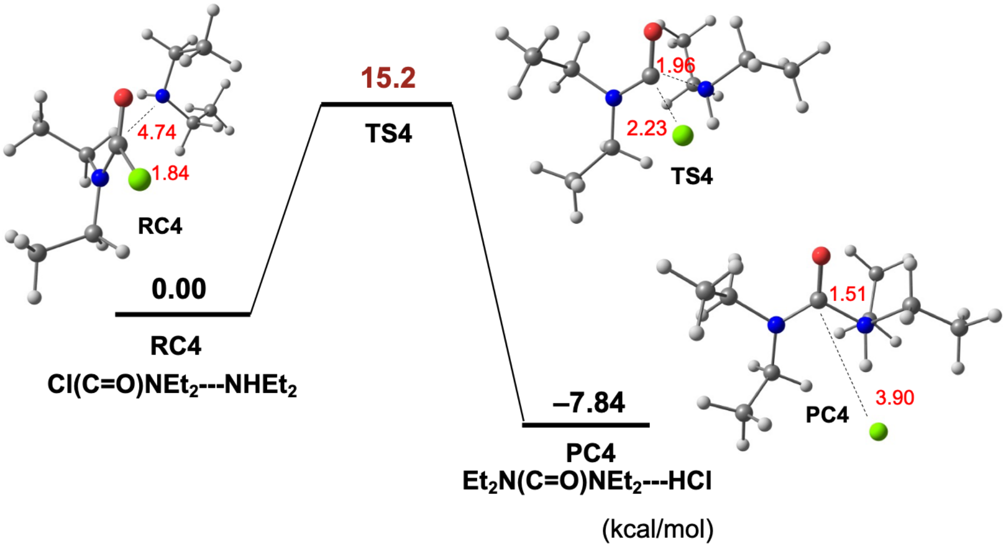

Figure S5-4. Energy diagram for the reaction of compound **16** and diethylamine.

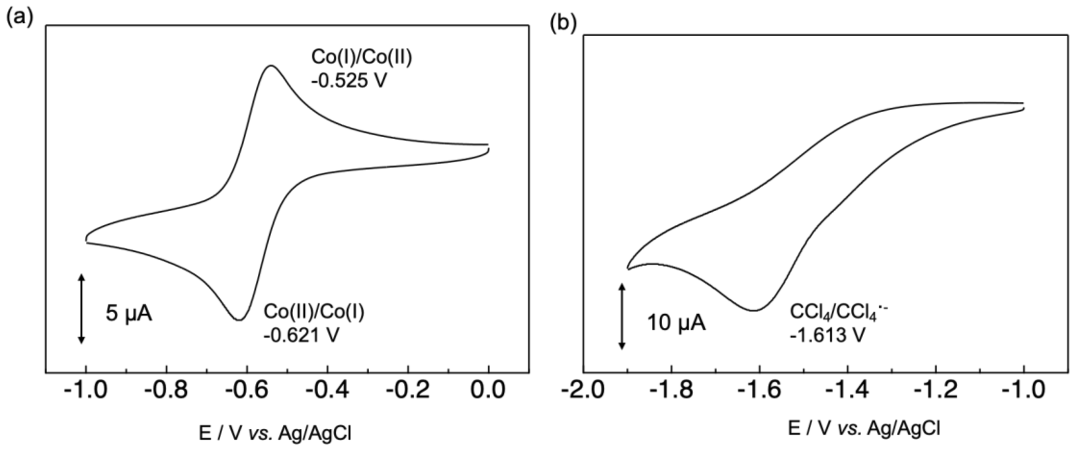

Figure S5-5. CVs of (a) **C2** and (b) CCl_4 in CH_3CN with 0.1 M of *n*-Bu_4NPF_6 as a supporting electrolyte. Scan rate: 0.1 V s^{-1}, WE: glassy carbon, CE: Pt, and RE: Ag/AgCl.

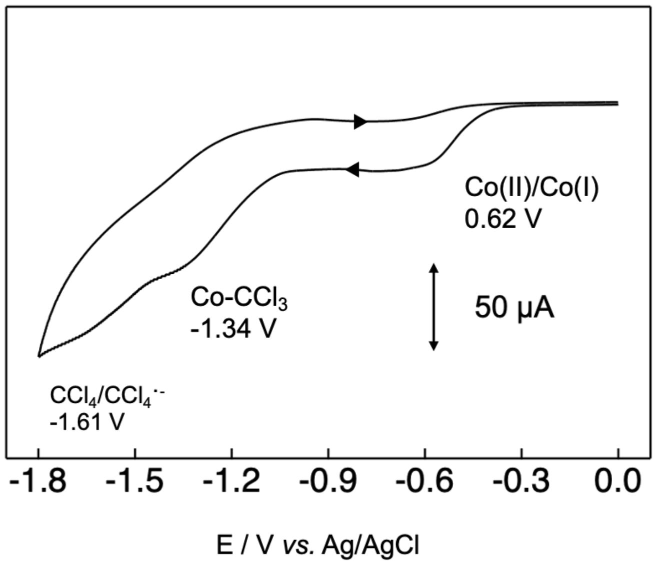

Figure S5-6. CVs of **C2** with CCl_4 in CH_3CN with 0.1 M of *n*-Bu_4NPF_6 as a supporting electrolyte. Scan rate: 0.1 V s^{-1}, WE: glassy carbon, CE: Pt, and RE: Ag/AgCl.

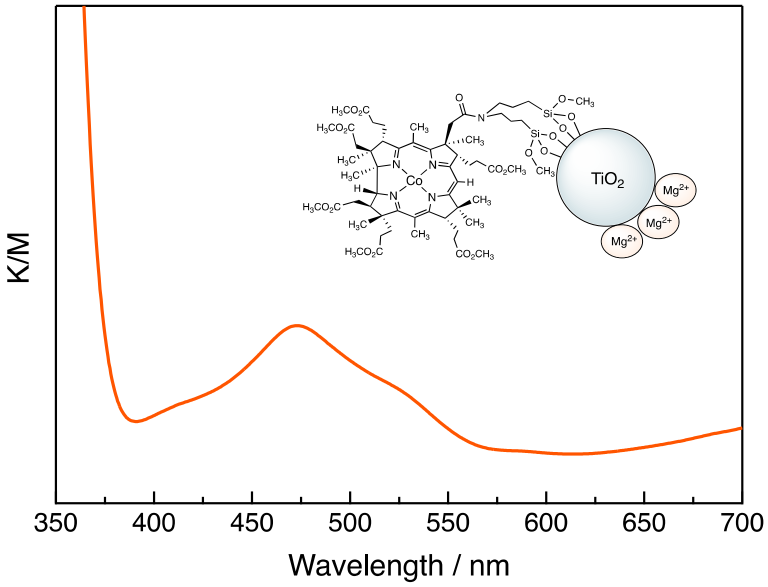

Figure S5-7. DR-UV-vis spectrum of Co(II) species of B_{12}–Mg^{2+}/TiO_2. Co(II) species of B_{12}–Mg^{2+}/TiO_2 was prepared by irradiation of UV light for 5 min in methanol and addition of air to resulting suspension.

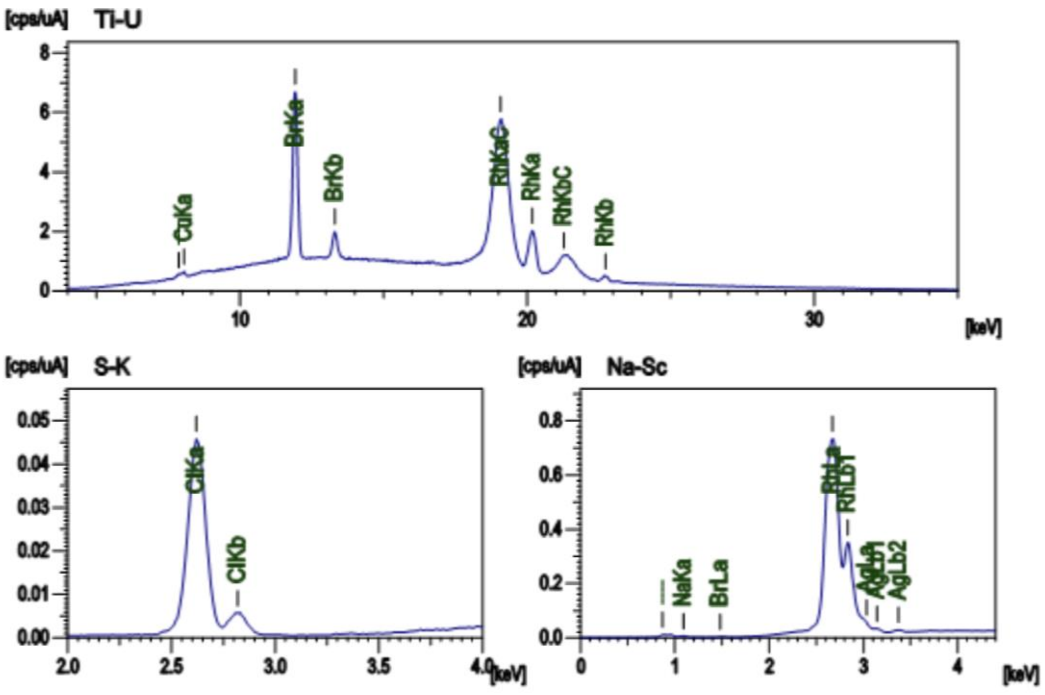

Figure S5-8. EDX analysis for aqueous solution after the reaction. 0.74 wt% of Cl was detected.

6. Products characterizations

Products characterizations

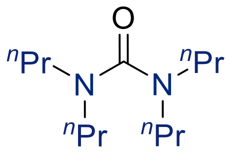

1,1,3,3-Tetrapropylurea (**4**)

Colorless oil (**4**) was obtained by general procedure.

^{1}H NMR (400 MHz, $CDCl_3$): δ 3.09-3.06 (m, 8H), 1.57-1.47 (m, 8H), 0.86 (t, *J* = 8.0Hz, 12H). **^{13}C NMR (101 MHz, $CDCl_3$)**: δ 165.6, 50.0, 21.2, 11.4. **IR (ATR)**: 2961, 2933, 2874, 1643, 1463, 1411, 1378, 1341, 1294, 1234 cm^{-1}.

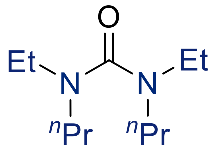

1,3-Diethyl-1,3-dipropylurea (**6**)

Colorless oil (**26**) was obtained by general procedure.

^{1}H NMR (400 MHz, $CDCl_3$): δ 3.17 (q, *J* = 6.7 Hz, 4H), 3.09-3.05 (m, 4H), 1.58-1.48 (m, 4H), 1.10 (t, *J* = 8.0 Hz, 6H), 0.87 (t, *J* = 8.0 Hz, 6H). **^{13}C NMR (101 MHz, $CDCl_3$)**: δ 165.3, 49.5, 42.9, 21.2, 13.21, 11.43. **IR (ATR)**: 2964, 2933, 2874, 1643, 1463, 1413, 1377, 1343, 1247 cm^{-1}.

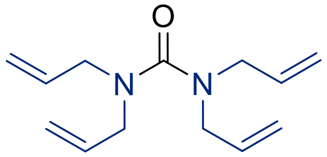

1,1,3,3-Tetraallylurea (**7**)

Colorless oil (**7**) was obtained by general procedure. Spectral data correlated with that previously reported in the literature.

^{1}H NMR (400 MHz, $CDCl_3$): δ 5.86-5.77(m, 4H), 5.19-5.14 (m, 8H), 3.75 (d, *J* = 4.0 Hz, 8H). **^{13}C NMR (101 MHz, $CDCl_3$)**: δ 164.4, 134.0, 117.0, 50.2. **IR (ATR)**: 3079, 3010, 2982, 2928, 2859, 1636, 1459, 1432, 1400, 1359, 1278, 1226 cm^{-1}.

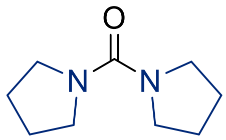

1,1'-Carbonyldipyrrolidine (**9**)

Yellow oil (**9**) was obtained by general procedure. Spectral data correlated with that previously reported in the literature.

^{1}H NMR (400 MHz, $CDCl_3$): δ 3.39-3.35 (m, 8H), 1.84-1.81 (m, 8H). **^{13}C NMR (101 MHz, $CDCl_3$)**: δ 161.4, 47.9, 25.5. **IR (ATR)**: 2964, 2871, 1610, 1404, 1337 cm^{-1}.

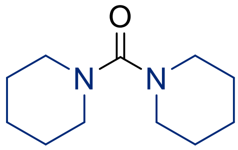

1,1'-Carbonyldipiperidine (**10**)

Colorless oil (**10**) was obtained by general procedure. Spectral data correlated with that previously reported in the literature.

^{1}H NMR (400 MHz, $CDCl_3$): δ 3.18-3.15 (m, 8H), 1.56-1.52 (m, 12H). **^{13}C NMR (101 MHz, $CDCl_3$)**: δ 164.8, 47.9, 25.8, 24.8. **IR (ATR)**: 2930, 2850, 1639, 1412, 1369, 1248, 1211 cm^{-1}.

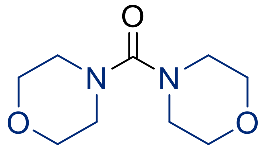

4,4'-Carbonyldimorpholine (**11**)

White solid (**11**) was obtained by general procedure. Spectral data correlated with that previously reported in the literature.

^{1}H NMR (400 MHz, $CDCl_3$): δ 3.68 (t, J = 6 Hz, 8H), 3.28 (t, J = 6 Hz, 8H). **^{13}C NMR (101 MHz, $CDCl_3$)**: δ 163.8, 66.6, 47.2. **IR (ATR)**: 3008, 2971, 2856, 2838, 2359, 2341 1640, 1460, 1437, 1403, 1357, 1327, 1309, 1272, 1262, 1234, 1217, 1207 cm^{-1}.

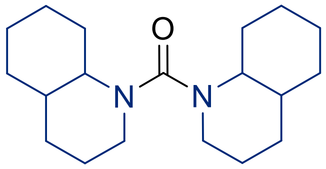

1,1’-Carbonyldecahydroquinoline (**12**)

Yellow oil (**12**) was obtained by general procedure.

^{1}H NMR (400 MHz, $CDCl_3$): δ 3.08-3.05 (m, 2H), 2.72-2.61 (m, 2H), 1.99-1.85 (m, 2H), 1.61-0.95 (m, 22H). **^{13}C NMR (101 MHz, $CDCl_3$)**: δ 165.6, 62.1, 60.8, 32.9, 32.8, 30.3, 30.1, 25.9, 25.9, 25.2, 24.7. **IR (ATR)**: 2920, 2850, 1646, 1446, 1406, 1352, 1314, 1284, 1264, 1242, 1222 cm^{-1}.

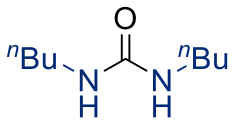

1,3-Dibutylurea (**13**)

White solid (**13**) was obtained by general procedure. Spectral data correlated with that previously reported in the literature.

^{1}H NMR (400 MHz, $CDCl_3$): δ 4.16 (bs, 2H), 3.16 (q, *J* = 6.7 Hz, 4H), 1.50-1.44 (m, 4H), 1.38-1.32 (m, 4H), 0.92 (t, *J* = 6.0 Hz, 6H). **^{13}C NMR (101 MHz, $CDCl_3$)**: δ 158.3, 40.3, 32.3, 20.0, 13.8. **IR (ATR)**: 3326, 2958, 2930, 2861, 1621, 1568, 1479, 1462, 1374, 1275, 1232 cm^{-1}.

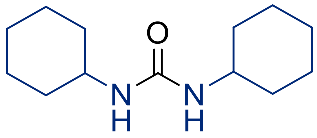

1,3-Dicyclohexylurea (**14**)

White solid (**14**) was obtained by general procedure. Spectral data correlated with that previously reported in the literature.

^{1}H NMR (400 MHz, $CDCl_3$): δ 4.05-4.03(br, 2H), 3.53-3.44(m, 2H), 1.97-1.91(m, 4H), 1.73-1.57(m, 6H), 1.41-1.30(m, 4H), 1.20-1.05(m, 6H). **^{13}C NMR (101 MHz, $CDCl_3$)**: δ156.7, 49.2, 34.0, 25.6, 24.9. **IR (ATR)**: 3322, 2926, 2848, 1622, 1568, 1536, 1436, 1310, 1270, 1242 cm^{-1}.

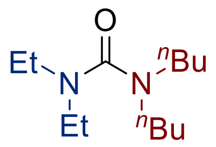

1,1-Dibutyl-3,3-diethylurea (**17**)

Yellow oil (**17**) was obtained by general procedure.

^{1}H NMR (400 MHz, CDCl$_3$): δ 3.18-3.09 (m, 8H), 1.52-1.44 (m, 4H), 1.28 (sext, *J* = 8 Hz, 4H), 1.10 (t, *J* = 8 Hz, 6H), 0.91 (t, *J* = 8.0 Hz, 6H). **^{13}C NMR (101 MHz, CDCl$_3$)**: δ 165.2, 47.8, 30.1, 20.2, 13.9, 13.2. **IR (ATR)**: 2959, 2931, 2873, 1643, 1459, 1413, 1376, 1281, 1257, 1233 cm^{-1}.

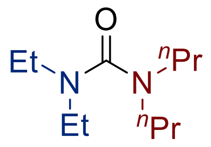

1,1-Diethyl-3,3-dipropylurea (**18**)

Yellow oil (**18**) was obtained by general procedure. Spectral data correlated with that previously reported in the literature.

^{1}H NMR (400 MHz, CDCl$_3$): δ 3.16 (q, *J* = 5.3 Hz, 4H), 3.10-3.07 (m, 4H), 1.57-1.48 (m, 4H), 1.10 (t, *J* = 8.0 Hz, 6H), 0.87 (t, *J* = 8.0 Hz, 6H). **^{13}C NMR (101 MHz, CDCl$_3$)**: δ 165.2, 49.9, 42.3, 21.1, 13.2, 11.4. **IR (ATR)**: 2964, 2933, 2874, 1643, 1477, 1463, 1413, 1377, 1247 cm^{-1}.

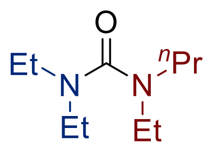

1,1,3-Triethyl-3-propylurea (**19**)

Colorless oil (**19**) was obtained by general procedure.

^{1}H NMR (400 MHz, CDCl$_3$): δ 3.20-3.13 (m, 6H), 3.09-3.05 (m, 2H)1.58-1.48 (m, 2H), 1.12-1.08 (m, 9H), 0.87 (t, *J* = 8.0 Hz, 3H). **^{13}C NMR (101 MHz, CDCl$_3$)**: δ 165.1, 49.4, 42.8, 42.3, 21.2, 13.2, 11.4. **IR (ATR)**: 2966, 2932, 2873, 1642, 1459, 1478, 1414, 1377, 1254, 1214 cm^{-1}.

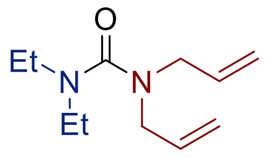

1,1-Diallyl-3,3-diethylurea (**20**)

Yellow oil (**20**) was obtained by general procedure. Spectral data correlated with that previously reported in the literature.

^{1}H NMR (400 MHz, $CDCl_3$): δ 5.88-5.78 (m, 2H), 5.20-5.14 (m, 4H), 3.72-3.70(m, 4H), 3.20 (q, *J* = 6.7 Hz, 4H), 1.11 (t, *J* = 8 Hz, 6H). **^{13}C NMR (101 MHz, $CDCl_3$)**: δ 164.7, 134.3, 116.9, 50.4, 42.0, 13.1. **IR (ATR)**: 3079, 2971, 2932, 2873, 1639, 1405, 1379, 1357, 1279, 1249 cm^{-1}.

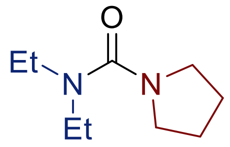

***N,N*-Diethylpyrrolidine-1-carboxamide** (**21**)

Colorless oil (**21**) was obtained by general procedure. Spectral data correlated with that previously reported in the literature.

^{1}H NMR (400 MHz, $CDCl_3$): δ 3.35-3.32 (m, 4H), 3.20 (q, *J* = 6.7 Hz, 4H), 1.84-1.80 (m, 4H), 1,13 (t, *J* = 8 Hz, 6H). **^{13}C NMR (101 MHz, $CDCl_3$)**: δ 163.0, 48.4, 41.6, 25.5, 13.4. **IR (ATR)**: 2968, 2934, 2872, 1623, 1477, 1415, 1376, 1346, 1322, 1296 cm^{-1}.

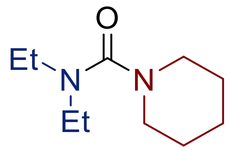

***N,N*-Diethylpiperidine-1-carboxamide** (**22**)

Colorless oil (**22**) was obtained by general procedure. Spectral data correlated with that previously reported in the literature.

^{1}H NMR (400 MHz, $CDCl_3$): δ 3.21-3.13 (m, 8H), 1.56-1.55 (m, 6H), 1.11 (t, *J* = 8 Hz, 6H). **^{13}C NMR (101 MHz, $CDCl_3$)**: δ 165.0, 48.1, 41.8, 25.7, 24.8, 13.2. **IR (ATR)**: 2964, 2933, 2900, 2874, 1642, 1477, 1460, 1413, 1378, 1244 cm^{-1}.

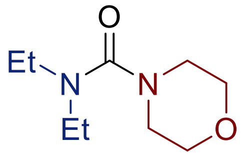

N,N-Diethylmorpholine-4-carboxamide (**23**)

Colorless oil (**23**) was obtained by general procedure. Spectral data correlated with that previously reported in the literature.

^{1}H NMR (400 MHz, $CDCl_3$): δ 3.21-3.13 (m, 8H), 1.56-1.55 (m, 6H), 1.11 (t, *J* = 8 Hz, 6H). **^{13}C NMR (101 MHz, $CDCl_3$)**: δ 164.5, 66.7, 47.6, 41.7, 13.2. **IR (ATR)**: 2968, 2932, 2894, 2852, 1636, 1457, 1412, 1381, 1360, 1299, 1251, 1215 cm^{-1}.

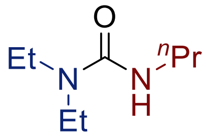

1,1-diethyl-3-propylurea (**24**)

Colorless oil (**24**) was obtained by general procedure.

^{1}H NMR (400 MHz, $CDCl_3$): δ 4.29 (bs, 1H), 3.28-3.19 (m, 6H), 1.53 (sext, *J* = 7.2 Hz, 2H), 1.14 (t, *J* = 8 Hz, 6H), 0.92 (t, *J* = 8 Hz, 3H). **^{13}C NMR (101 MHz, $CDCl_3$)**: δ 157.3, 42.5, 41.1, 23.6, 13.8, 11.4. **IR (ATR)**: 3346, 2963, 2932, 2874, 1623, 1530, 1495, 1457, 1405, 1377, 1275, 1225 cm^{-1}.

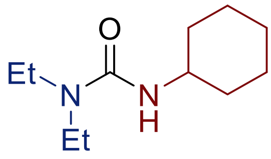

3-Cyclohexyl-1,1-diethylurea (**25**)

White solid (**25**) was obtained by general procedure.

^{1}H NMR (400 MHz, $CDCl_3$): δ 4.11 (bs, 1H), 3.71-3.63 (m, 1H), 3.24 (q, *J* = 8 Hz, 4H), 1.98-1.93 (m, 2H), 1.72-1.59 (m, 3H), 1.43-1.32 (m, 2H), 1.15-1.05(m, 9H). **^{13}C NMR (101 MHz, $CDCl_3$)**: δ 156.6, 49.2, 41.1, 34.1, 25.7, 25.1, 13.8. **IR (ATR)**: 3339, 2972, 2927, 2854, 1617, 1527, 1492, 1450, 1408, 1376, 1361, 1310, 1279, 1252 cm^{-1}.

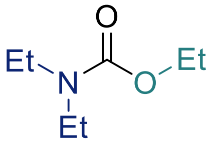

Ethyl *N,N*-diethylcarbamate (**26**)

Yellow oil (**26**) was obtained by general procedure. Spectral data correlated with that previously reported in the literature.

^{1}H NMR (400 MHz, CDCl$_3$): δ 4.13 (q, *J* = 6.7 Hz, 2H), 3.27 (br, 4H), 1.25 (q, *J* = 8.0 Hz, 3H), 1.11 (q, *J* = 8.0 Hz, 6 H). **^{13}C NMR (101 MHz, CDCl$_3$)**: δ 156.1, 60.8, 41.5, 41.3, 14.7, 13.9, 13.6. **IR (ATR)**: 2976, 2935, 2877, 1693, 1478, 1459, 1425, 1380, 1316, 1271, 1227 cm^{-1}.

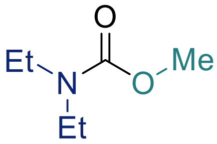

Methyl *N,N*- diethylcarbamate (**27**)

Yellow oil (**27**) was obtained by general procedure. Spectral data correlated with that previously reported in the literature.

^{1}H NMR (400 MHz, CDCl$_3$): δ 3.69 (s, 3H), 3.27 (bs, 4H), 1.11 (t, *J* = 8.0 Hz, 6H). **^{13}C NMR (101 MHz, CDCl$_3$)**: δ 156.5, 52.2, 41.6, 41.3, 14.0, 13.6. **IR (ATR)**: 2974, 2936, 2878, 1696, 1480, 1440, 1408, 1380, 1316, 1274, 1226 cm^{-1}.

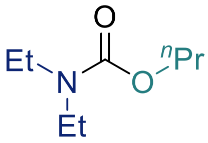

Propyl *N,N*-diethylcarbamate (**28**)

Yellow oil (**28**) was obtained by general procedure. Spectral data correlated with that previously reported in the literature.

^{1}H NMR (400 MHz, CDCl$_3$): δ 4.03 (t, *J* = 6.7 Hz, 2H), 3.27 (bs, 4H), 1.70-1.61 (m, 2H), 1.12 (t, *J* = 8.0 Hz, 6H), 0.95 (t, *J* = 8.0 Hz, 3H). **^{13}C NMR (101 MHz, CDCl$_3$)**: δ156.2, 66.5, 41.6, 41.2, 22.4, 14.0, 13.6, 10.5. **IR (ATR)**: 2971, 2936, 2879, 1694, 1479, 1459, 1425, 1380, 1316, 1271, 1227 cm^{-1}.

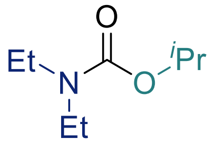

Isopropyl *N,N*-diethylcarbamate (**29**)

Yellow oil (**29**) was obtained by general procedure. Spectral data correlated with that previously reported in the literature.

^{1}H NMR (400 MHz, $CDCl_3$): δ 4.97-4.88 (m, 1H), 3.26 (bs, 4H), 1.24 (d, *J* = 4.0 Hz, 6H), 1.11 (t, *J* = 8.0 Hz, 6H). **^{13}C NMR (101 MHz, $CDCl_3$)**: δ 155.7, 67.9, 41.3, 22.3, 13.9. **IR (ATR)**: 2978, 2936, 2878, 1791, 1738, 1690, 1477, 1459, 1422, 1379, 1317, 1272, 1228, 1214 cm^{-1}.

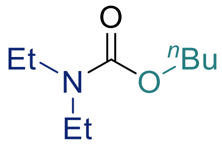

Butyl *N,N*-diethylcarbamate (**30**)

Colorless oil (**30**) was obtained by general procedure. Spectral data correlated with that previously reported in the literature.

^{1}H NMR (400 MHz, $CDCl_3$): δ 4.07 (t, *J* = 6.6 Hz, 2H), 3.27 (bs, 4H), 1.65-1.60 (m, 2H), 1.44-1.35 (dd, *J* = 15.1, 7.1 Hz, 2H), 1.11 (t, *J* = 7.1 Hz, 6H), 0.94 (t, *J* = 8.0 Hz, 3H). **^{13}C NMR (101 MHz, $CDCl_3$)**: δ 156.2, 64.8, 41.5, 41.1, 31.2, 19.2, 13.8. **IR (ATR)**: 2962, 2934, 2875, 1695, 1477, 1459, 1424, 1379, 1316, 1271, 1227 cm^{-1}.

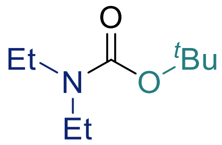

Tert-butyl *N,N*-diethylcarbamate (31)

Colorless oil (**31**) was obtained by general procedure. Spectral data correlated with that previously reported in the literature.

^{1}H NMR (400 MHz, $CDCl_3$): 3.23 (bs, 4H), 1.46 (s, 9H), 1.10 (t, *J* = 6 Hz, 6H). **^{13}C NMR (101 MHz, $CDCl_3$)**: δ 155.3, 78.9, 41.3, 28.5, 13.7. **IR (ATR)**: 2973, 2932, 2876, 1688, 1479, 1456, 1416, 1390, 1378, 1364, 1315, 1281, 1253, 1226 cm^{-1}.

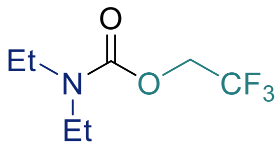

Trifluoromethyl *N,N*-diethylcarbamate (32)

Colorless oil (**32**) was obtained by general procedure.

^{1}H NMR (400 MHz, $CDCl_3$): 4.48 (q, J = 9.33 Hz, 2H), 3.36-3.26 (m, 4H), 1.17-1.13 (m, 6H). **^{13}C NMR (101 MHz, $CDCl_3$)**:153.9, 123.3 (q, $J_{C\text{-}F}$ = 278 Hz), 61.1 (q, $J_{C\text{-}F}$ = 36 Hz), 42.0 (d, $J_{C\text{-}F}$ = 84 Hz), 13.6 (d, $J_{C\text{-}F}$ = 59 Hz). δ **IR (ATR)**: 2977, 2938, 2879, 1713, 1479, 1460, 1430, 1382, 1368, 1351, 1290, 1261, 1225 cm^{-1}.

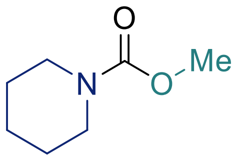

Methyl piperidine-1-carboxylate (**33**)

Yellow oil (**33**) was obtained by general procedure. Spectral data correlated with that previously reported in the literature.

^{1}H NMR (400 MHz, $CDCl_3$): δ 3.68 (s, 3H), 3.42-3.40 (m, 4H), 1.57 (m, 2H), 1.53-1.52 (m, 4H). **^{13}C NMR (101 MHz, $CDCl_3$)**: δ 156.0, 52.4, 44.7, 25.6, 24.3. **IR (ATR)**: 2935, 2855, 1696, 1471, 1443, 1407, 1372, 1352, 1260, 1234 cm^{-1}.

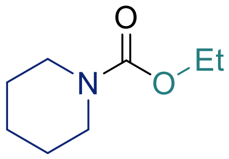

Ethyl piperidine-1-carboxylate (**34**)

Yellow oil (**34**) was obtained by general procedure. Spectral data correlated with that previously reported in the literature.

^{1}H NMR (400 MHz, $CDCl_3$): δ 4.12 (q, J = 8 Hz, 2H), 3.42-3.39 (m, 4H), 1.58-1.52 (m, 6H), 1.25 (t, J = 6.0 Hz, 3H). **^{13}C NMR (101 MHz, $CDCl_3$)**: δ 155.6, 61.0, 44.7, 25.6, 24.4, 14.7. **IR (ATR)**: 2981, 2934, 2855, 1693, 1471, 1427, 1386, 1348, 1282, 1260, 1231 cm^{-1}.

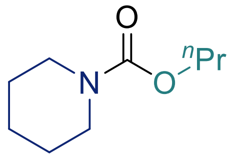

Propyl piperidine-1-carboxylate (**35**)

Yellow oil (**35**) was obtained by general procedure. Spectral data correlated with that previously reported in the literature.

^{1}H NMR (400 MHz, CDCl$_3$): δ 4.03 (t, J = 6.0, 2H), 3.41 (m, 4H), 1.69-1.52 (m, 8H), 0.94 (t, J=6.0, 3H). **^{13}C NMR (101 MHz, CDCl$_3$)**: δ 155.7, 66.7, 44.7, 25.7, 24.4, 22.4, 10.4. **IR (ATR)**: 2934, 2854, 1694, 1468, 1426, 1350, 1282, 1260, 1230 cm^{-1}.

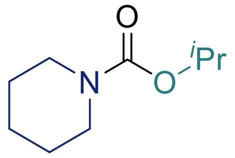

Isopropyl piperidine-1-carboxylate (**36**)

Yellow oil (**36**) was obtained by general procedure. Spectral data correlated with that previously reported in the literature.

^{1}H NMR (400 MHz, CDCl$_3$): δ 4.91 (quin, J = 6.0 Hz, 1H), 3.39 (t, J = 6.0 Hz, 4H), 1.59-1.51 (m, 6H), 1.23 (d, J = 4.0 Hz, 6H). **^{13}C NMR (101 MHz, CDCl$_3$)**: δ 155.3, 68.2, 44.6, 25.7, 24.4, 22.3. **IR (ATR)**: 2978, 2934, 2854, 1688, 1470, 1424, 1374, 1346, 1282, 1262, 1232 cm^{-1}.

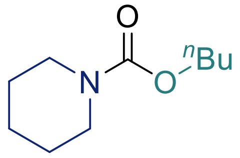

Butyl piperidine-1-carboxylate (**37**)

Yellow oil (**37**) was obtained by general procedure. Spectral data correlated with that previously reported in the literature.

^{1}H NMR (400 MHz, CDCl$_3$): δ 4.07 (t, J = 6.0 Hz, 2H), 3.41(t, J = 6.0 Hz, 4H), 1.65-1.52 (m, 8H), 1.42-1.34 (m, 2H), 0.94 (t, J = 8.0 Hz, 3H). **^{13}C NMR (101 MHz, CDCl$_3$)**: δ 155.7, 65.0, 44.7, 31.1, 25.7, 24.4, 19.2, 13.8. **IR (ATR)**: 2934, 2856, 1694, 1468, 1426, 1350, 1260, 1230 cm^{-1}.

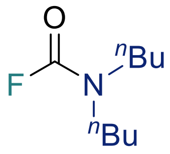

N,N-Dibuthylcarbamoyl fluoride (40)

Yellow oil (**40**) was obtained by general procedure.

^{1}H NMR (400 MHz, $CDCl_3$): δ 3.26-3.17 (m, 4H), 1.62-1.51 (m, 4H), 1.38-1.28 (m, 4H), 0.96-0.92 (m, 6H). **^{13}C NMR (101 MHz, $CDCl_3$)**: δ 149.2, 146.3, 48.2, 47.5, 30.7, 29.5, 19.8, 19.8, 13.7, 13.7. **IR (ATR)**: 2960, 2934, 2874, 1780, 1467, 1421, 1380, 1372, 1255, 1217 cm^{-1}.

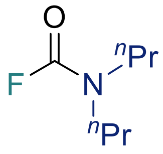

N,N-Dipropylcarbamoyl fluoride (41)

Yellow oil (**41**) was obtained by general procedure.

^{1}H NMR (400 MHz, $CDCl_3$): δ 3.23-3.15 (m, 4H), 1.68-1.56 (m, 4H), 0.95-0.89 (m, 6H). **^{13}C NMR (101 MHz, $CDCl_3$)**: δ 149.3, 146.4, 50.1, 49.4, 21.8, 20.7, 11.0, 11.0. **IR (ATR)**: 2965, 2935, 2879, 1780, 1465, 1419, 1384, 1346, 1303, 1237 cm^{-1}.

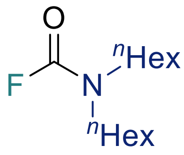

N,N-Dihexylcarbamoyl fluoride (42)

Yellow oil (**42**) was obtained by general procedure.

^{1}H NMR (400 MHz, $CDCl_3$): δ 3.25-3.16 (m, 4H), 1.62-1.52 (b, 4H), 1.34-1.27 (b, 12H), 0.91-0.88 (b, 6H). **^{13}C NMR (101 MHz, $CDCl_3$)**: δ 149.2, 146.3, 48.5, 47.8, 31.5, 31.4, 28.6, 27.4, 26.3, 26.2, 22.5, 22.5, 14.0, 14.0. **IR (ATR)**: 2955, 2927, 2858, 1783, 1460, 1420, 1379, 1297, 1256, 1224 cm^{-1}.

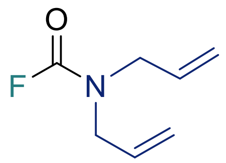

***N,N*-Diallylcarbamoyl fluoride (43)**

Yellow oil (**43**) was obtained by general procedure.

^{1}H NMR (400 MHz, $CDCl_3$): δ 5.85-5.72 (m, 2H), 5.28-5.18 (m, 4H), 3.91-3.82 (m, 4H). **^{13}C NMR (101 MHz, $CDCl_3$)**: δ 148.9, 146.0, 131.8, 131.5, 118.9, 118.2, 50.1, 49.1. **IR (ATR)**: 3083, 2925, 2854, 1785, 1726, 1645, 1459, 1408, 1229 cm^{-1}.

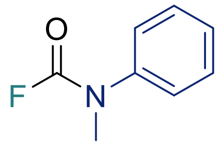

***N,N*-Methyl(phenyl)carbamoyl fluoride (44)**

Yellow oil (**44**) was obtained by general procedure.

^{1}H NMR (400 MHz, $CDCl_3$): δ 7.43-7.41 (m, 2H), 7.39-7.30 (m, 2H), 7.24-7.22 (m, 1H), 3.38-3.36 (d, 3H). **^{13}C NMR (101 MHz, $CDCl_3$)**: δ 140.75, 140.21, 131.0, 129.4, 129.1, 127.7, 127.1, 125.6, 124.6, 122.8, 38.9, 37.9. **IR (ATR)**: 3060, 2924, 2854, 1790, 1598, 1497, 1370, 1290, 1272 cm^{-1}.

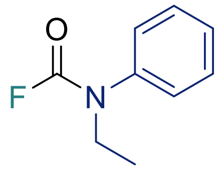

***N,N*-Ethyl(phenyl)carbamoyl fluoride (44)**

Yellow oil (**45**) was obtained by general procedure.

^{1}H NMR (400 MHz, $CDCl_3$): δ 7.43-7.32 (m, 3H), 7.21-7.19 (m, 2H), 3.76-3.71 (m, 2H), 1.26-1.20 (m, 3H). **^{13}C NMR (101 MHz, $CDCl_3$)**: δ 147.5, 144.7, 139.1, 131.9, 129.4, 128.9, 128.0, 127.7, 126.9, 126.2, 46.8, 46.8, 14.2, 12.8. **IR (ATR)**: 2975, 2937, 1783, 1594, 1493, 1395, 1272 cm^{-1}.

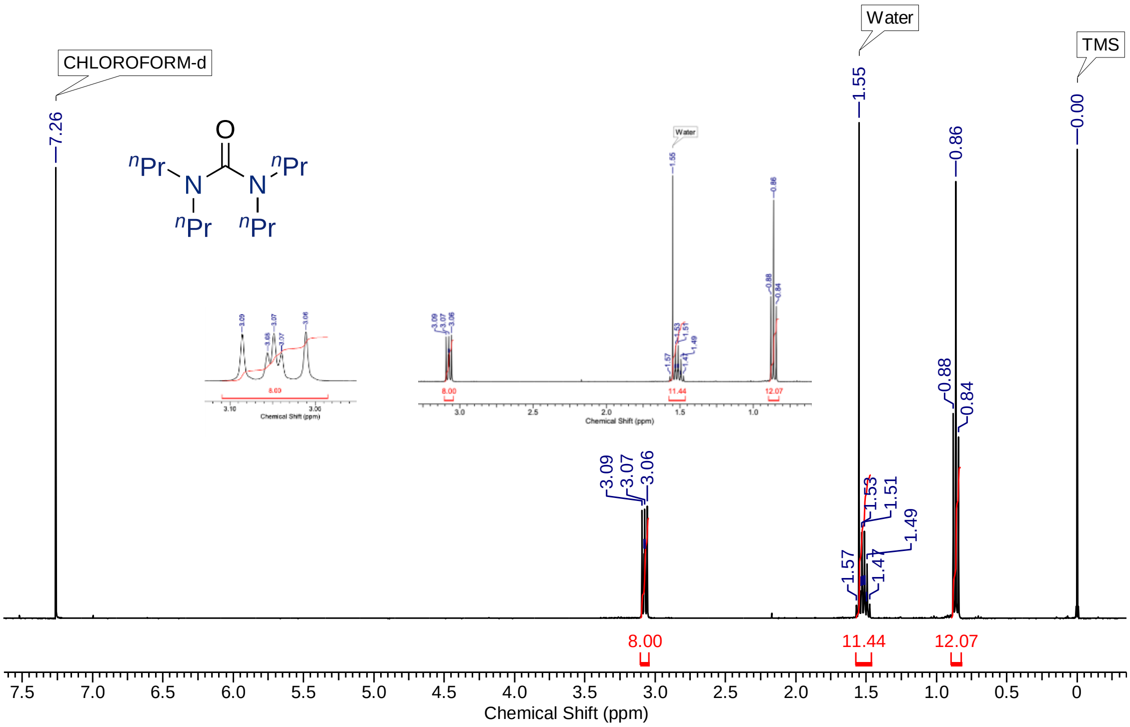

Figure S6-1. ^{1}H NMR spectrum of **4** in $CDCl_3$ (400 MHz).

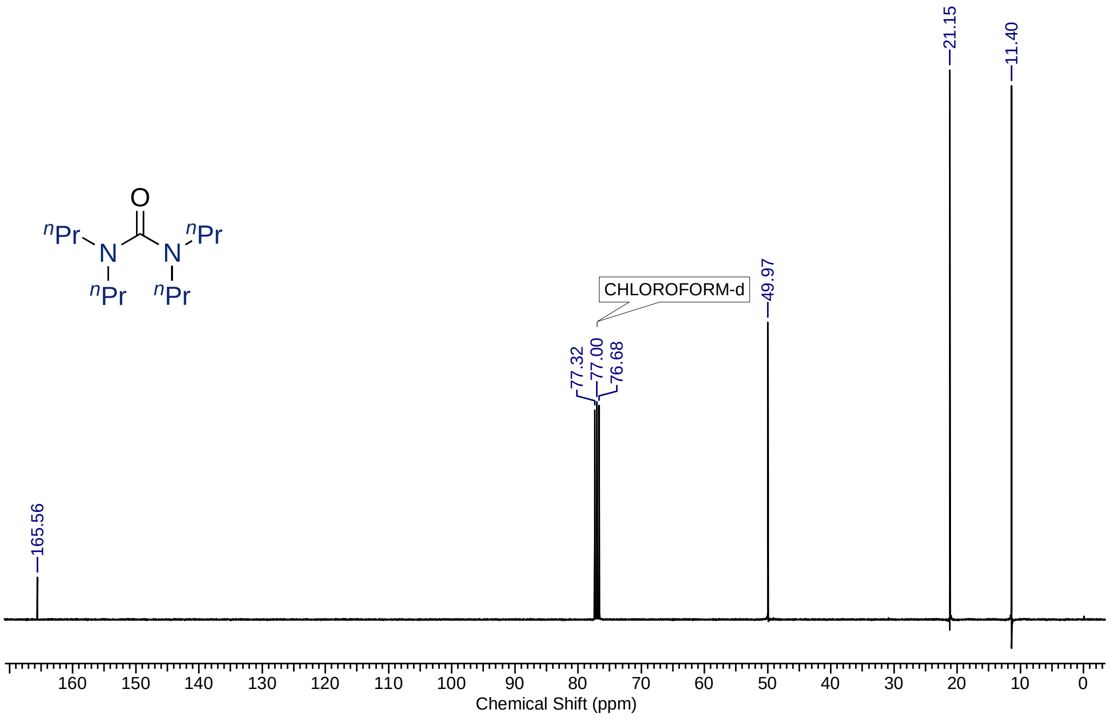

Figure S6-2. $^{13}C\{^1H\}$ NMR spectrum of **4** in $CDCl_3$ (101 MHz).

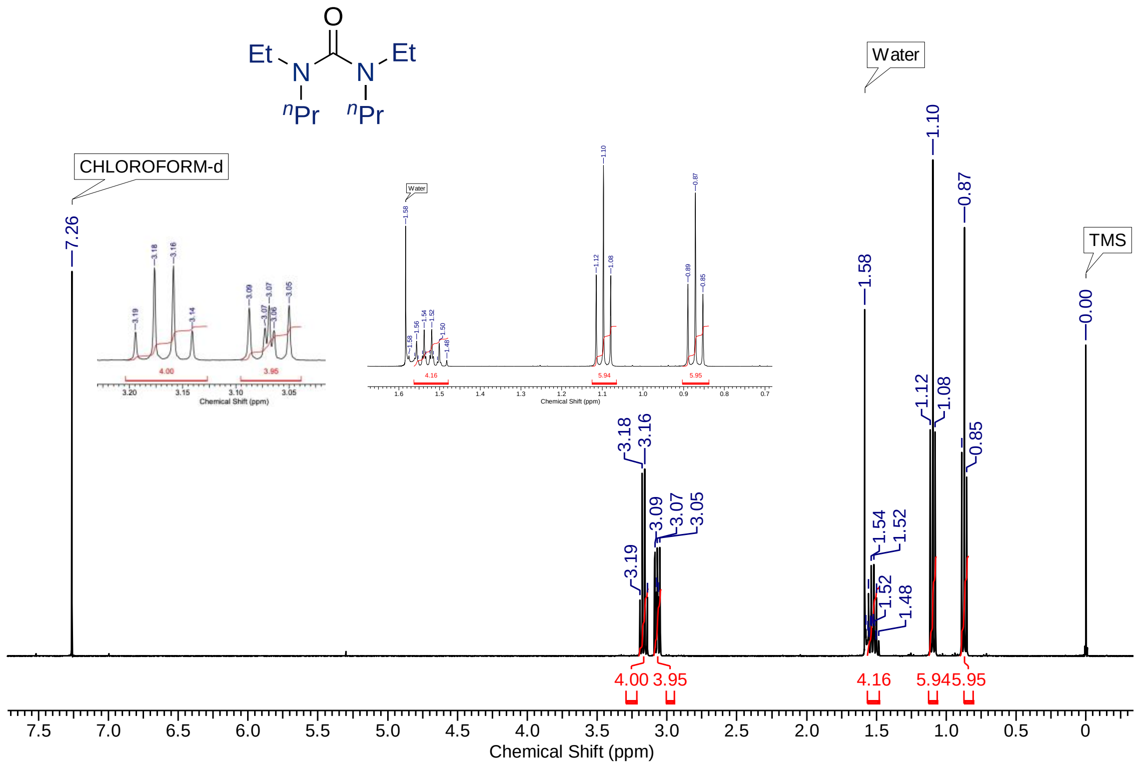

Figure S6-3. ^{1}H NMR spectrum of **6** in $CDCl_3$ (400 MHz).

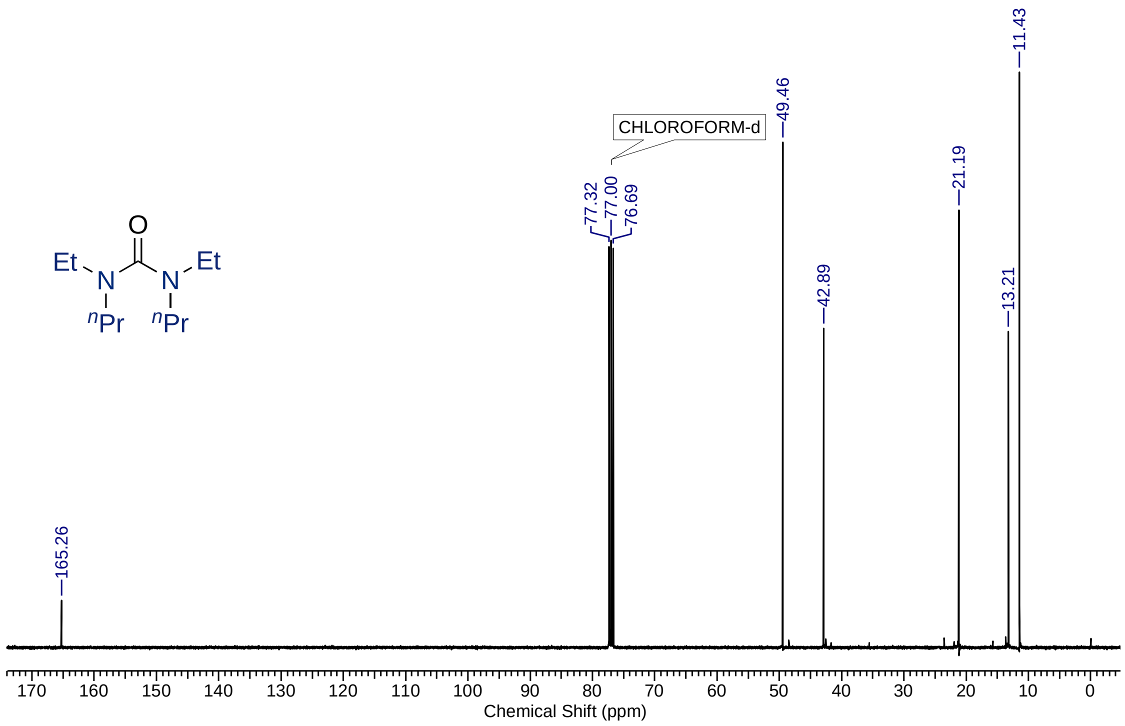

Figure S6-4. $^{13}C\{^{1}H\}$ NMR spectrum of **6** in $CDCl_3$ (101 MHz).

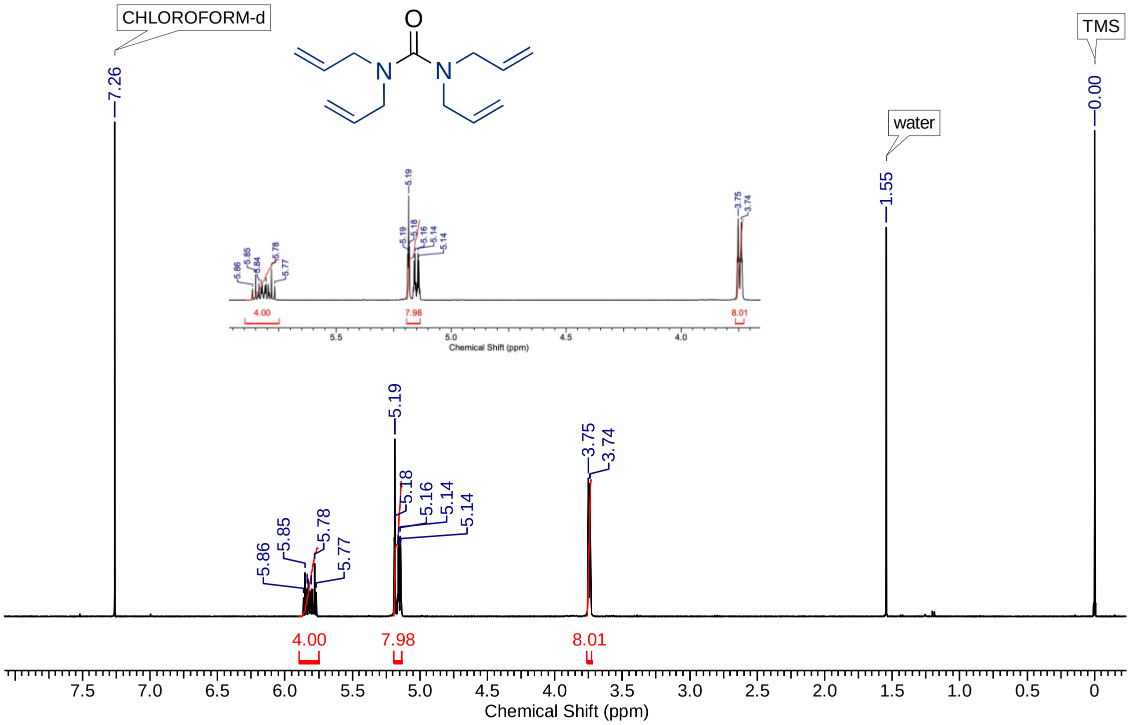

Figure S6-5. ^{1}H NMR spectrum of **7** in $CDCl_3$ (400 MHz).

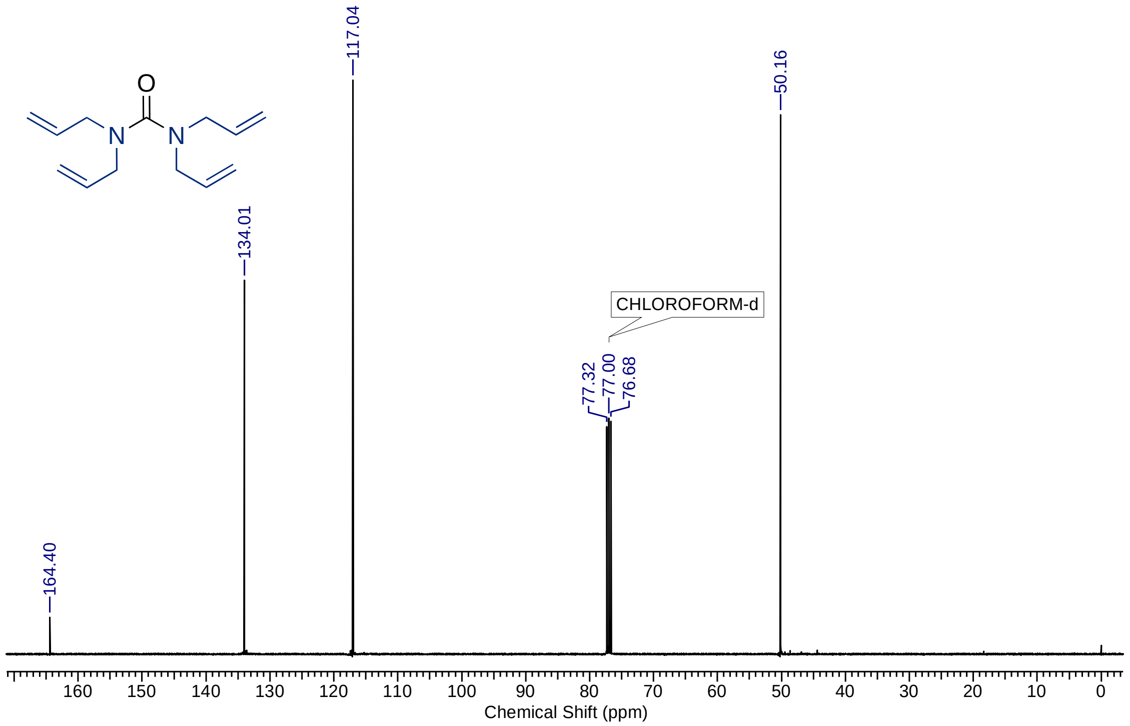

Figure S6-6. $^{13}C\{^{1}H\}$ NMR spectrum of **7** in $CDCl_3$ (101 MHz).

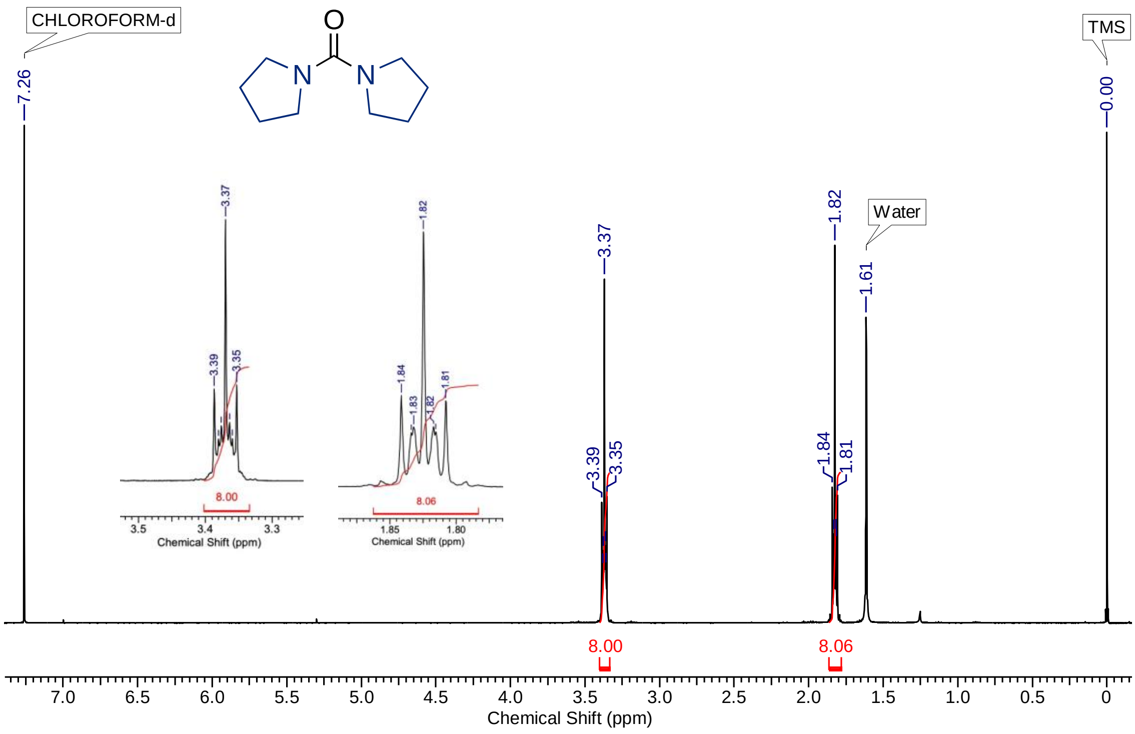

Figure S6-7. ^{1}H NMR spectrum of **9** in $CDCl_3$ (400 MHz).

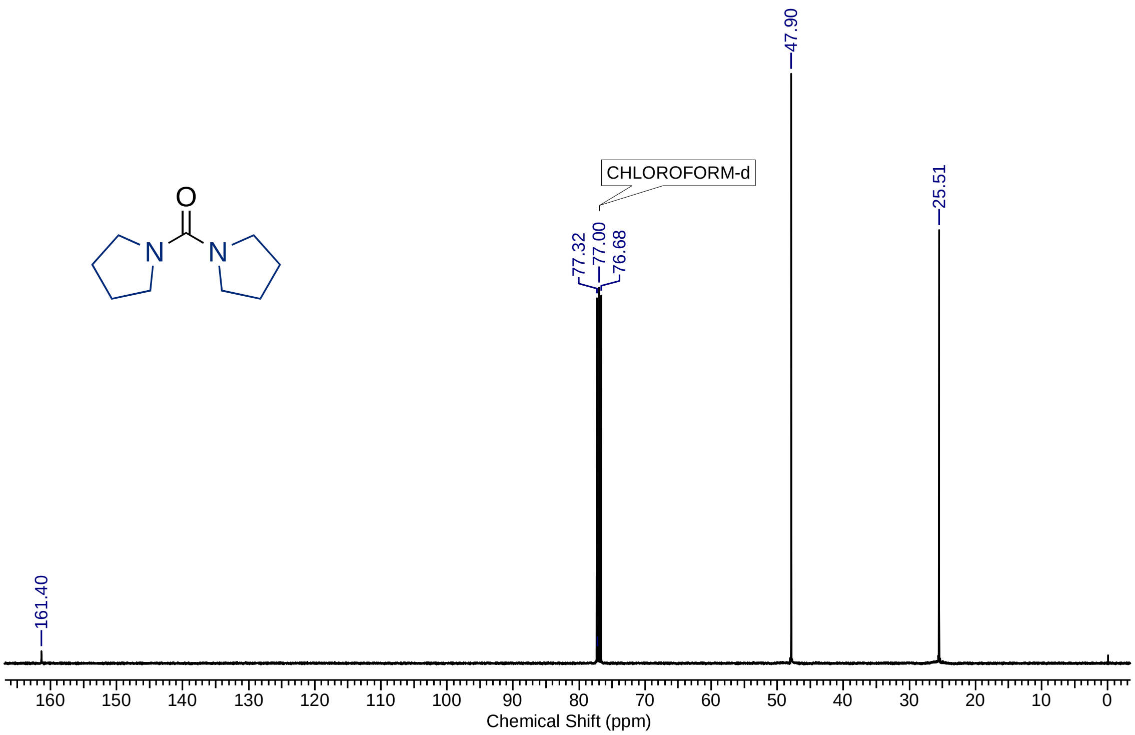

Figure S6-8. $^{13}C\{^{1}H\}$ NMR spectrum of **9** in $CDCl_3$ (101 MHz).

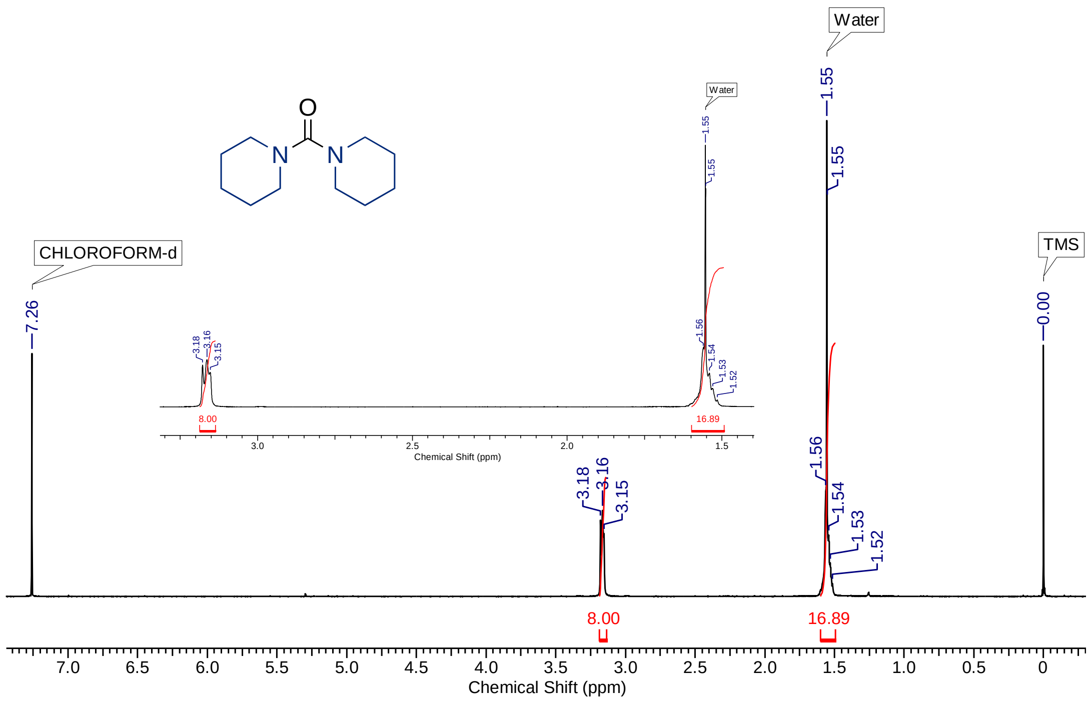

Figure S6-9.. ^{1}H NMR spectrum of **10** in $CDCl_3$ (400 MHz).

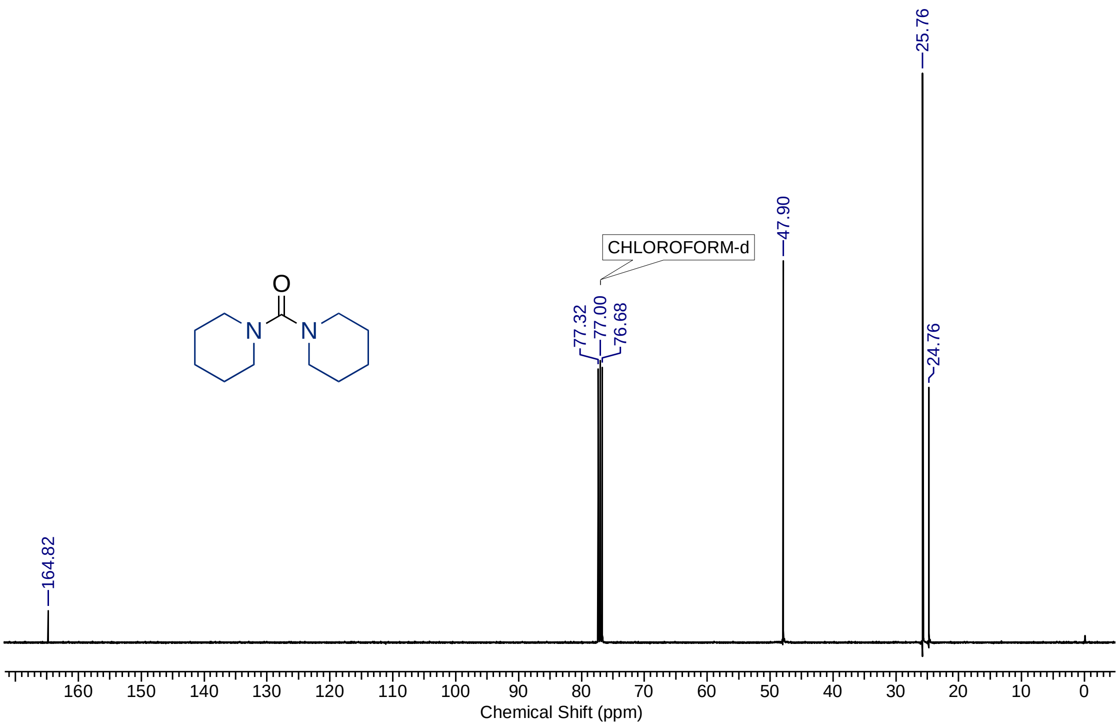

Figure S6-11. $^{13}C\{^{1}H\}$ NMR spectrum of **10** in $CDCl_3$ (101 MHz).

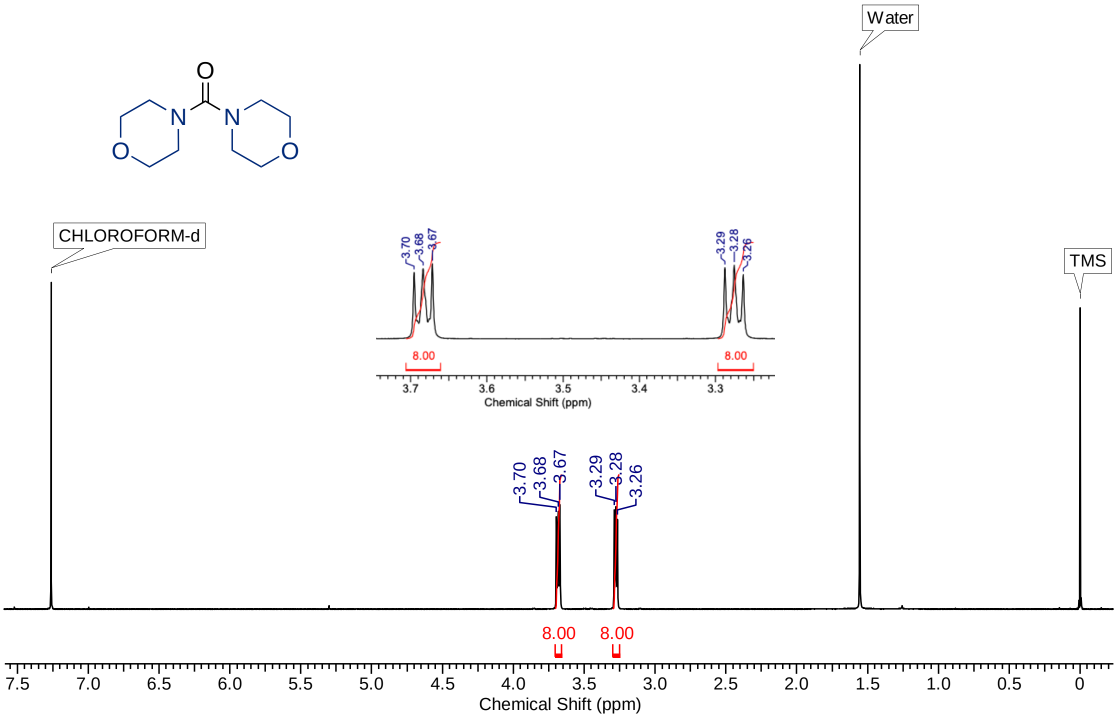

Figure S6-12. ^{1}H NMR spectrum of **11** in $CDCl_3$ (400 MHz).

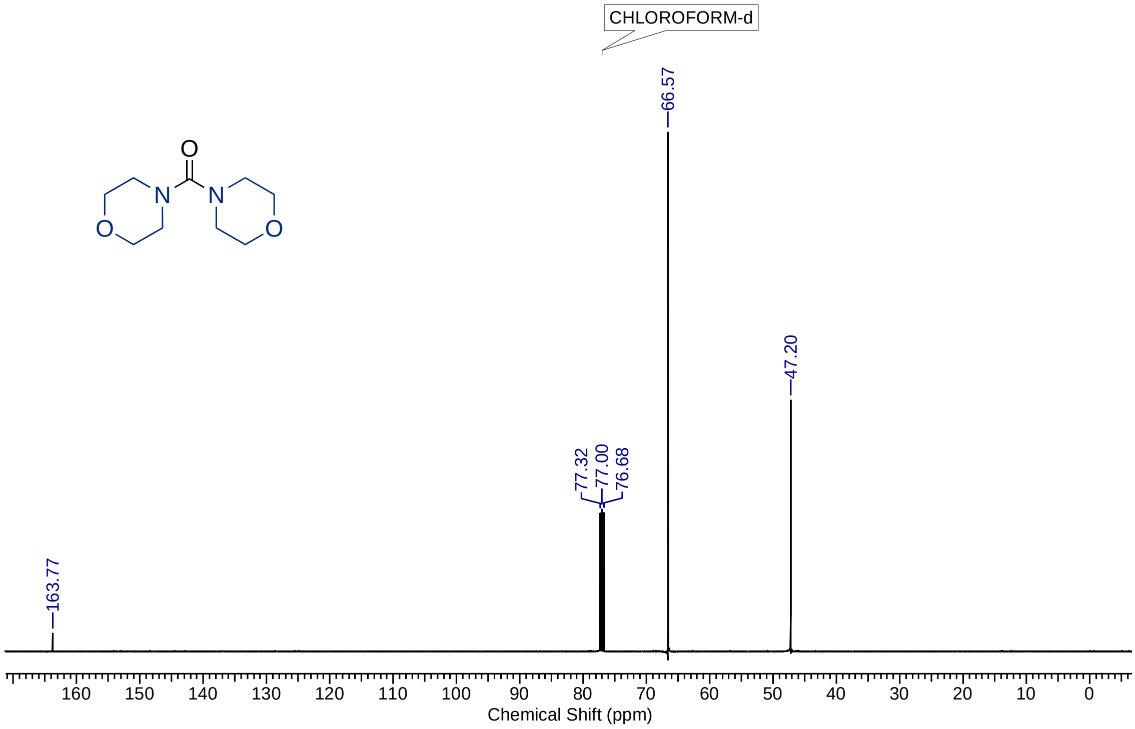

Figure S6-13. $^{13}C\{^{1}H\}$ NMR spectrum of **11** in $CDCl_3$ (101 MHz).

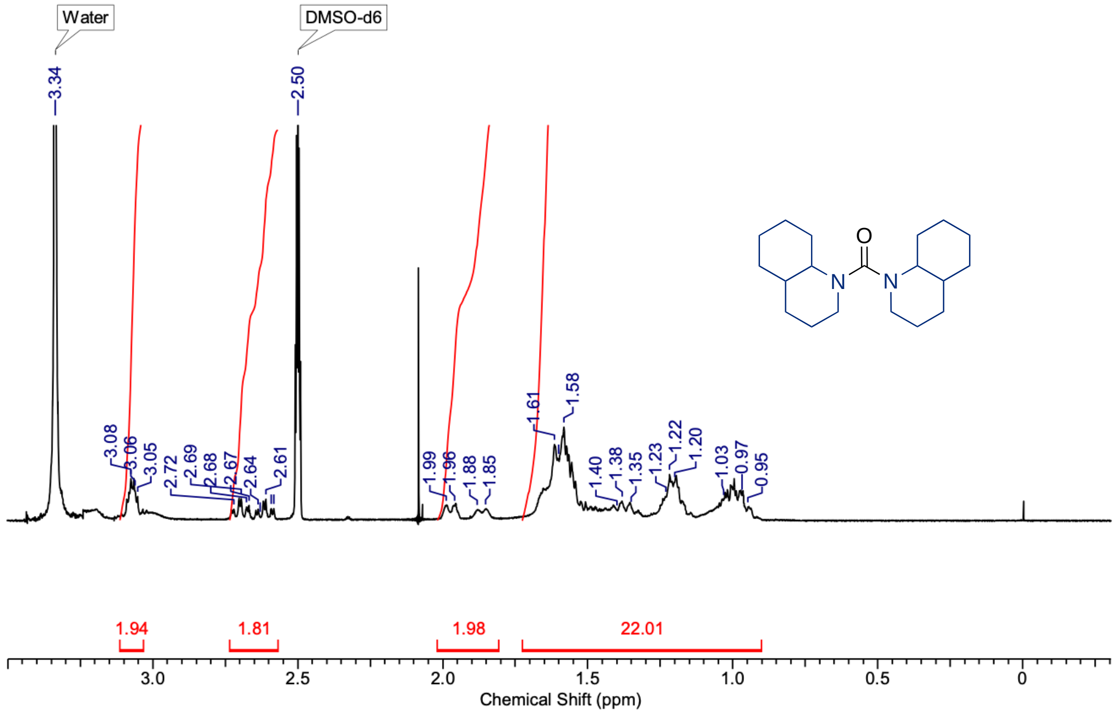

Figure S6-14. ^{1}H NMR spectrum of **12** in DMSO-d^{6} (400 MHz).

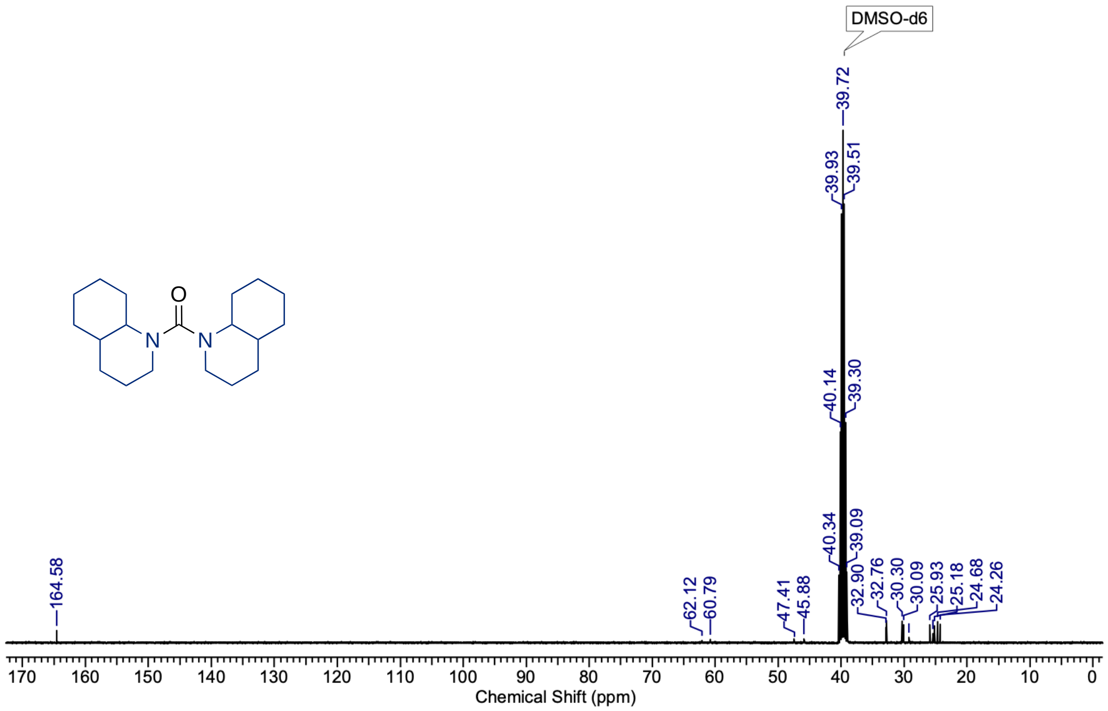

Figure S6-15. $^{13}C\{^{1}H\}$ NMR spectrum of **12** in DMSO-d^{6} (101 MHz).

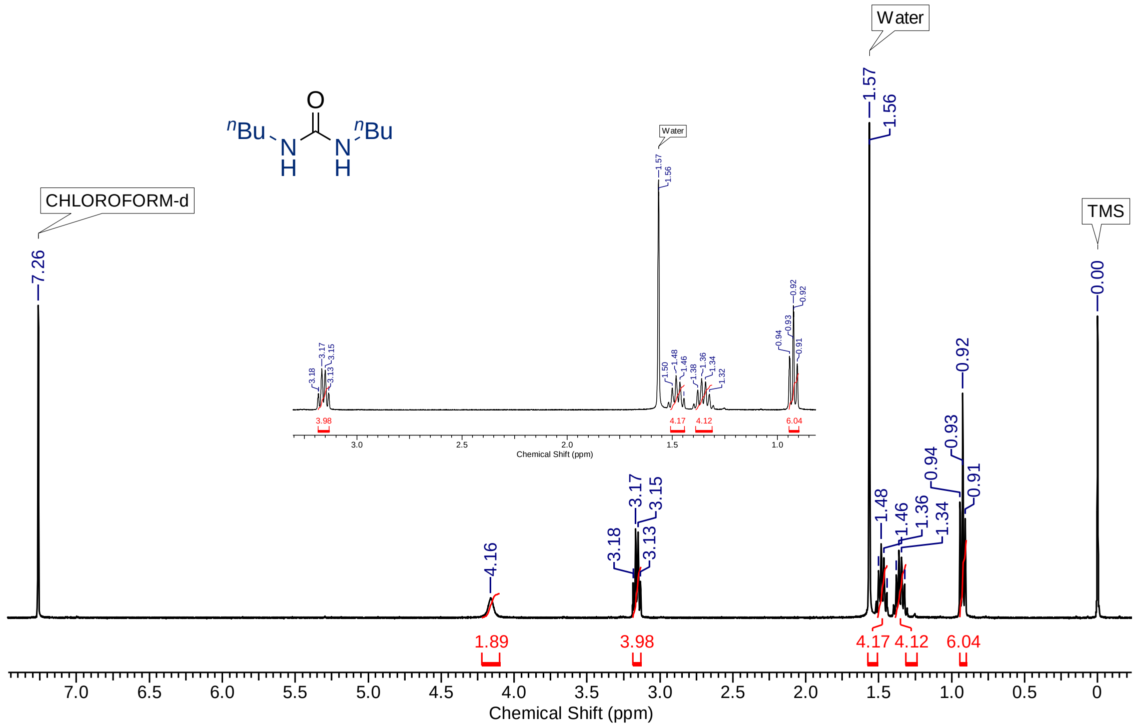

Figure S6-16. ^{1}H NMR spectrum of **13** in $CDCl_3$ (400 MHz).

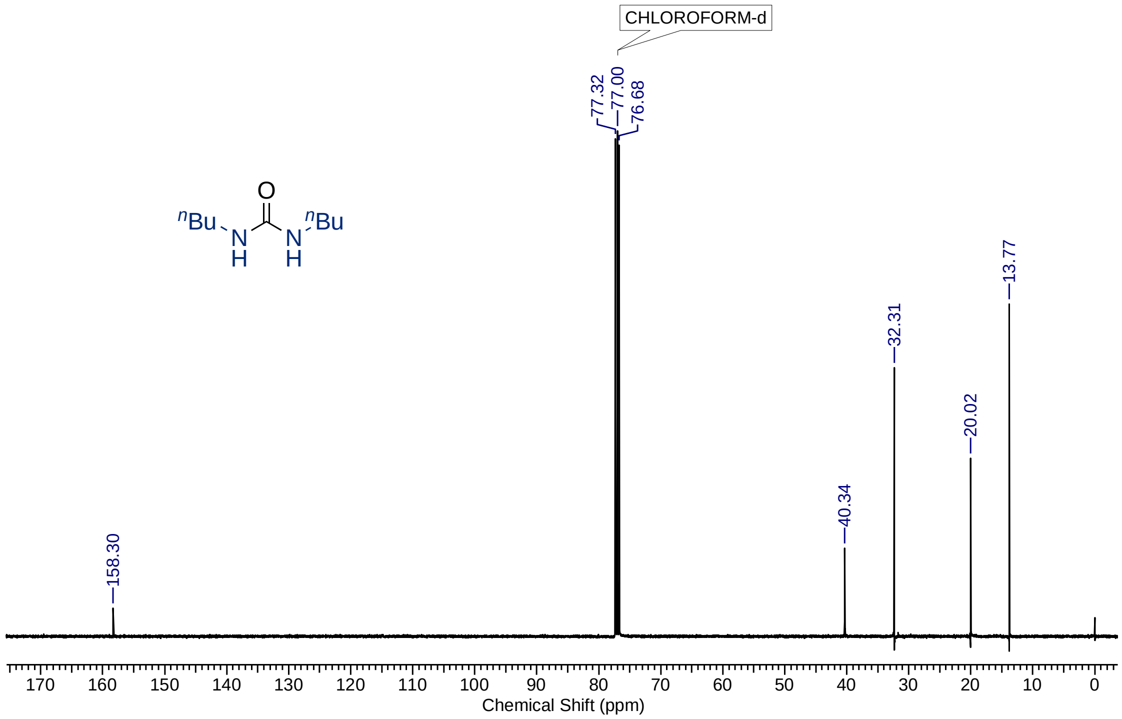

Figure S6-17. $^{13}C\{^{1}H\}$ NMR spectrum of **13** in $CDCl_3$ (101 MHz).

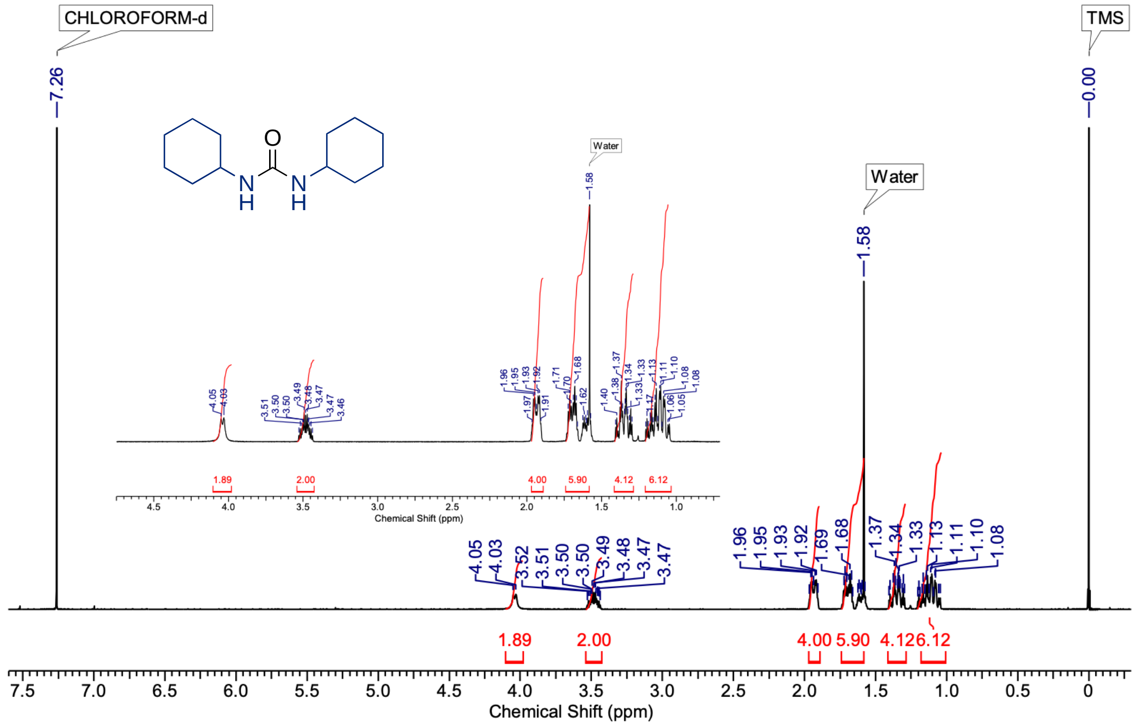

Figure S6-18. ^{1}H NMR spectrum of **14** in $CDCl_3$ (400 MHz).

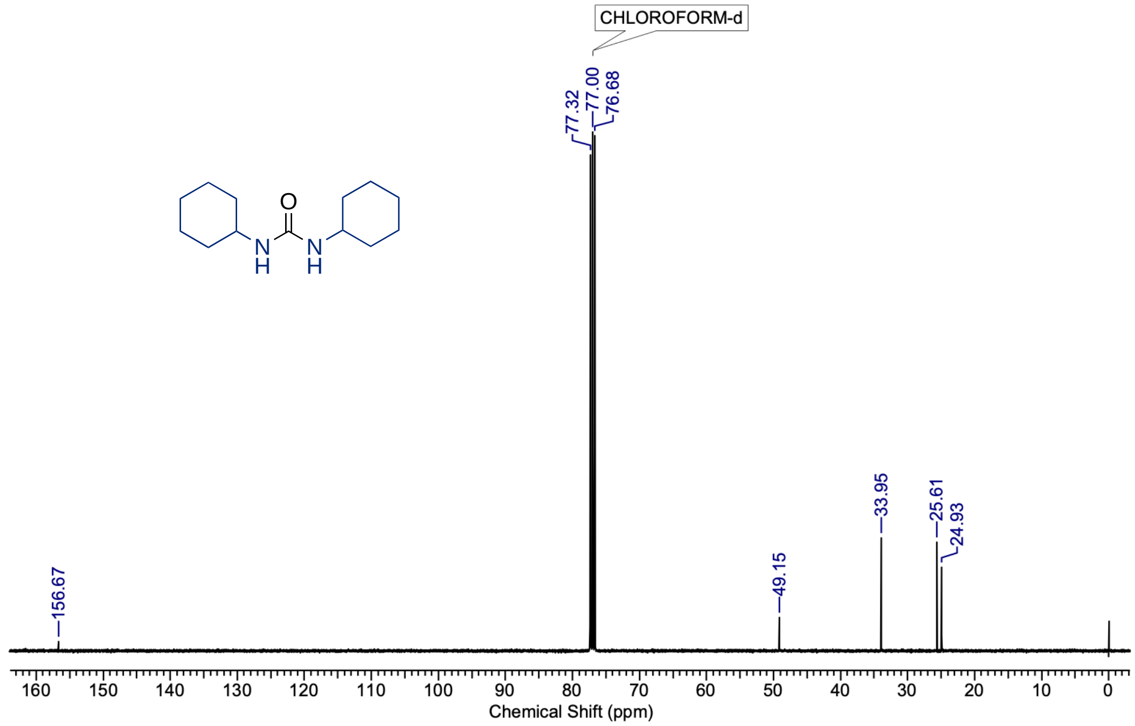

Figure S6-19. $^{13}C\{^{1}H\}$ NMR spectrum of **14** in $CDCl_3$ (101 MHz).

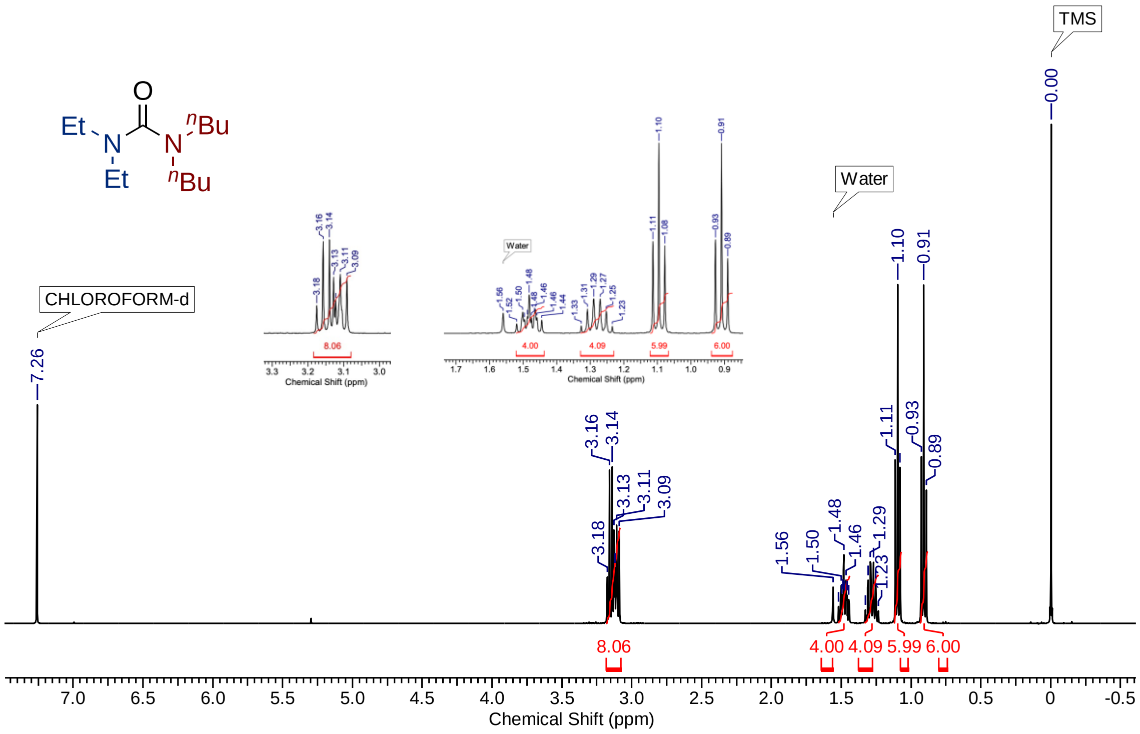

Figure S6-20. ^{1}H NMR spectrum of **17** in $CDCl_3$ (400 MHz).

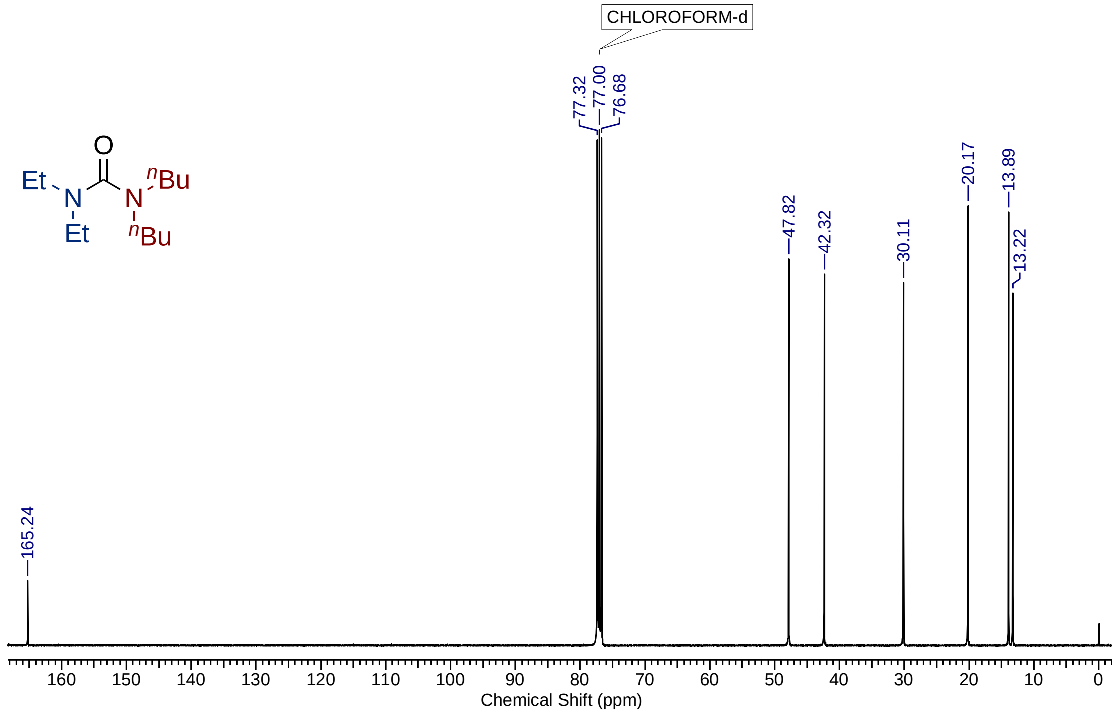

Figure S6-21. $^{13}C\{^{1}H\}$ NMR spectrum of **17** in $CDCl_3$ (101 MHz).

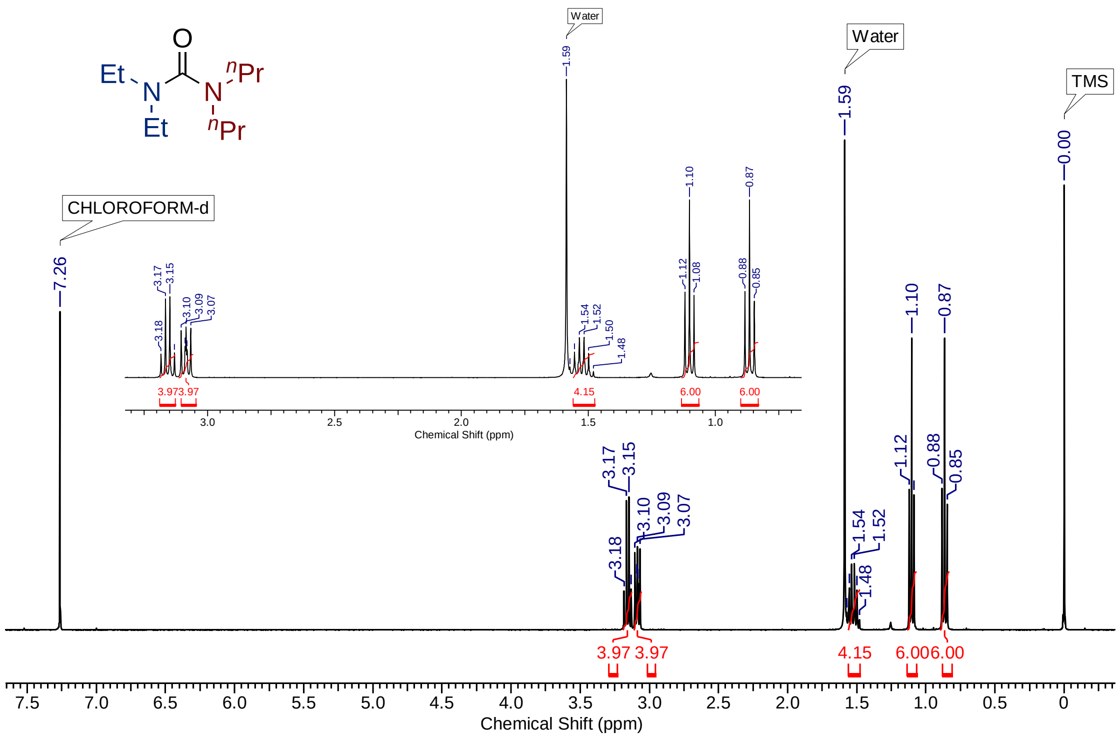

Figure S6-22. ^{1}H NMR spectrum of **18** in $CDCl_3$ (400 MHz).

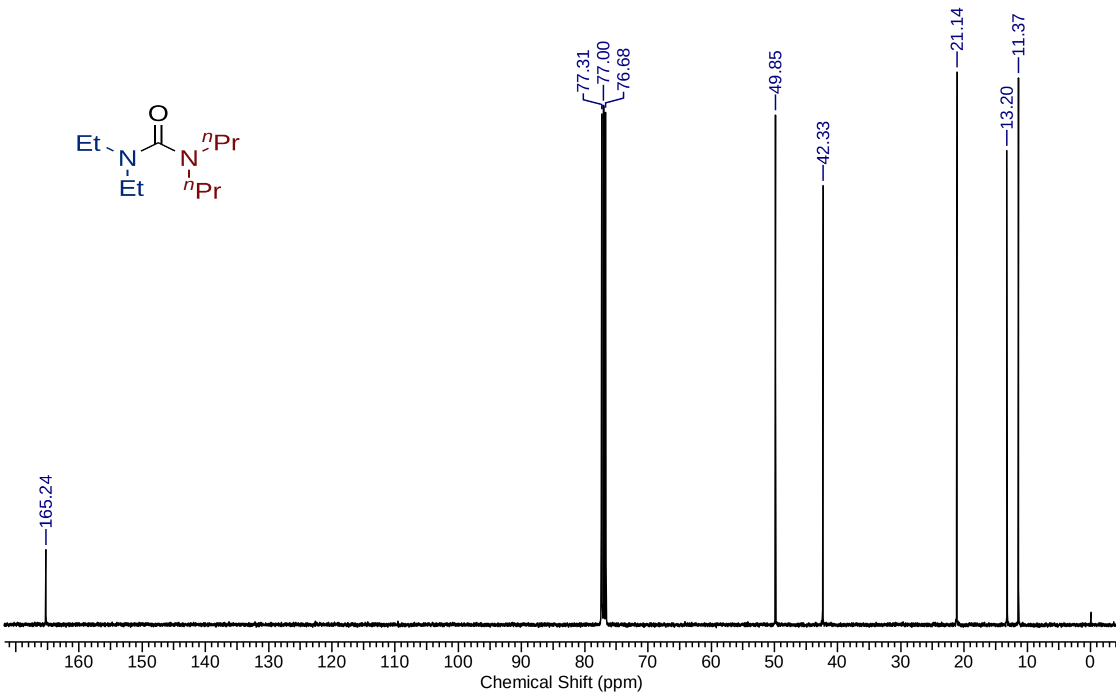

Figure S6-23. $^{13}C\{^{1}H\}$ NMR spectrum of **18** in $CDCl_3$ (101 MHz).

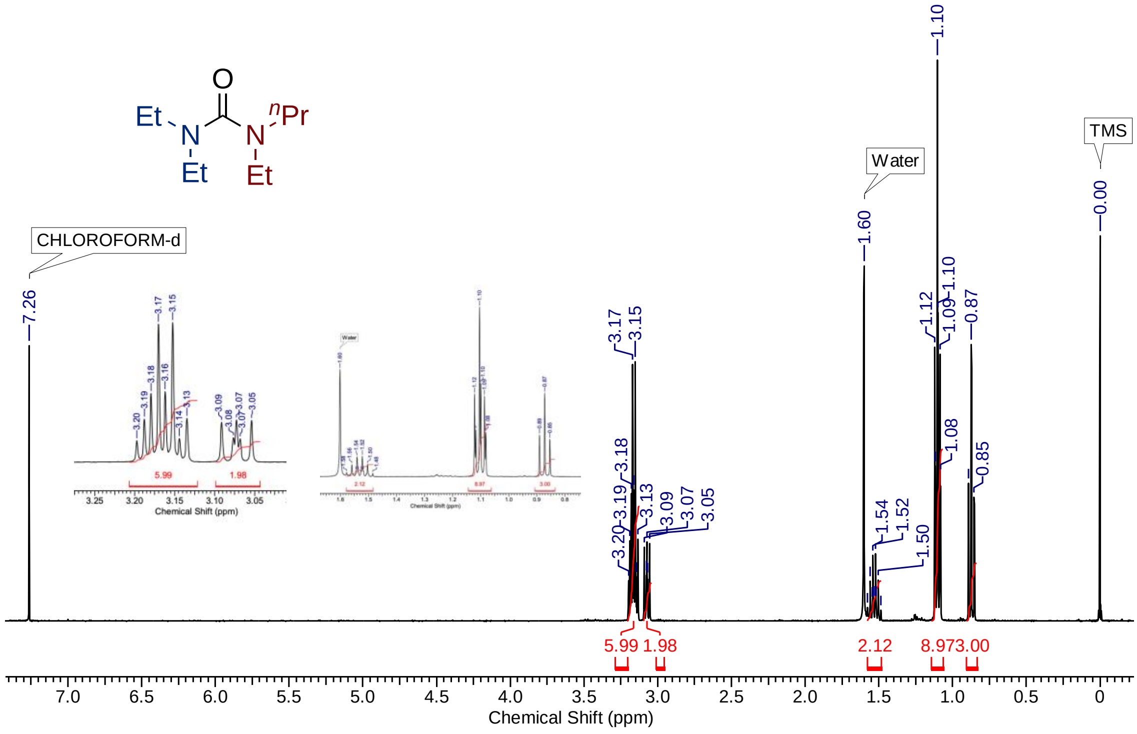

Figure S6-24. ^{1}H NMR spectrum of **19** in $CDCl_3$ (400 MHz).

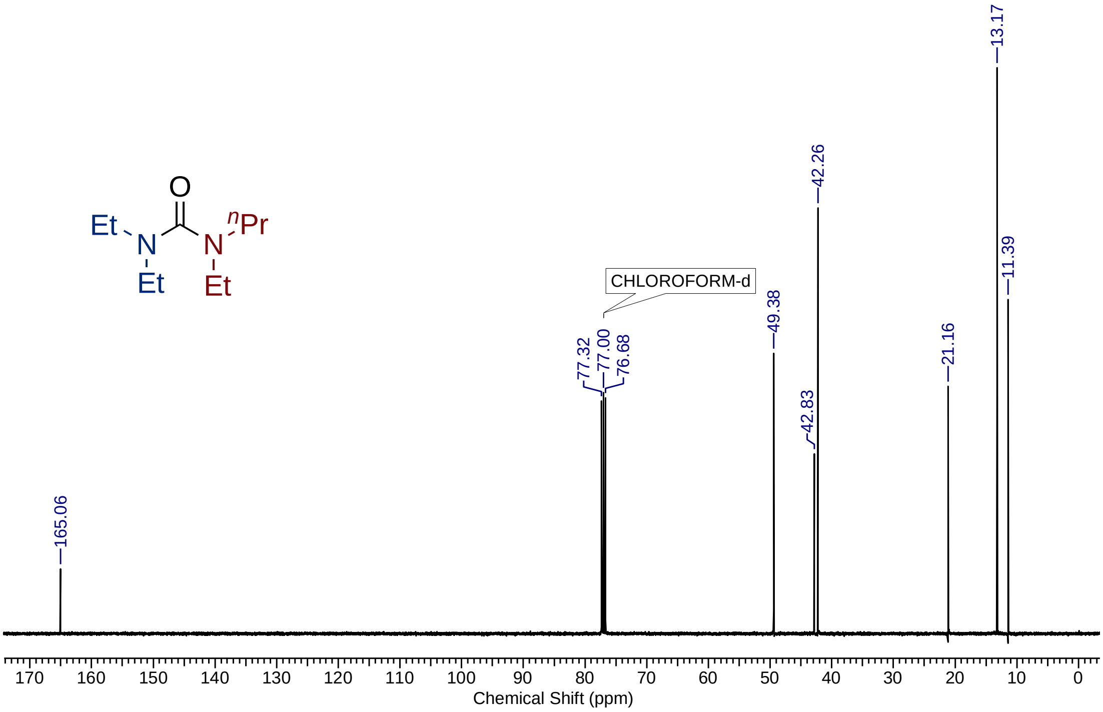

Figure S6-25. $^{13}C\{^{1}H\}$ NMR spectrum of **19** in $CDCl_3$ (101 MHz).

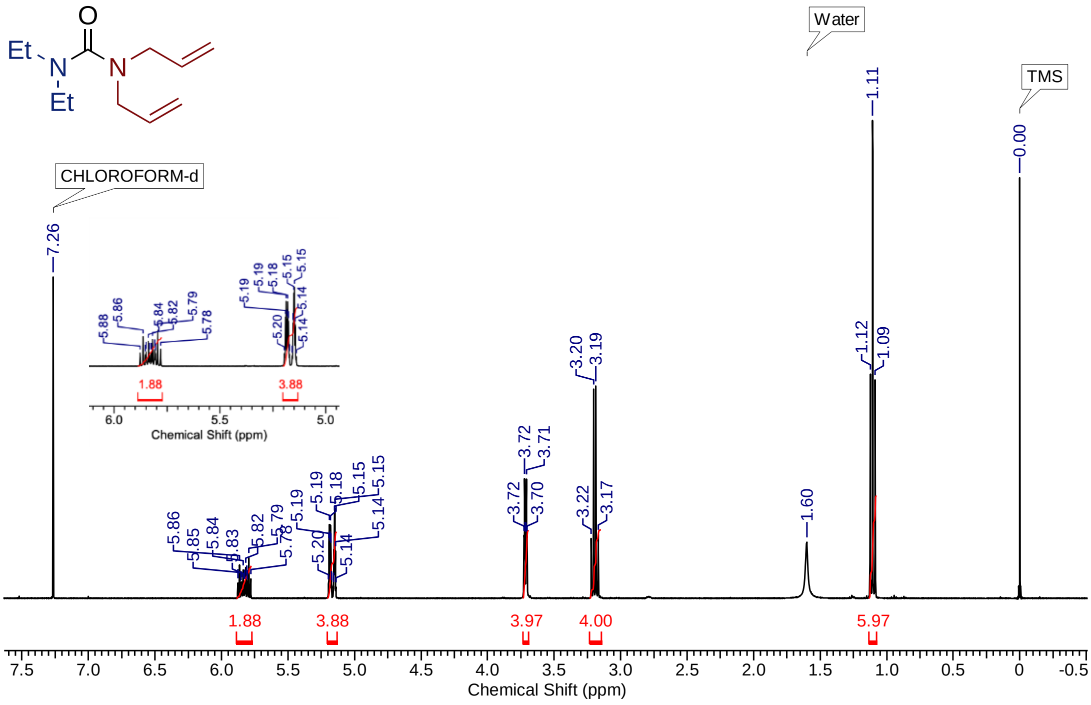

Figure S6-26. ^{1}H NMR spectrum of **20** in $CDCl_3$ (400 MHz).

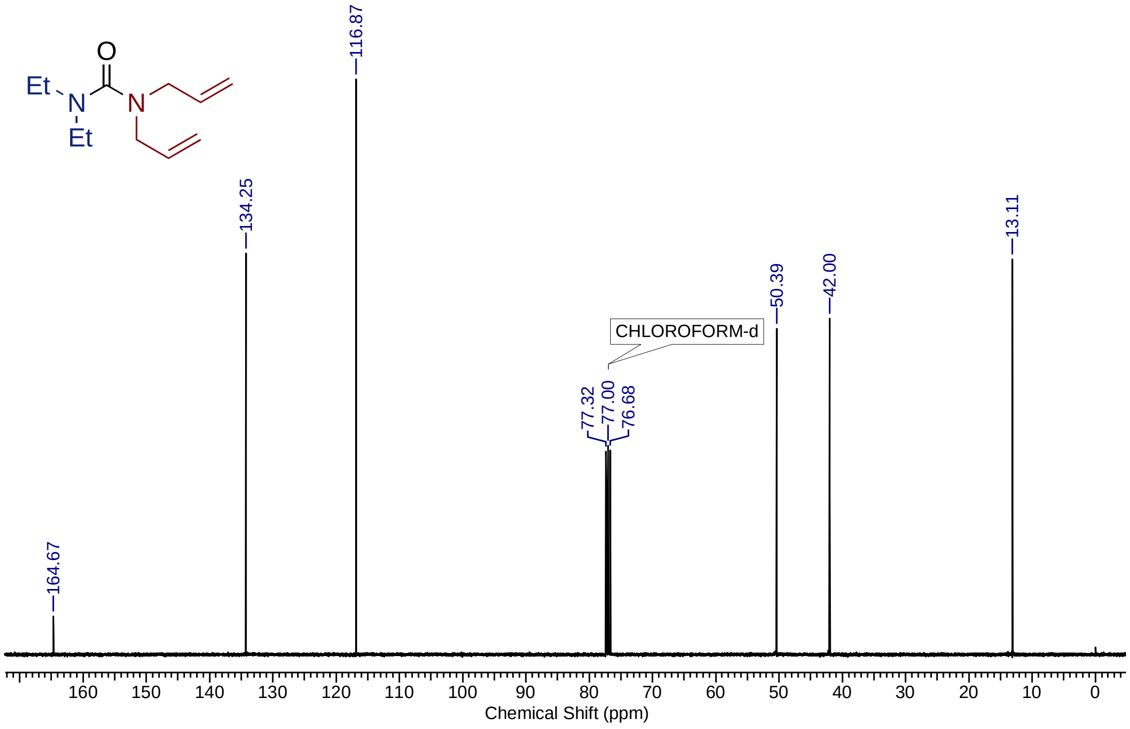

Figure S6-27. $^{13}C\{^{1}H\}$ NMR spectrum of **20** in $CDCl_3$ (101 MHz).

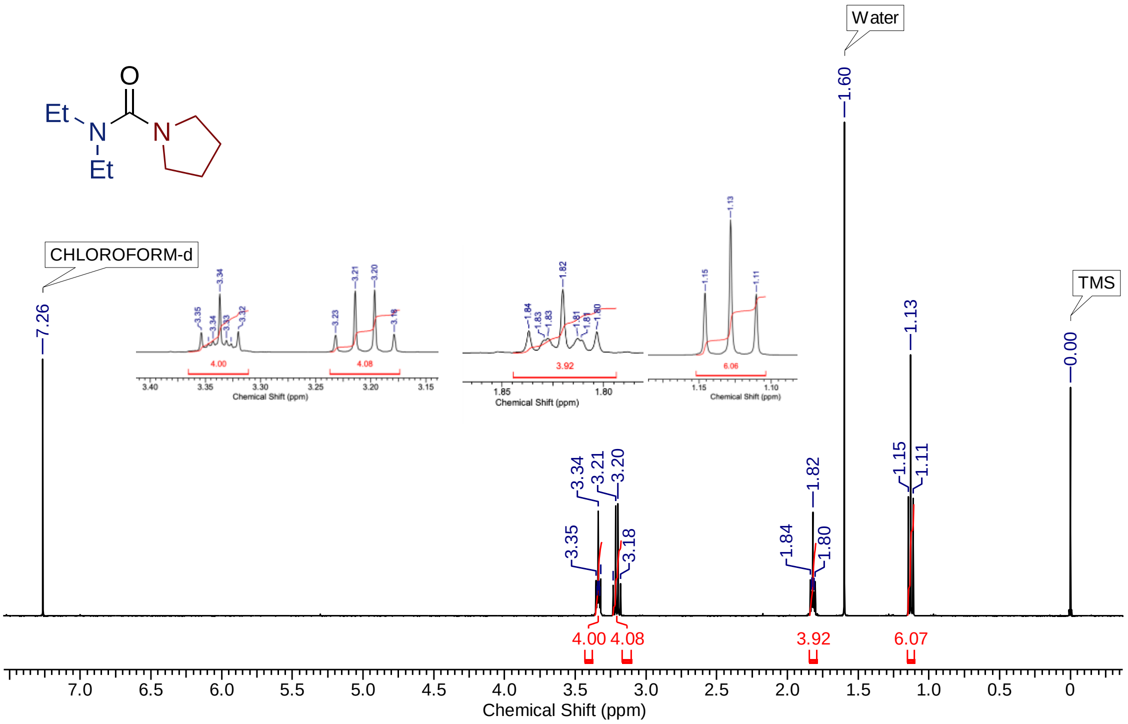

Figure S6-28. ^{1}H NMR spectrum of **21** in $CDCl_3$ (400 MHz).

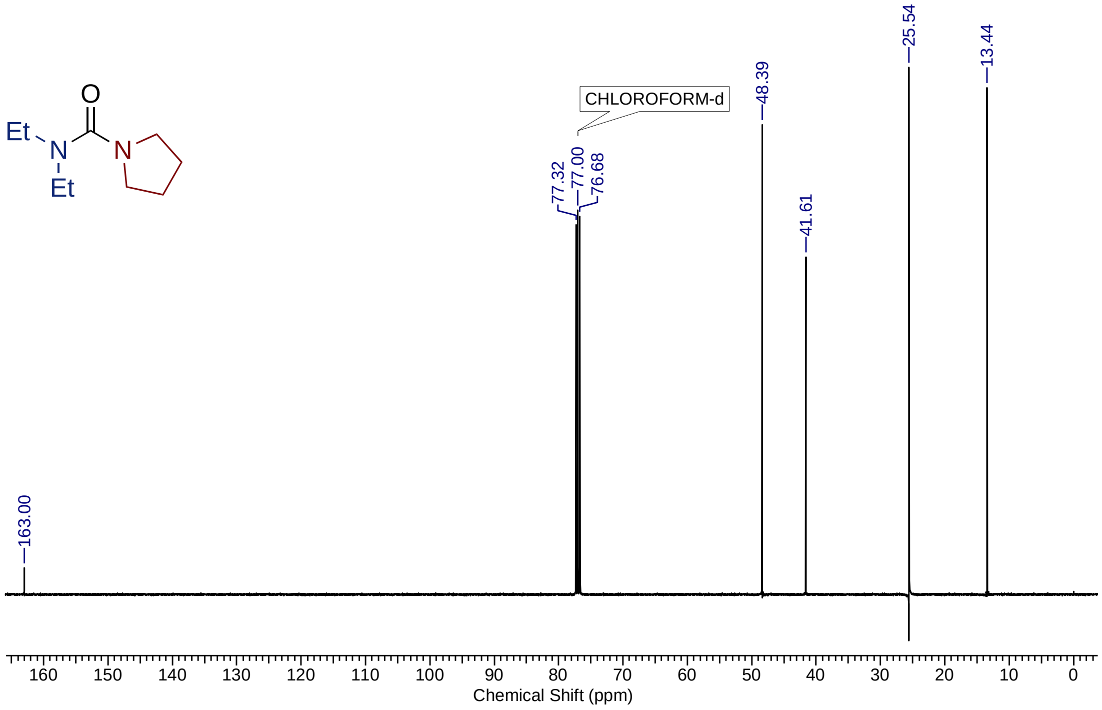

Figure S6-29. $^{13}C\{^{1}H\}$ NMR spectrum of **21** in $CDCl_3$ (101 MHz).

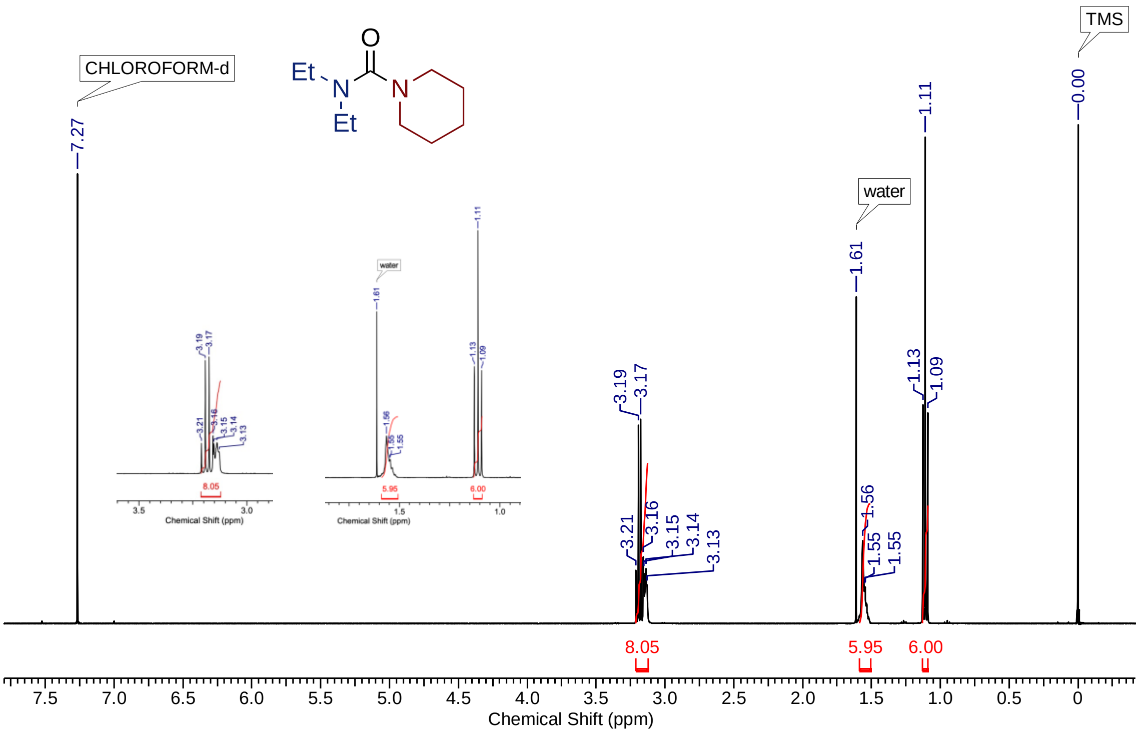

Figure S6-30. ^{1}H NMR spectrum of **22** in $CDCl_3$ (400 MHz).

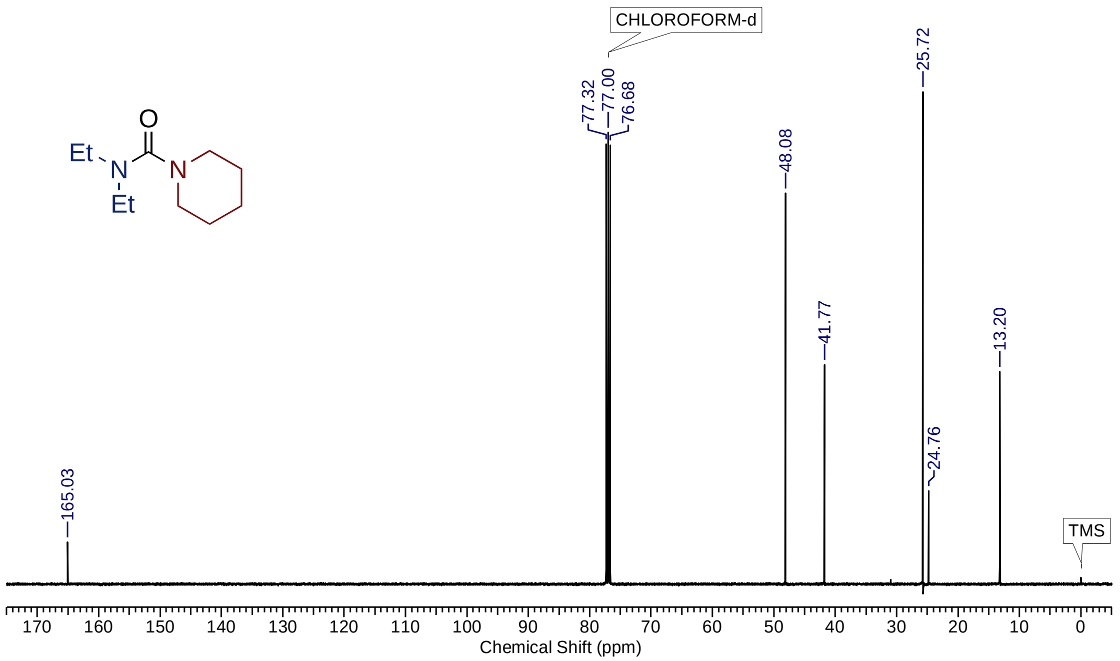

Figure S6-31. $^{13}C\{^{1}H\}$ NMR spectrum of **22** in $CDCl_3$ (101 MHz).

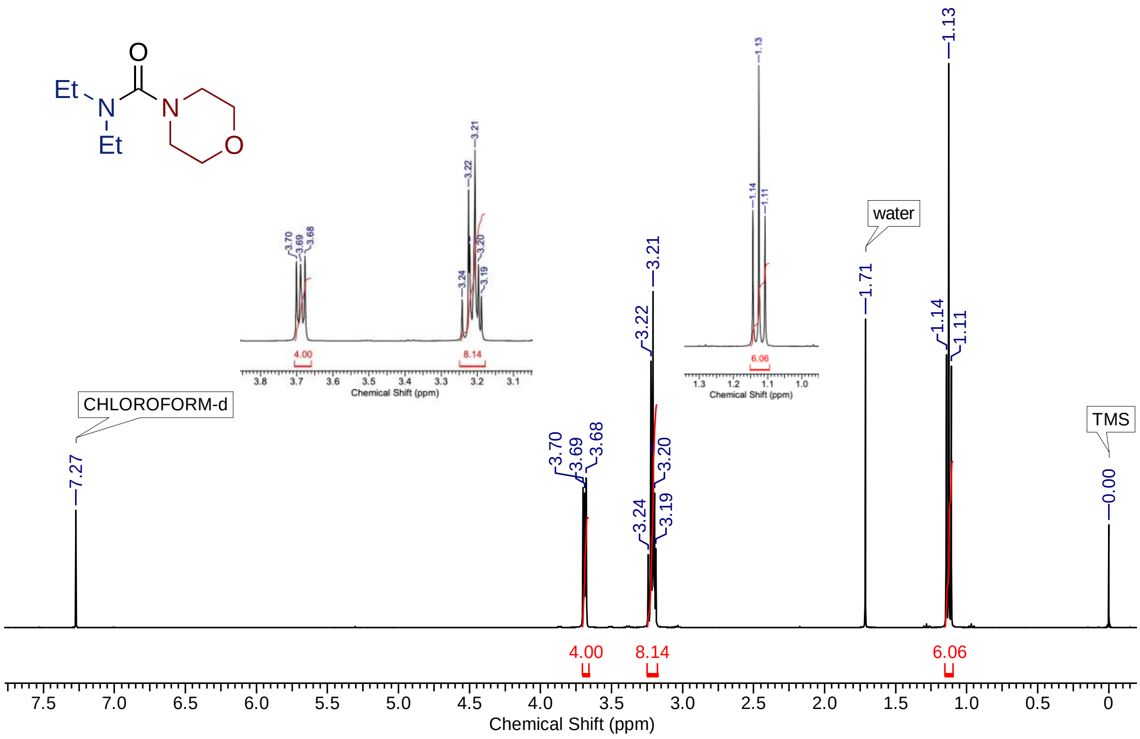

Figure S6-32. ^{1}H NMR spectrum of **23** in $CDCl_3$ (400 MHz).

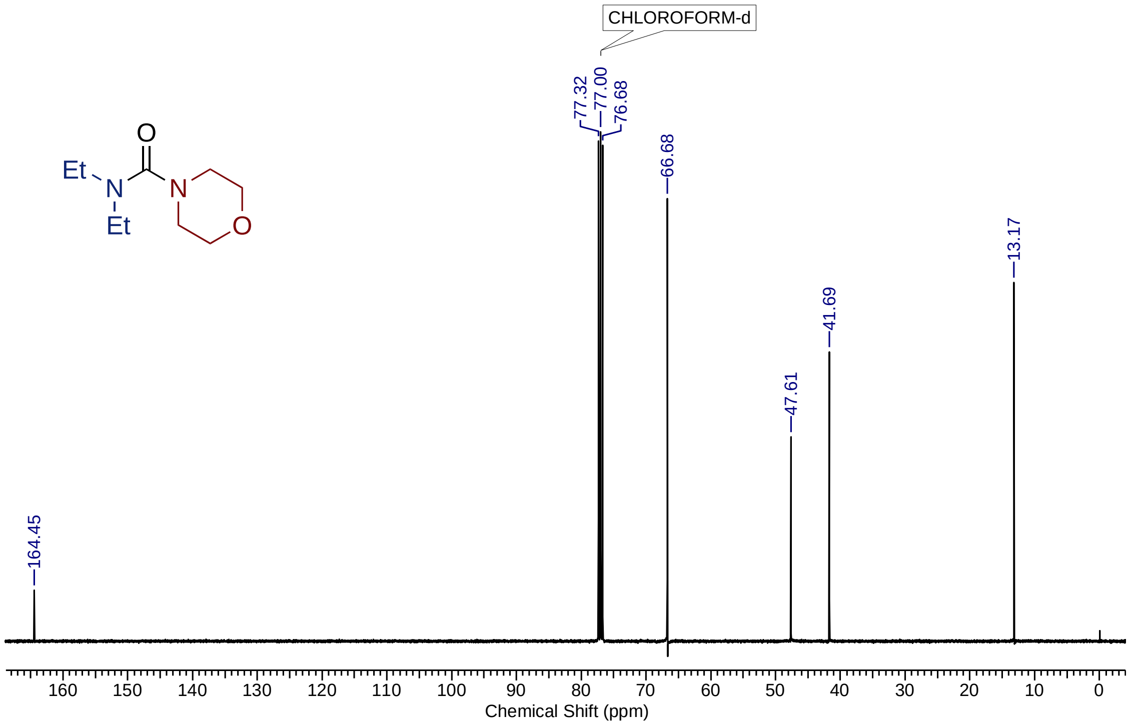

Figure S6-33. $^{13}C\{^{1}H\}$ NMR spectrum of **23** in $CDCl_3$ (101 MHz).

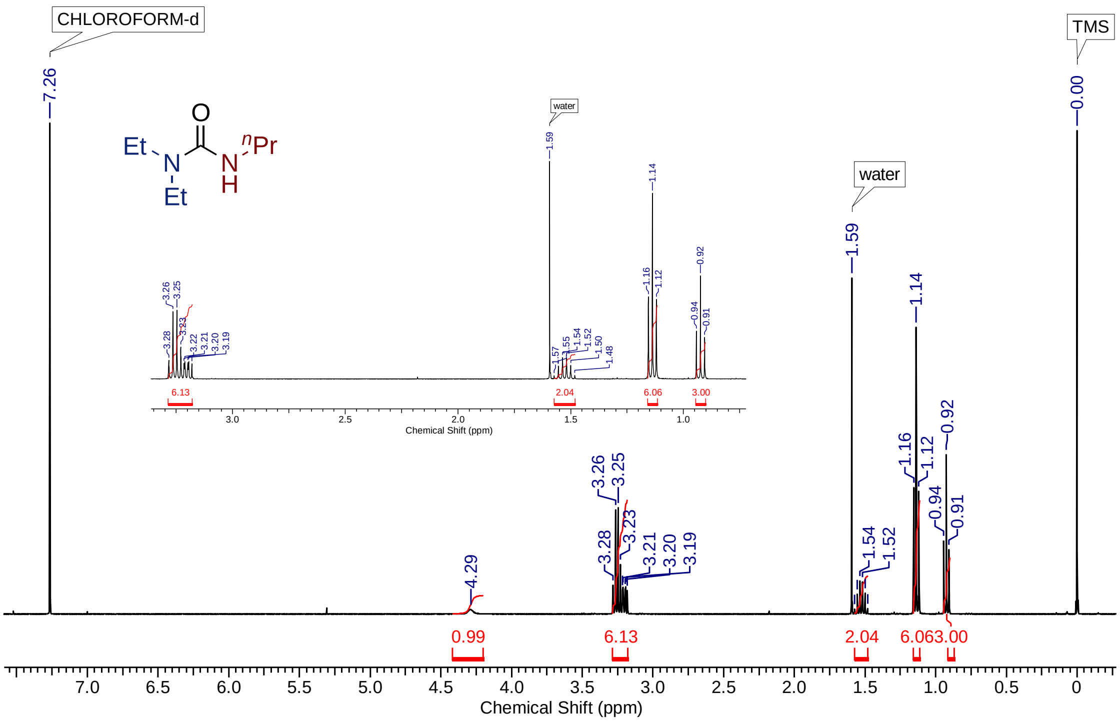

Figure S6-34. ^{1}H NMR spectrum of **24** in $CDCl_3$ (400 MHz).

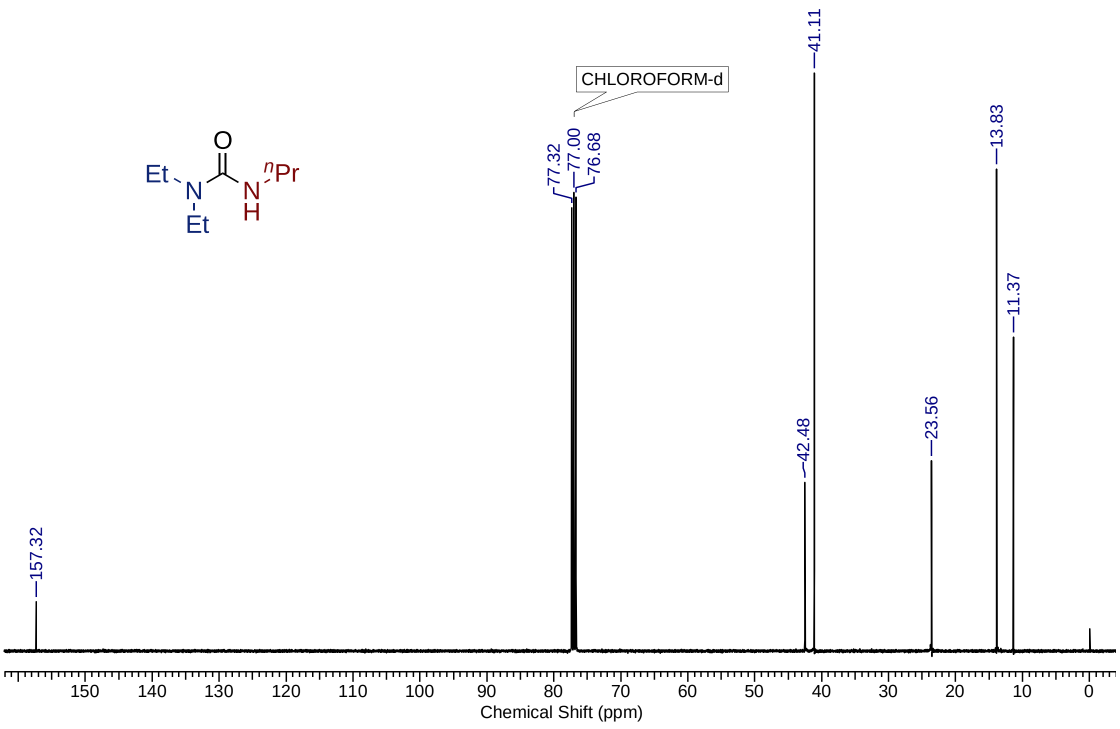

Figure S6-35. $^{13}C\{^{1}H\}$ NMR spectrum of **24** in $CDCl_3$ (101 MHz).

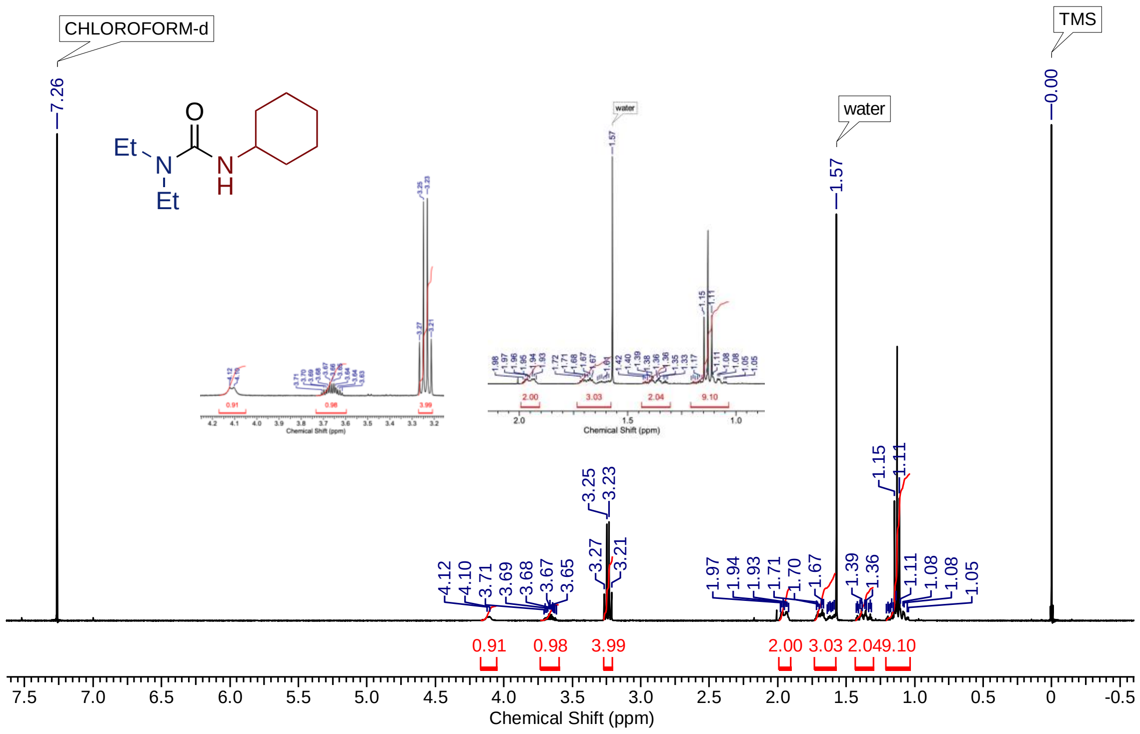

Figure S6-36. ^{1}H NMR spectrum of **25** in $CDCl_3$ (400 MHz).

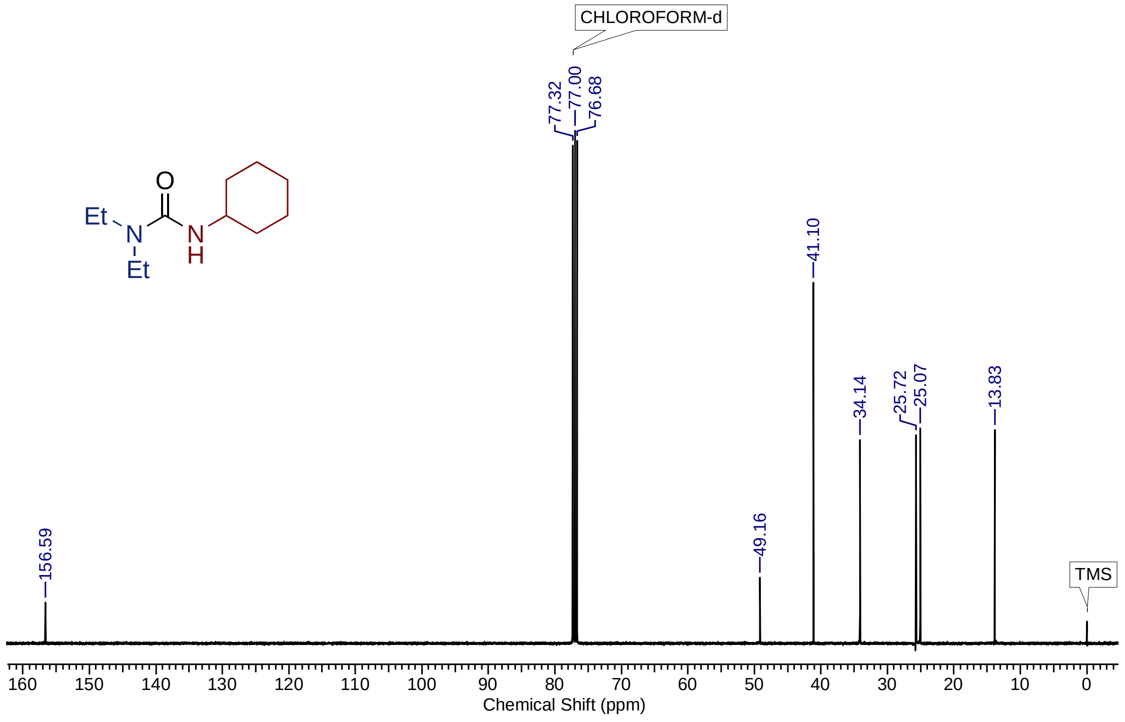

Figure S6-37. $^{13}C\{^{1}H\}$ NMR spectrum of **25** in $CDCl_3$ (101 MHz).

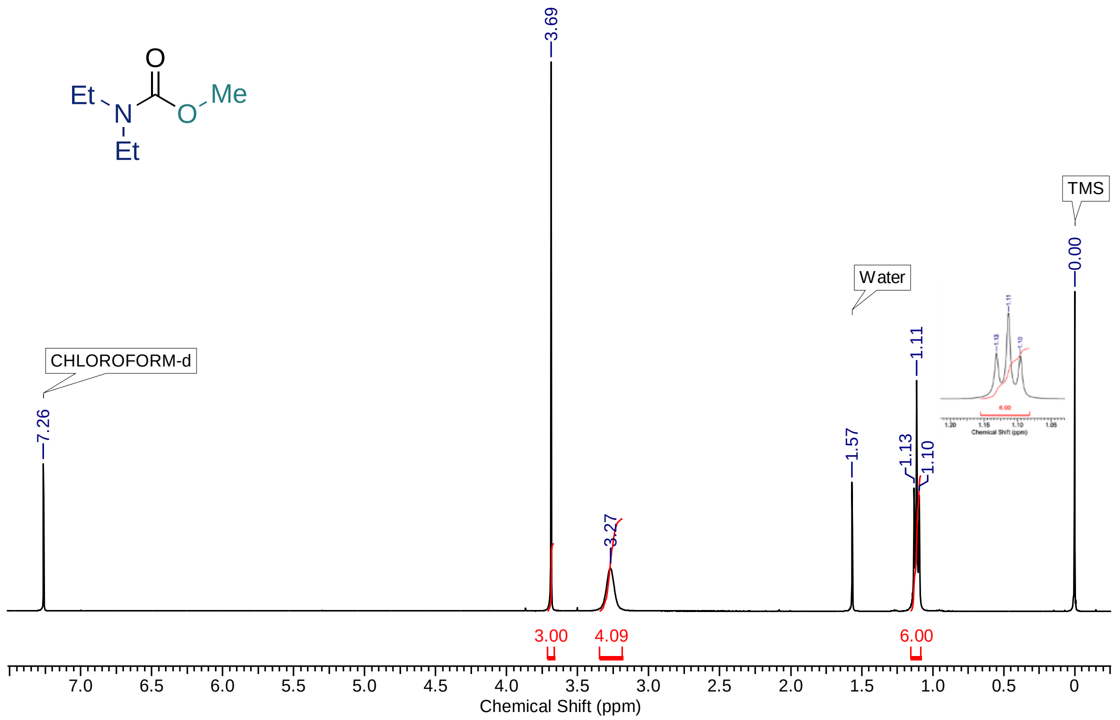

Figure S6-38. ^{1}H NMR spectrum of **26** in $CDCl_3$ (400 MHz).

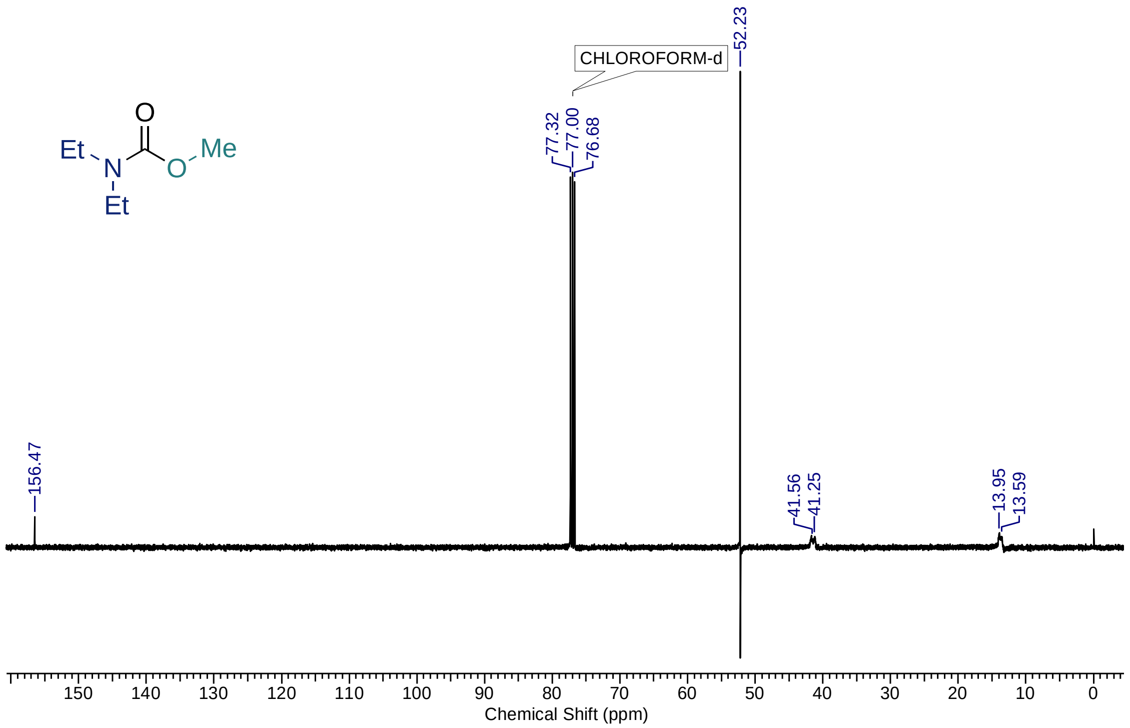

Figure S6-39. $^{13}C\{^{1}H\}$ NMR spectrum of **26** in $CDCl_3$ (101 MHz).

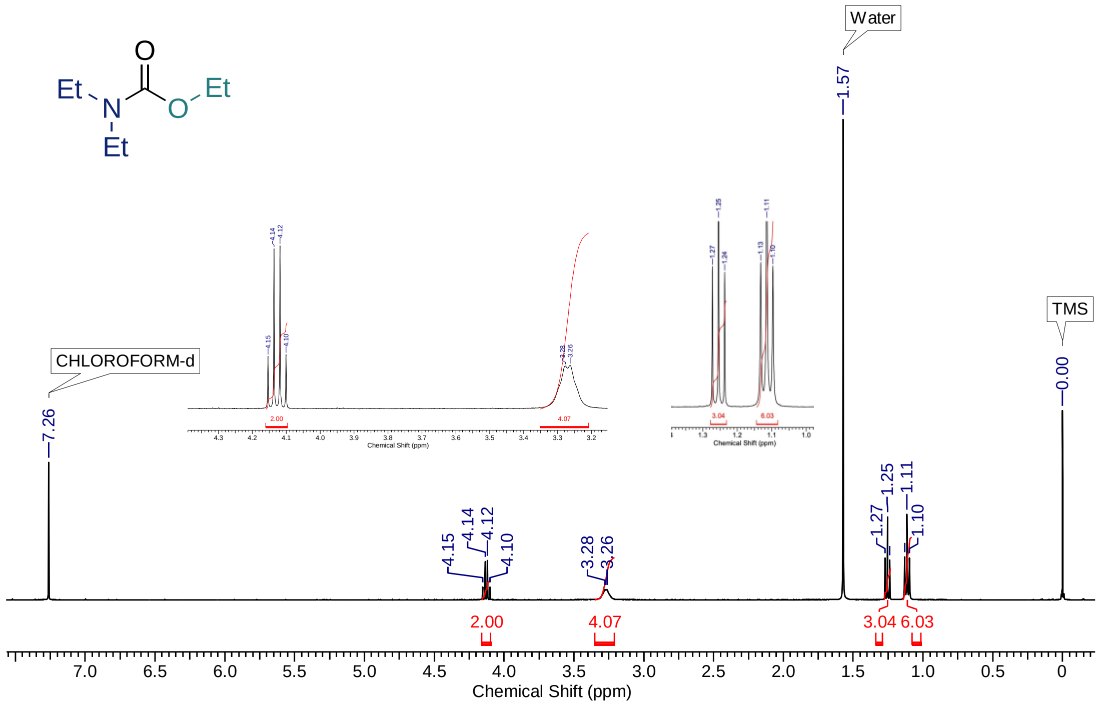

Figure S6-40. ^{1}H NMR spectrum of **27** in $CDCl_3$ (400 MHz).

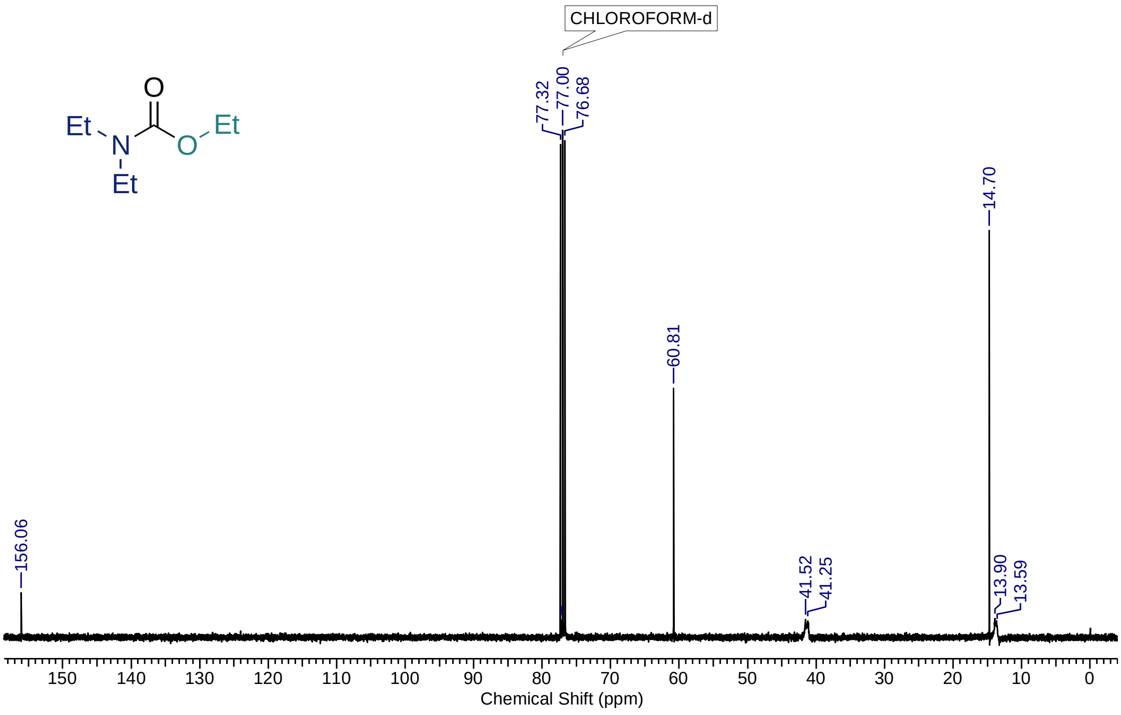

Figure S6-41. $^{13}C\{^{1}H\}$ NMR spectrum of **27** in $CDCl_3$ (101 MHz).

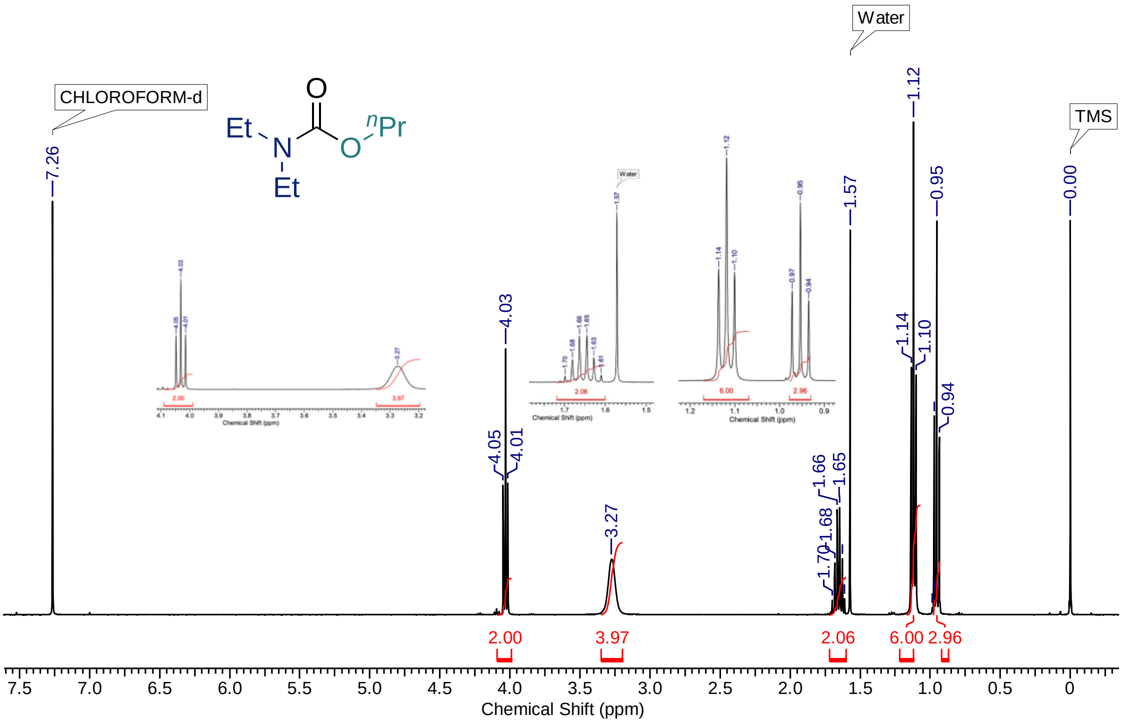

Figure S6-42. ^{1}H NMR spectrum of **28** in $CDCl_3$ (400 MHz).

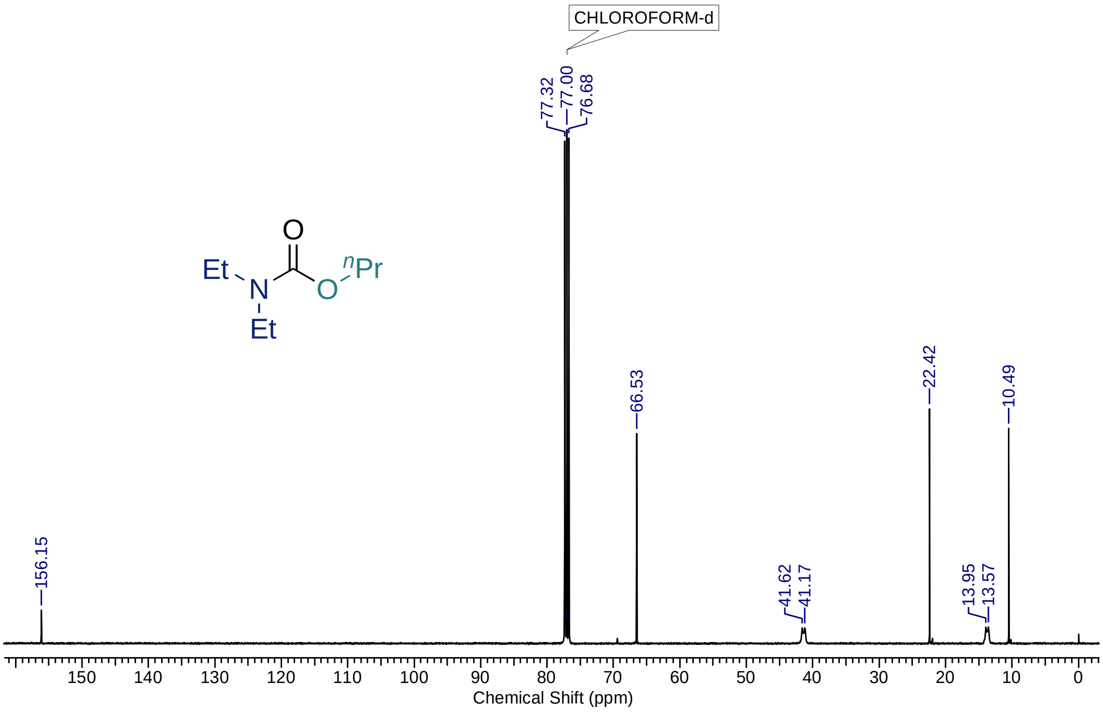

Figure S6-43. $^{13}C\{^{1}H\}$ NMR spectrum of **28** in $CDCl_3$ (101 MHz).

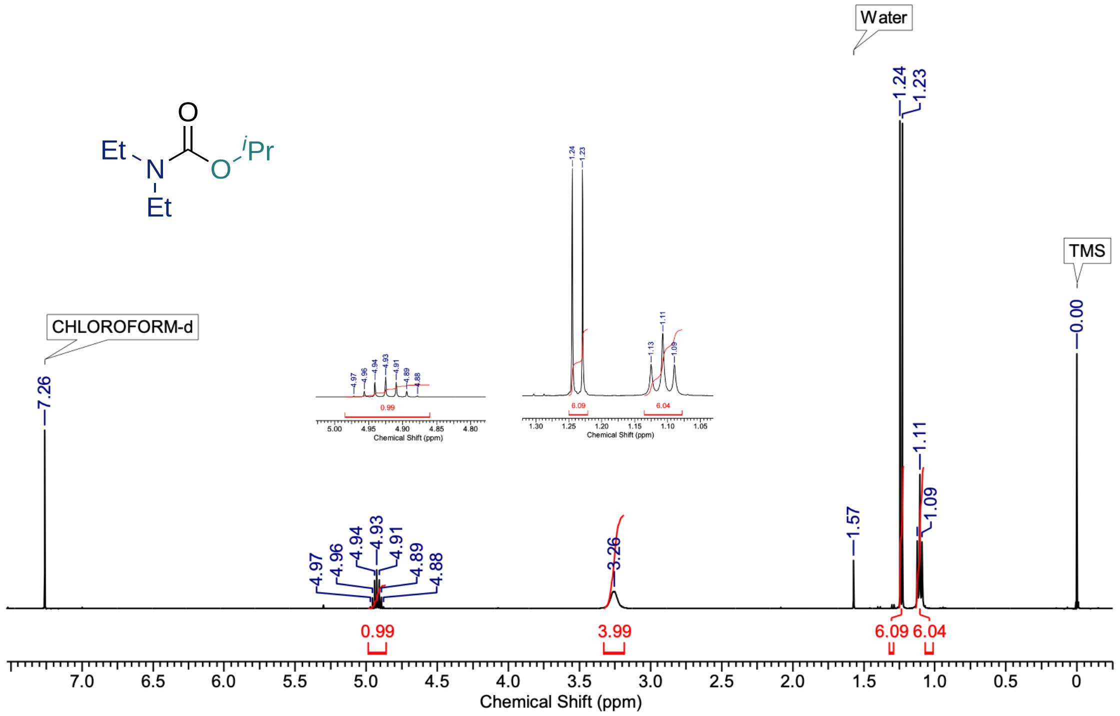

Figure S6-44. ^{1}H NMR spectrum of **29** in $CDCl_3$ (400 MHz).

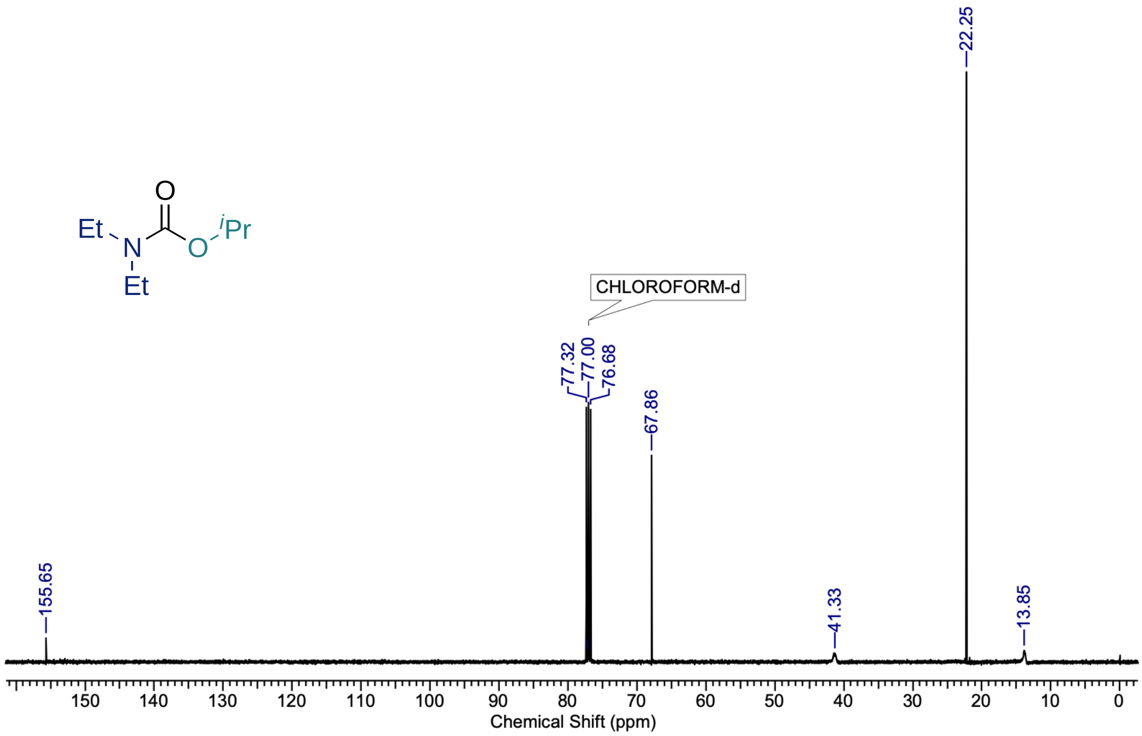

Figure S6-45. $^{13}C\{^{1}H\}$ NMR spectrum of **29** in $CDCl_3$ (101 MHz).

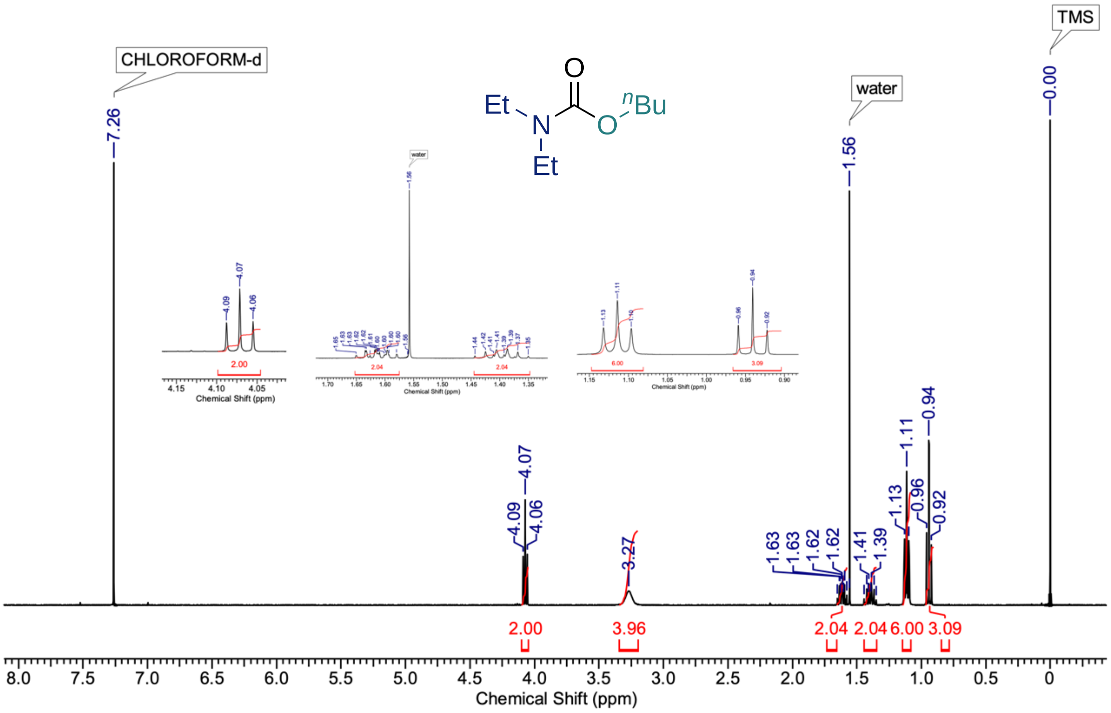

Figure S6-46. ^{1}H NMR spectrum of **30** in $CDCl_3$ (400 MHz).

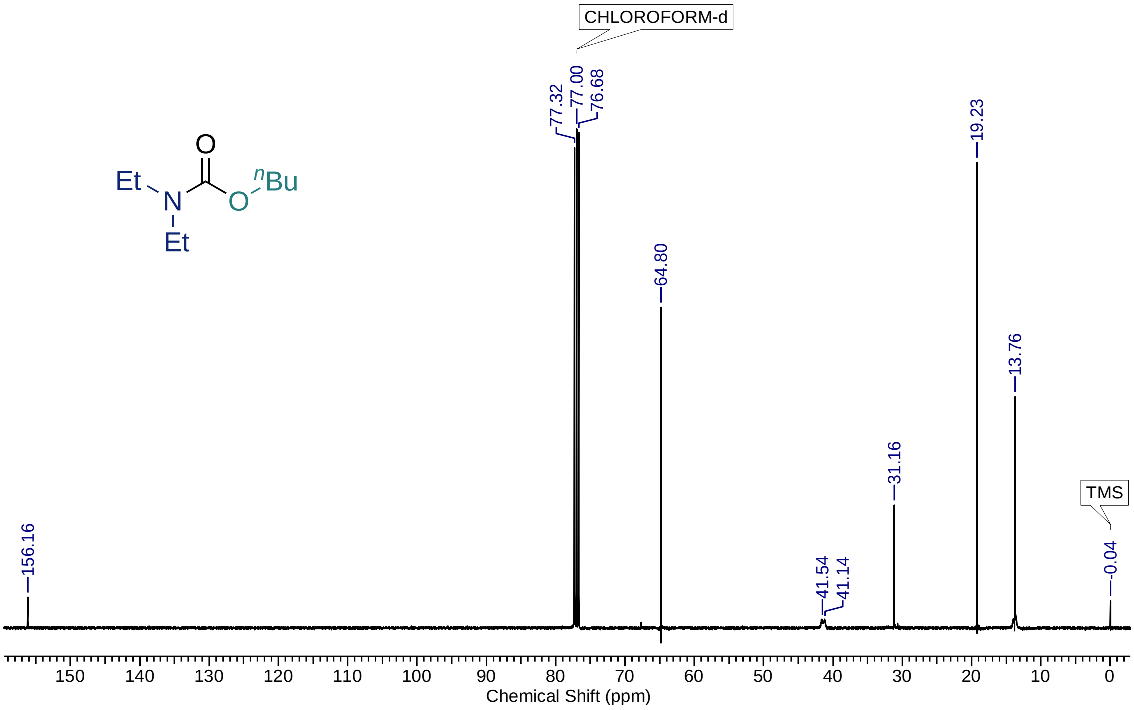

Figure S6-47. $^{13}C\{^{1}H\}$ NMR spectrum of **30** in $CDCl_3$ (101 MHz).

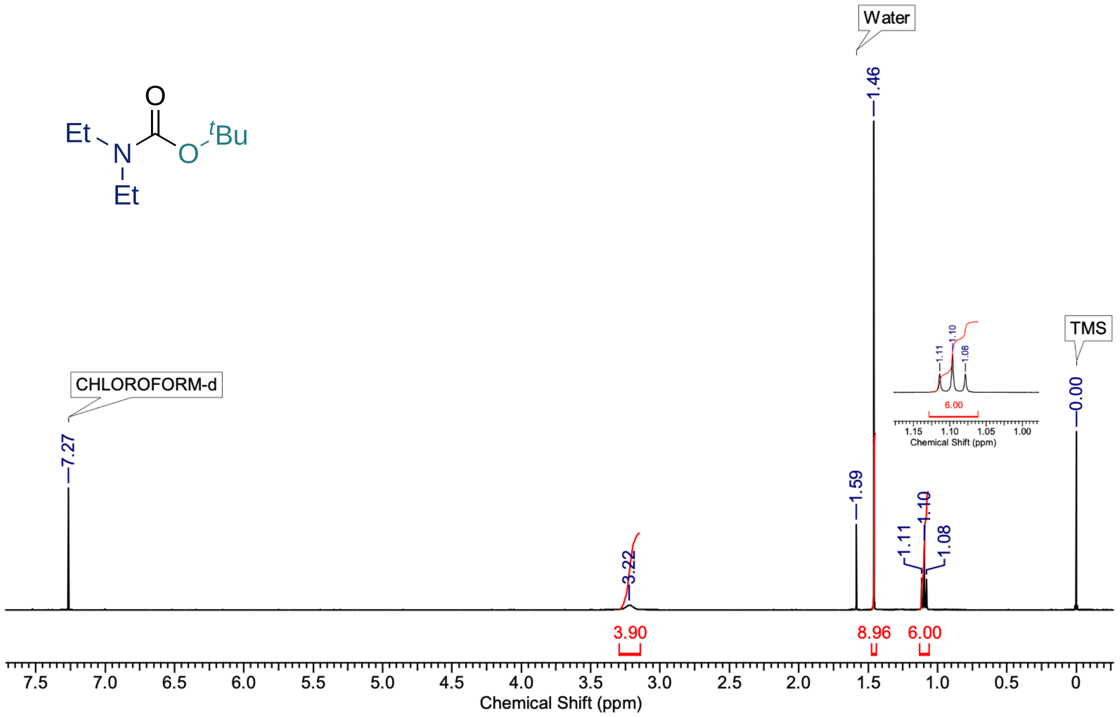

Figure S6-48. ^{1}H NMR spectrum of **31** in $CDCl_3$ (400 MHz).

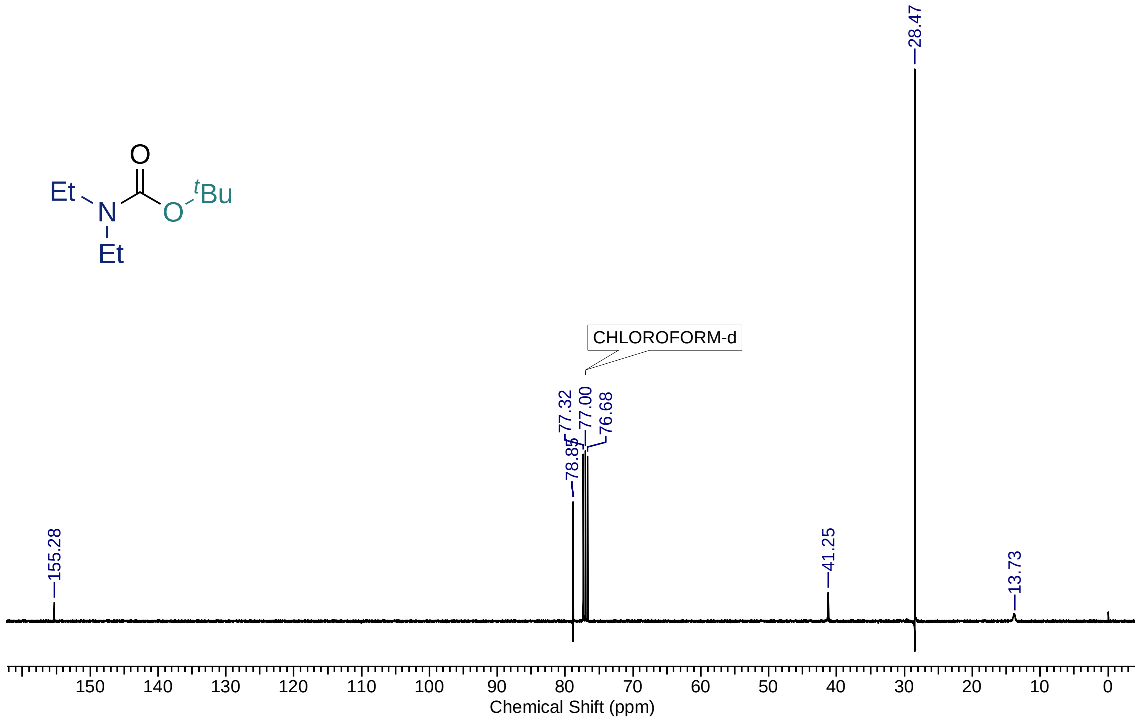

Figure S6-49. $^{13}C\{^1H\}$ NMR spectrum of **31** in $CDCl_3$ (101 MHz).

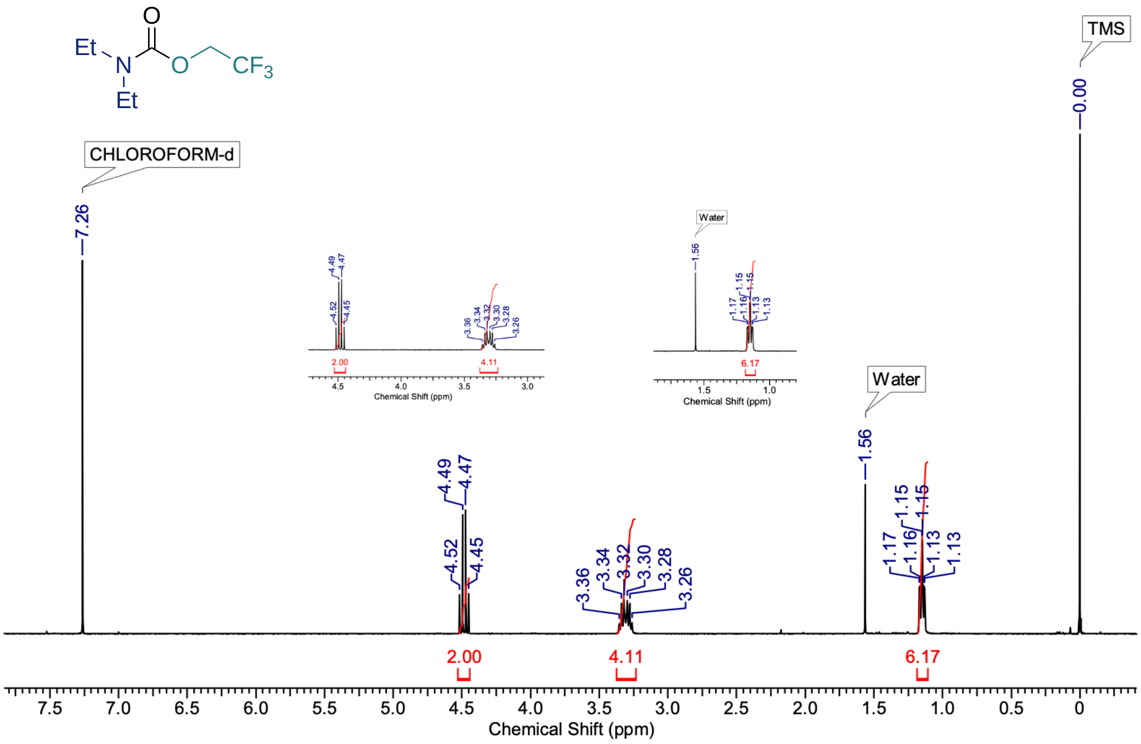

Figure S6-50. ^{1}H NMR spectrum of **32** in $CDCl_3$ (400 MHz).

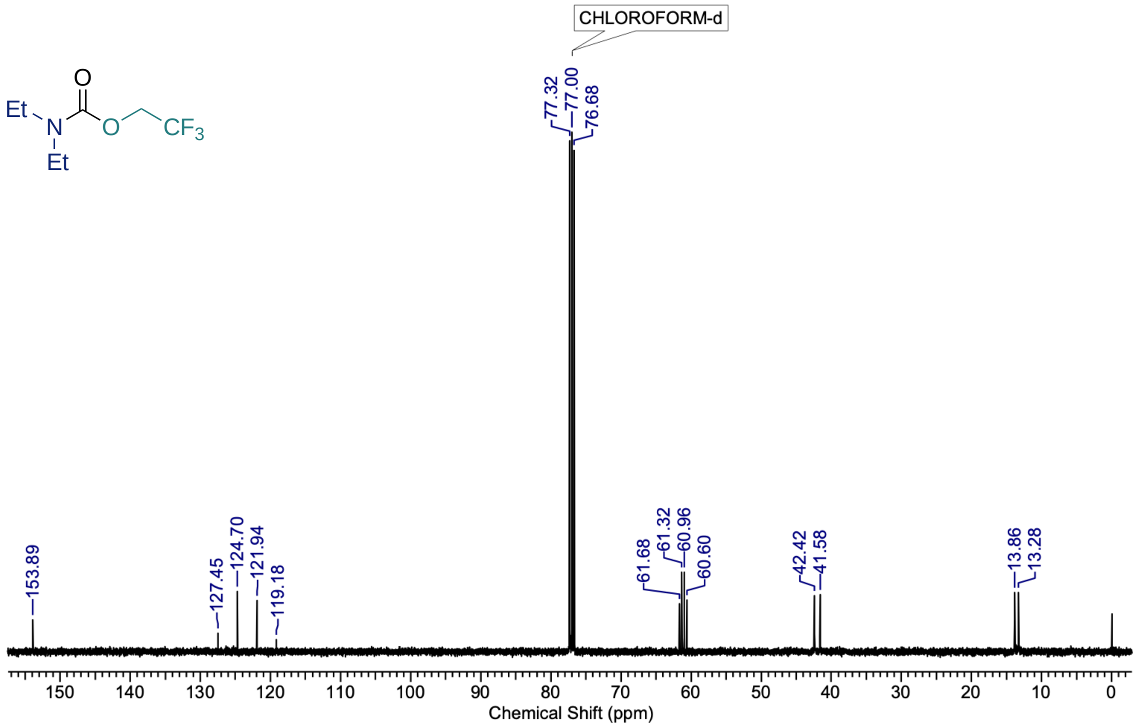

Figure S6-51. $^{13}C\{^{1}H\}$ NMR spectrum of **32** in $CDCl_3$ (101 MHz).

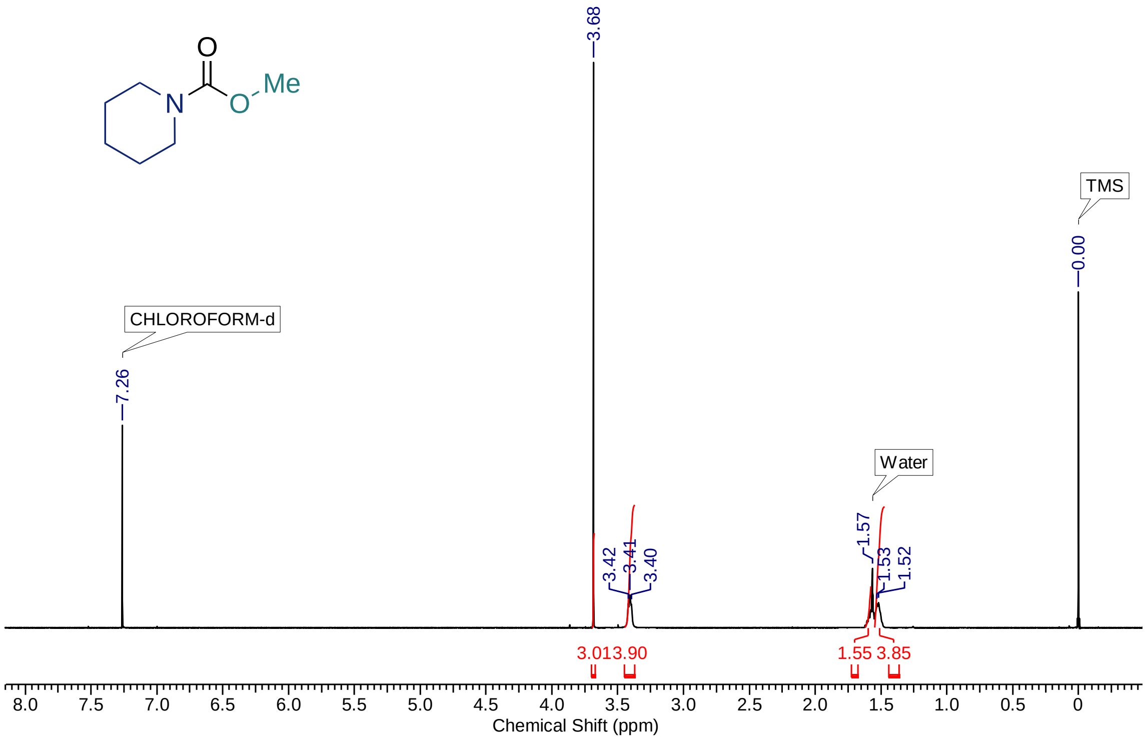

Figure S6-52. ^{1}H NMR spectrum of **33** in $CDCl_3$ (400 MHz).

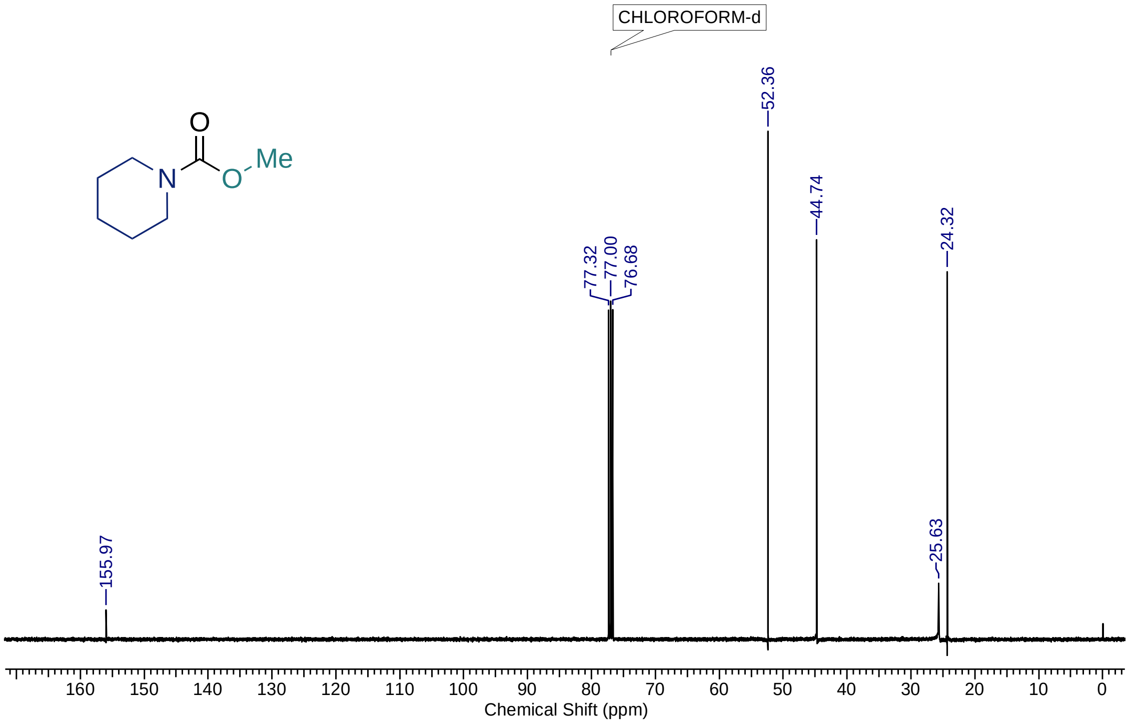

Figure S6-53. $^{13}C\{^{1}H\}$ NMR spectrum of **33** in $CDCl_3$ (101 MHz).

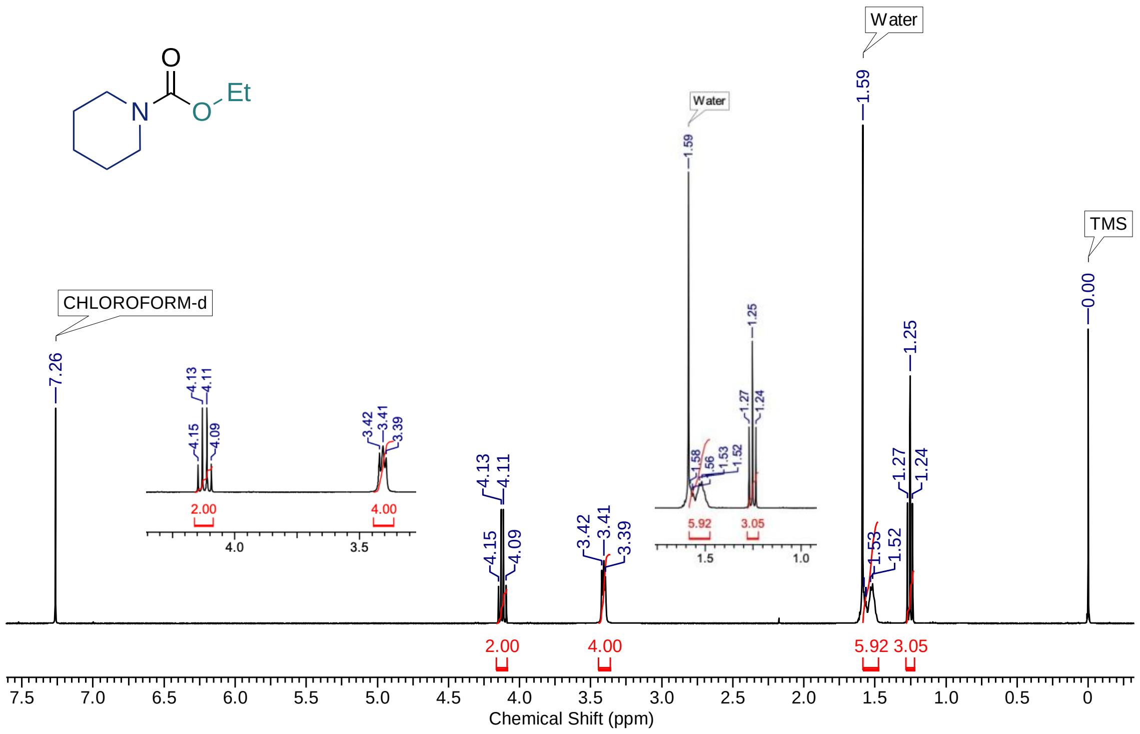

Figure S6-54. ^{1}H NMR spectrum of **34** in $CDCl_3$ (400 MHz).

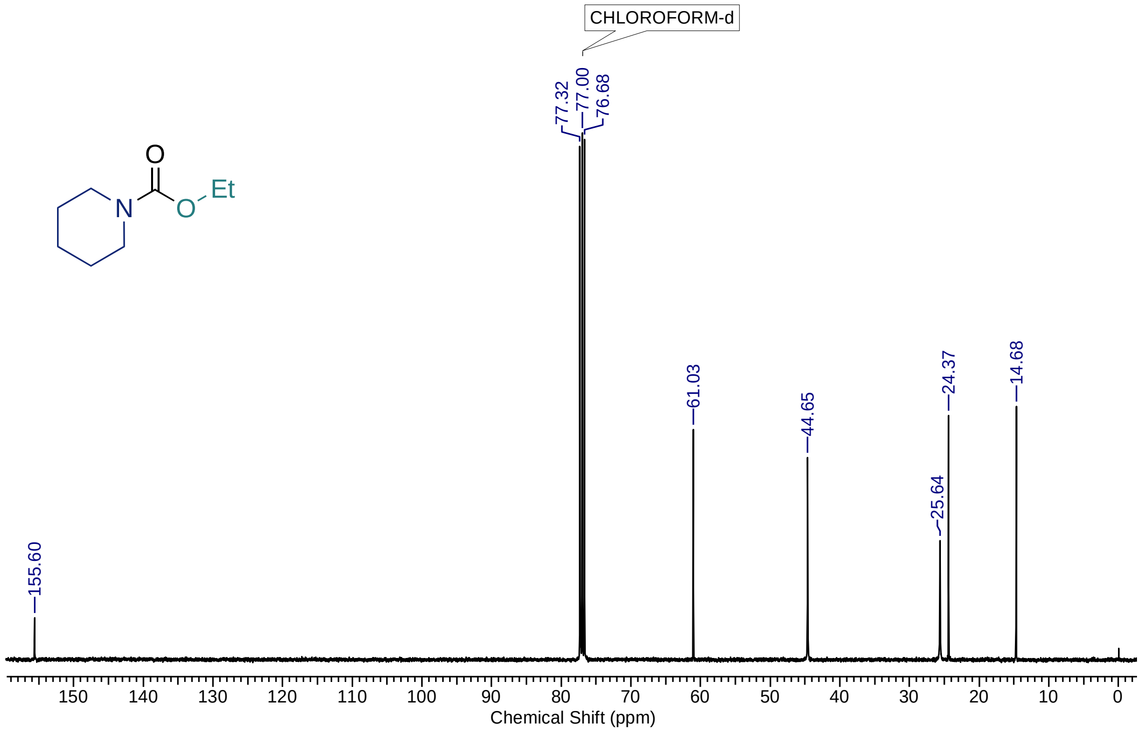

Figure S6-55. $^{13}C\{^{1}H\}$ NMR spectrum of **34** in $CDCl_3$ (101 MHz).

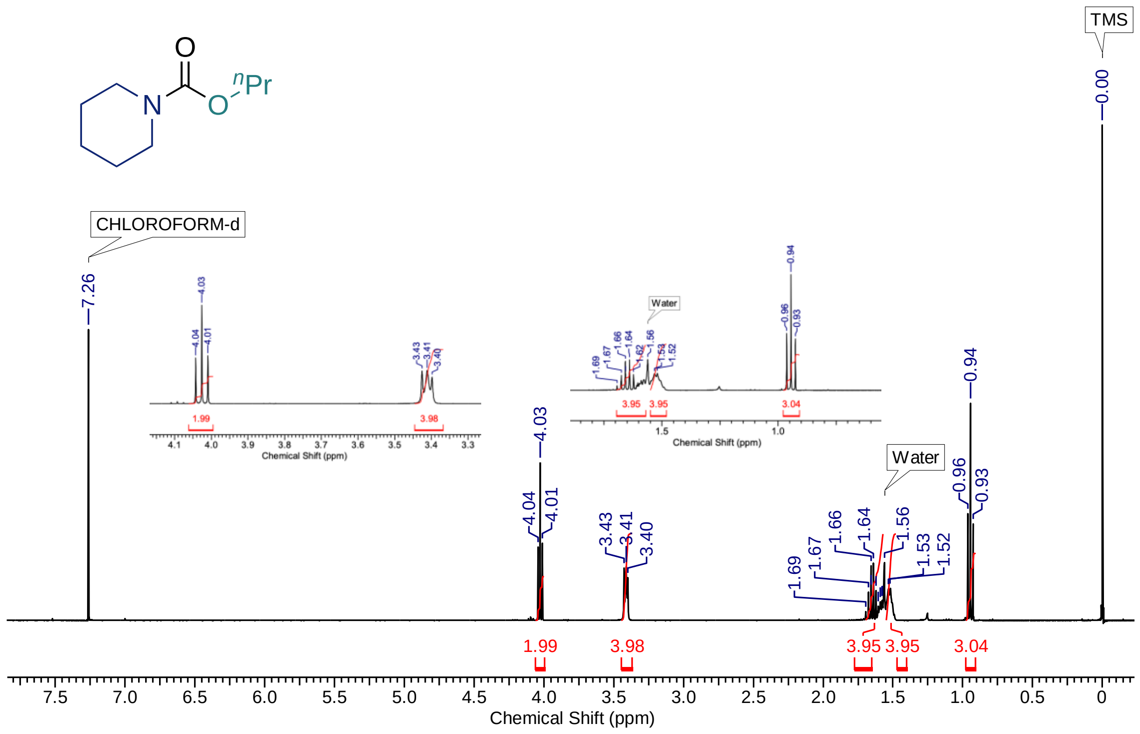

Figure S6-56. ^{1}H NMR spectrum of **35** in $CDCl_3$ (400 MHz).

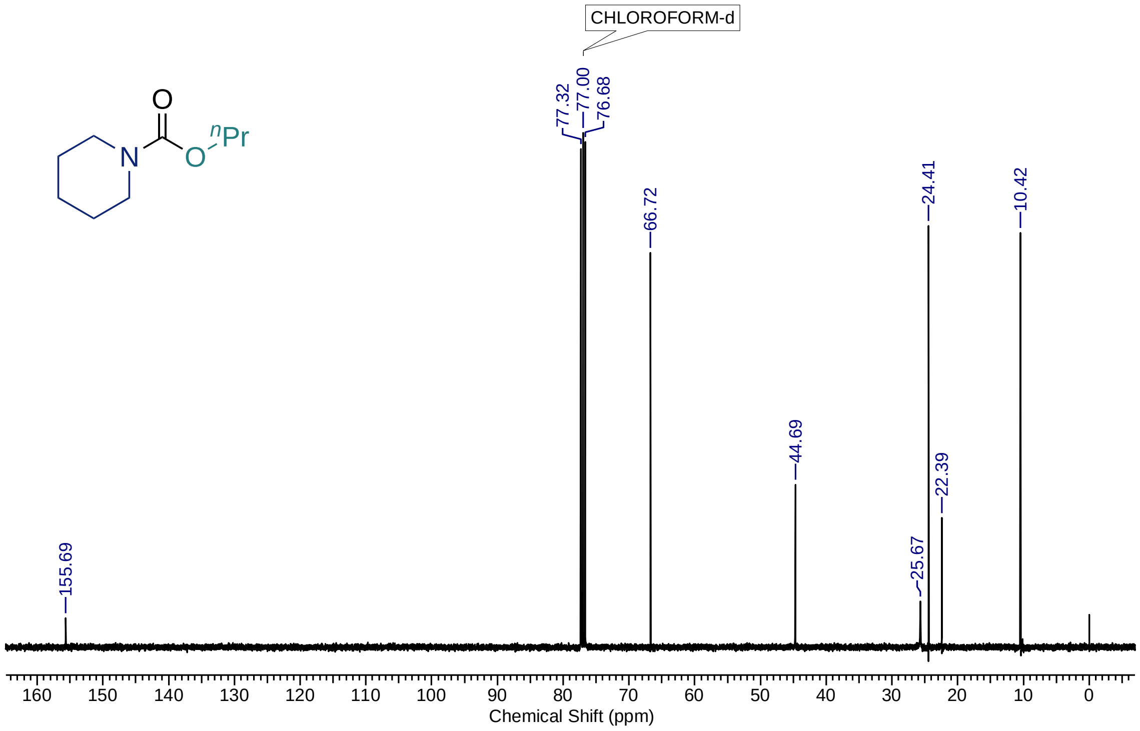

Figure S6-57. $^{13}C\{^{1}H\}$ NMR spectrum of **35** in $CDCl_3$ (101 MHz).

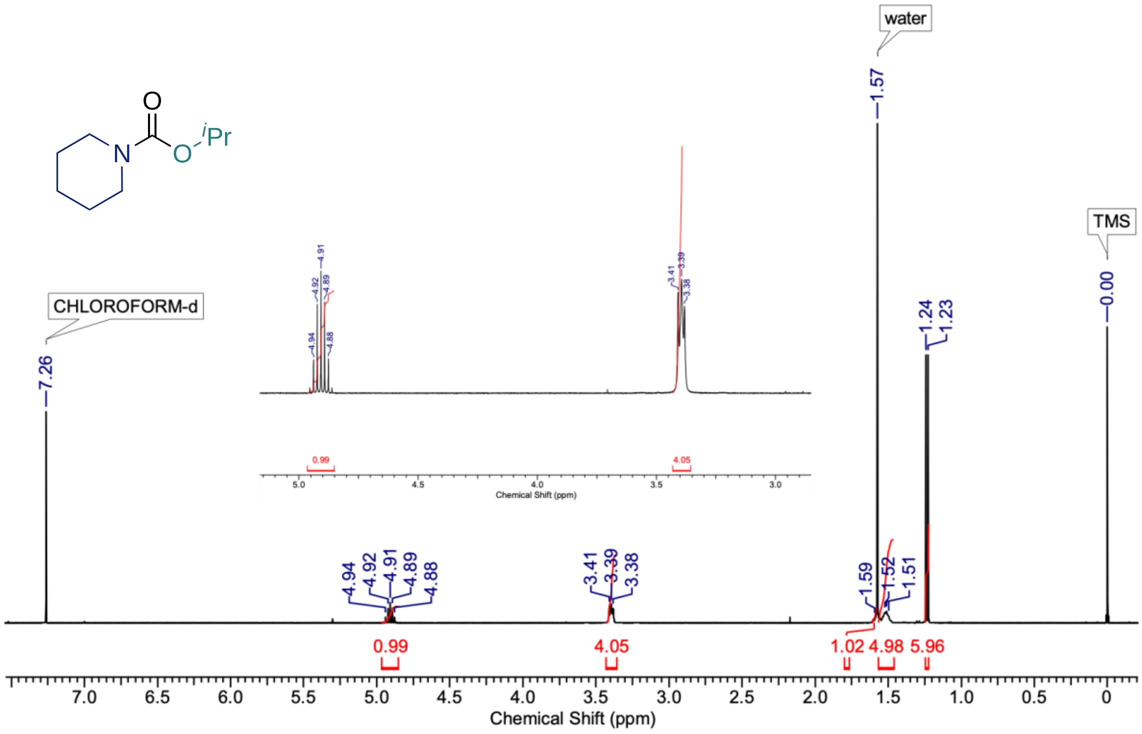

Figure S6-58. ^{1}H NMR spectrum of **36** in $CDCl_3$ (400 MHz).

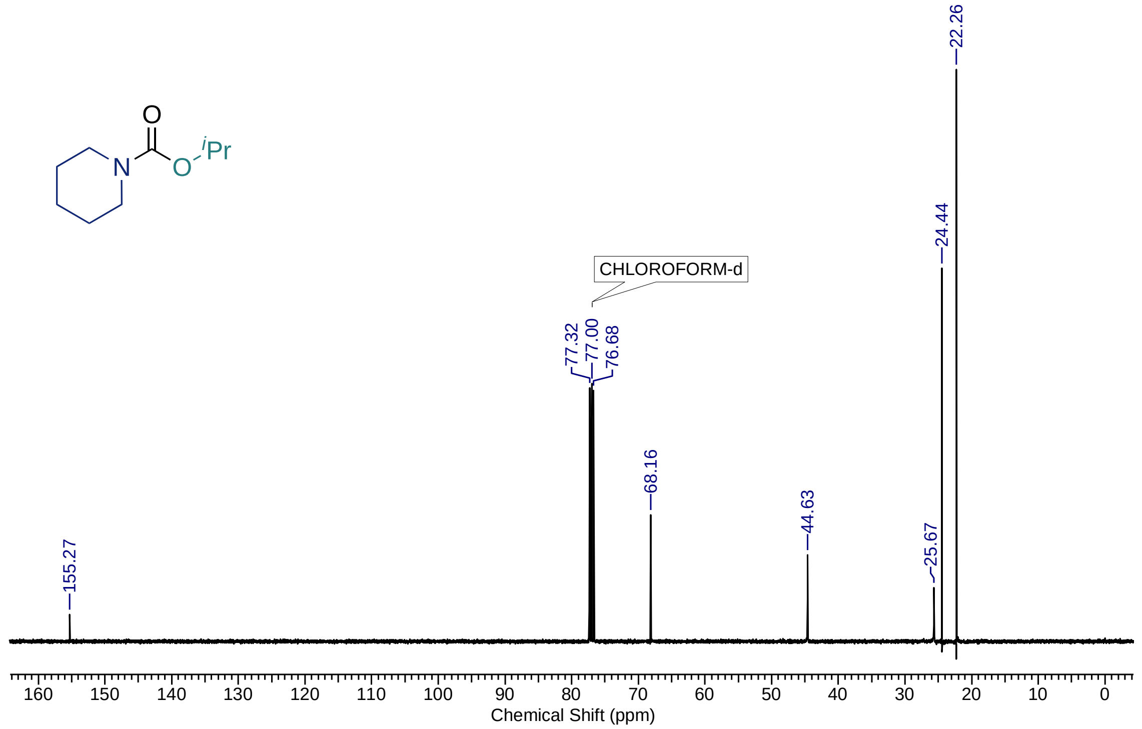

Figure S6-59. $^{13}C\{^{1}H\}$ NMR spectrum of **36** in $CDCl_3$ (101 MHz).

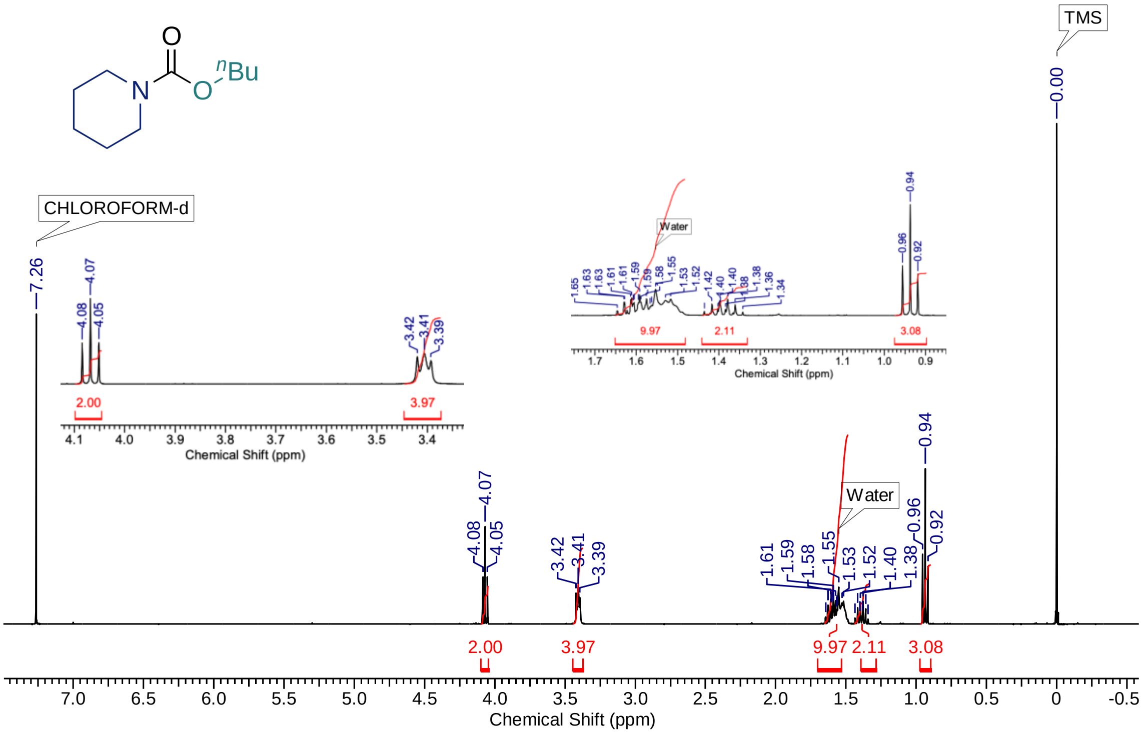

Figure S6-60. ^{1}H NMR spectrum of **37** in $CDCl_3$ (400 MHz).

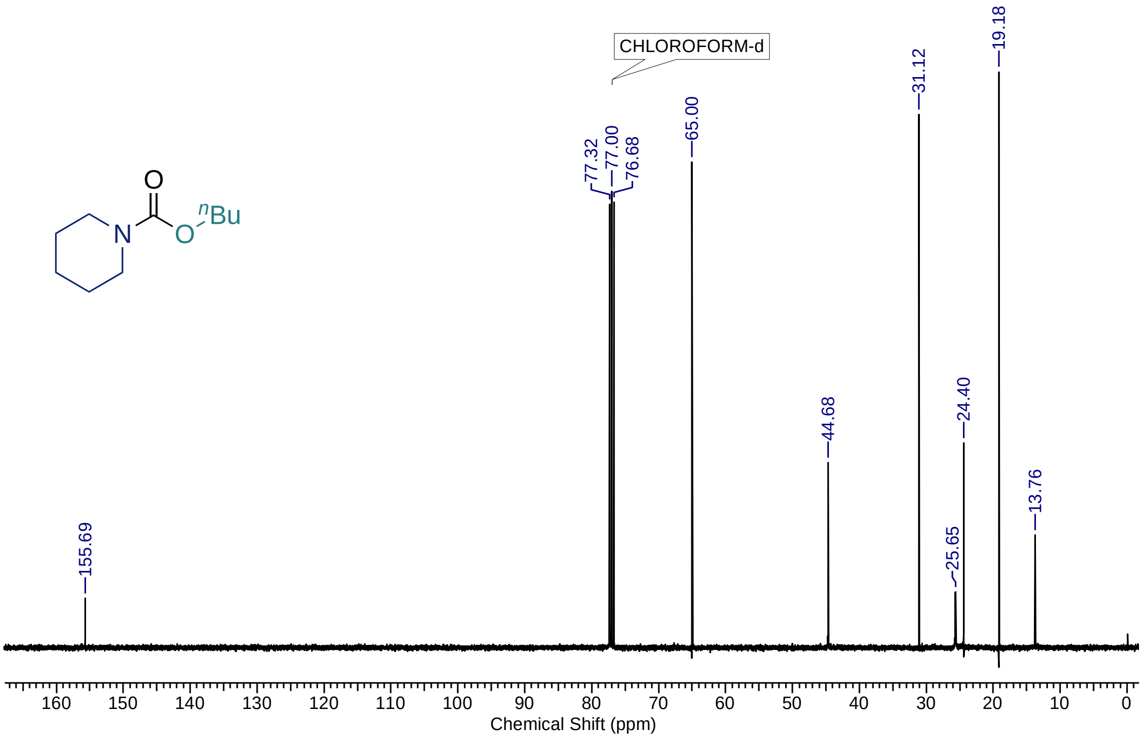

Figure S6-61. $^{13}C\{^{1}H\}$ NMR spectrum of **37** in $CDCl_3$ (101 MHz).

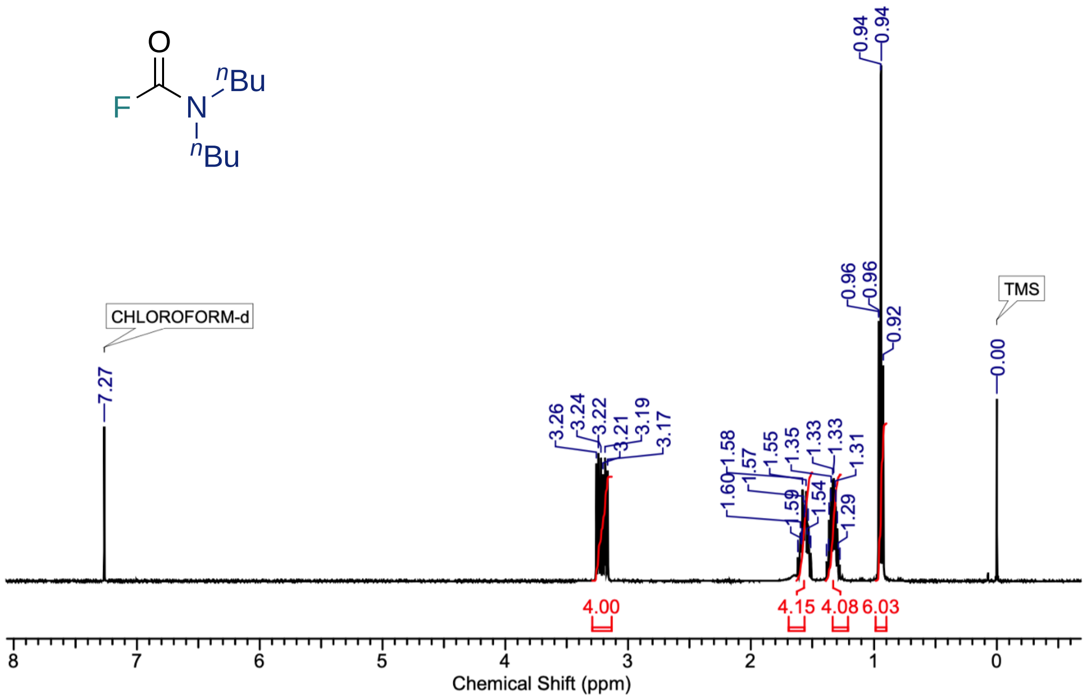

Figure S6-62. ^{1}H NMR spectrum of **40** in $CDCl_3$ (400 MHz).

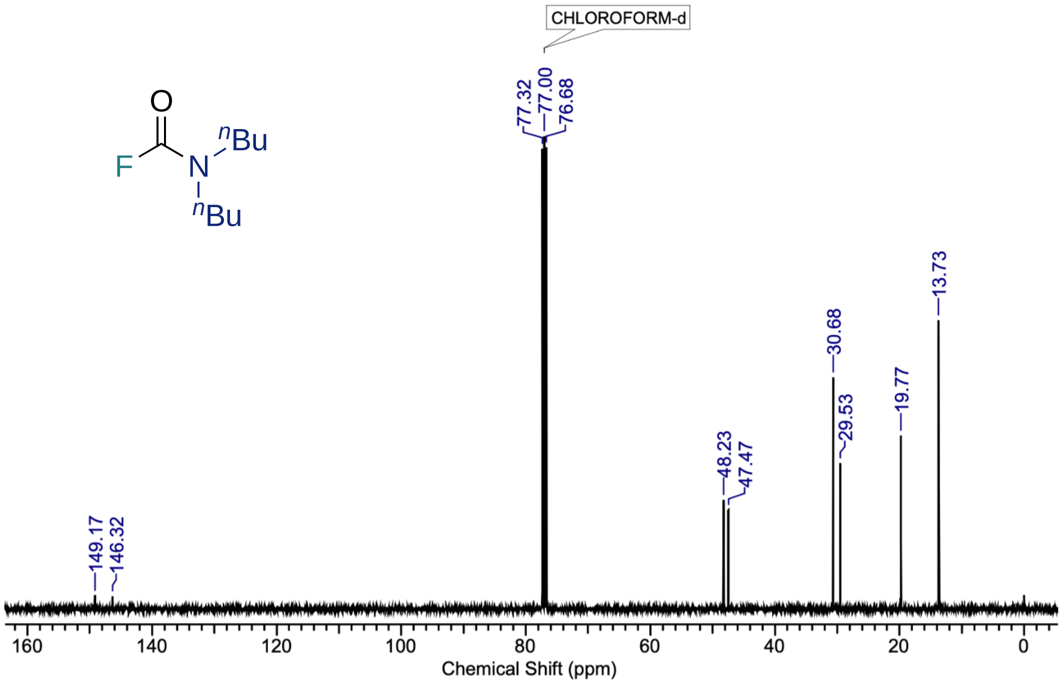

Figure S6-63. ^{13}C{^{1}H} NMR spectrum of **40** in $CDCl_3$ (101 MHz).

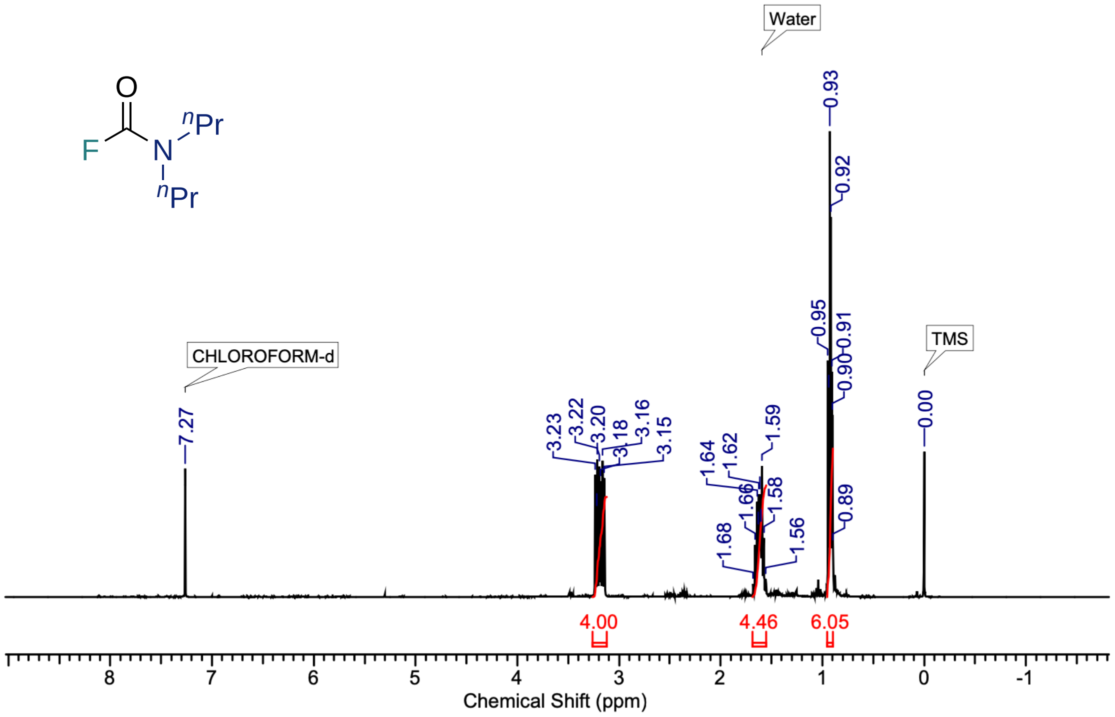

Figure S6-64. ^{1}H NMR spectrum of **41** in $CDCl_3$ (400 MHz).

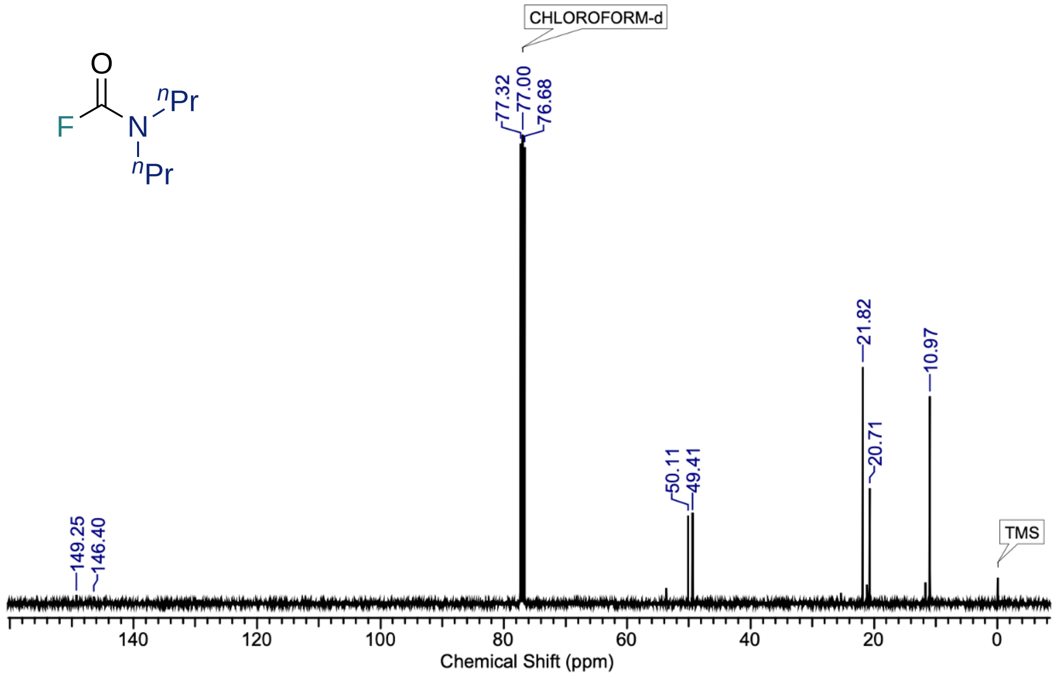

Figure S6-65. $^{13}C\{^{1}H\}$ NMR spectrum of **41** in $CDCl_3$ (101 MHz).

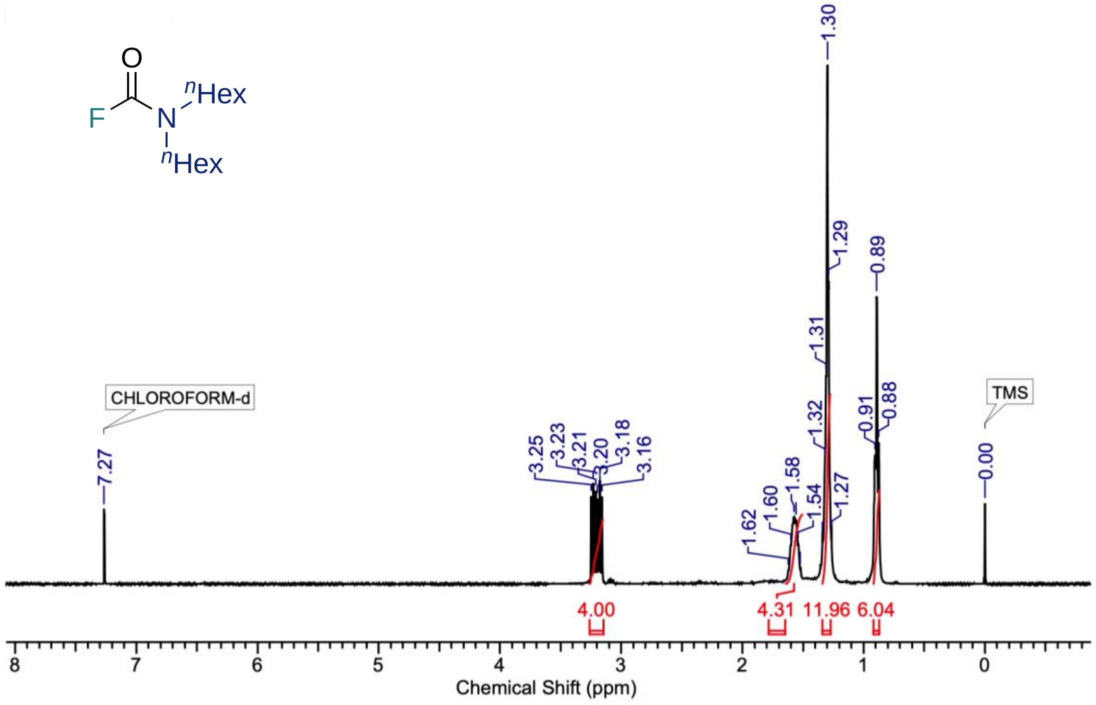

Figure S6-66. ^{1}H NMR spectrum of **42** in $CDCl_3$ (400 MHz).

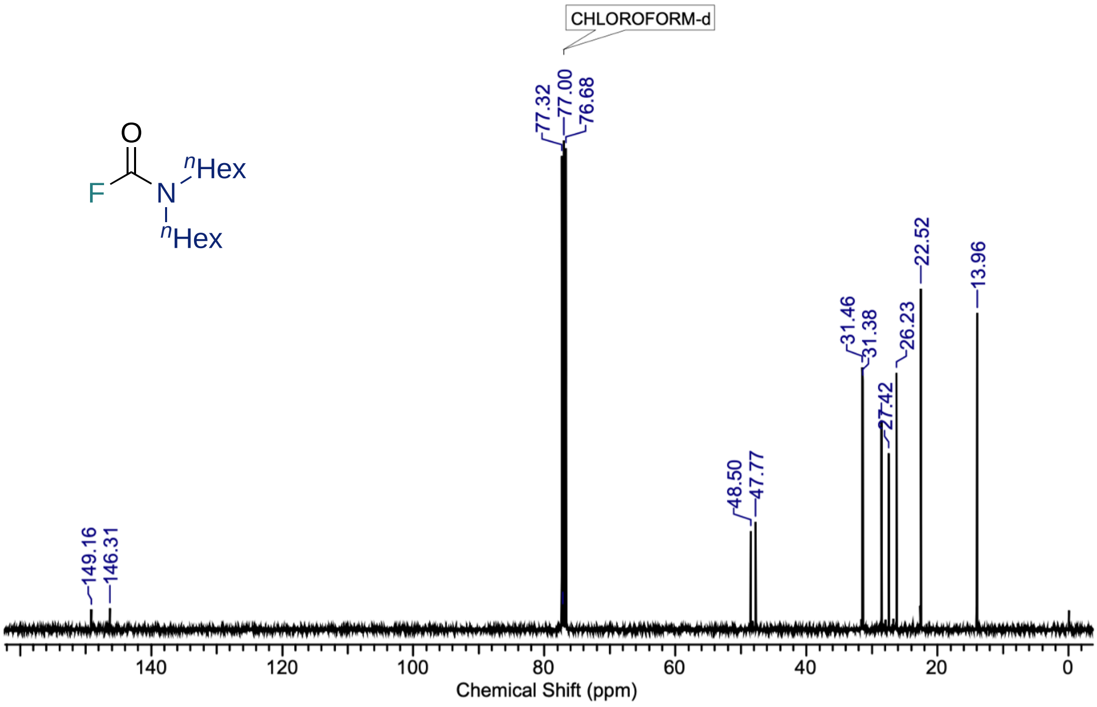

Figure S6-67. $^{13}C\{^1H\}$ NMR spectrum of **42** in $CDCl_3$ (101 MHz).

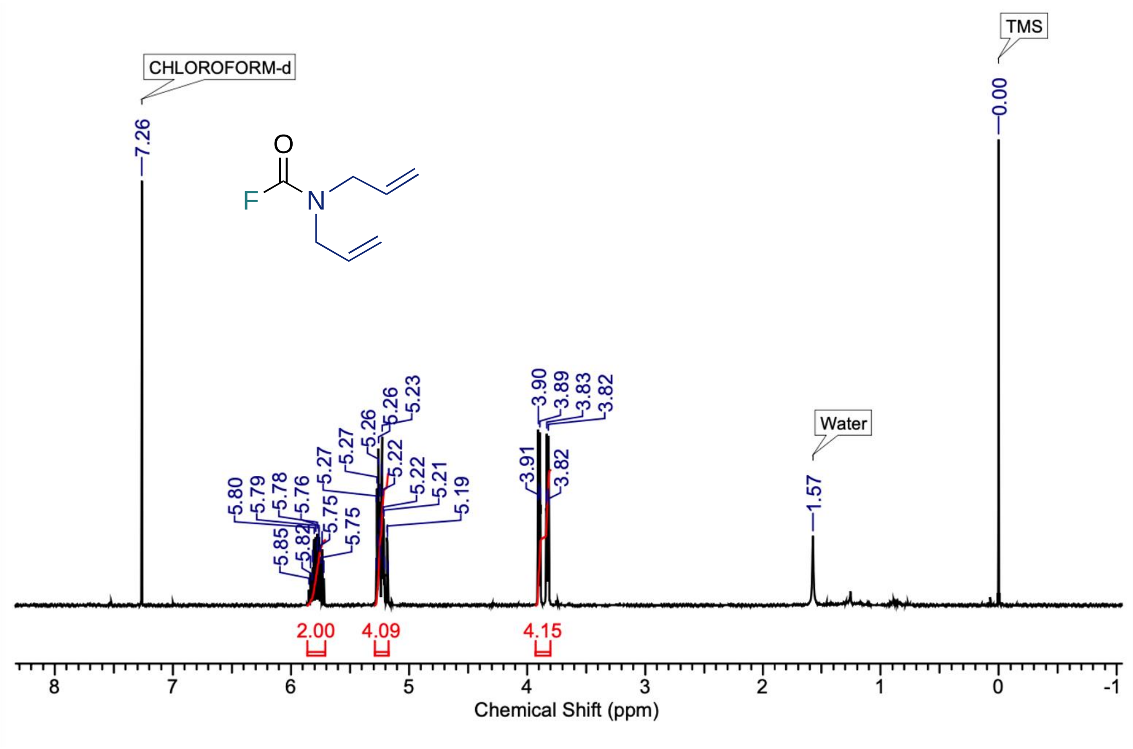

Figure S6-68. ^{1}H NMR spectrum of **43** in $CDCl_3$ (400 MHz).

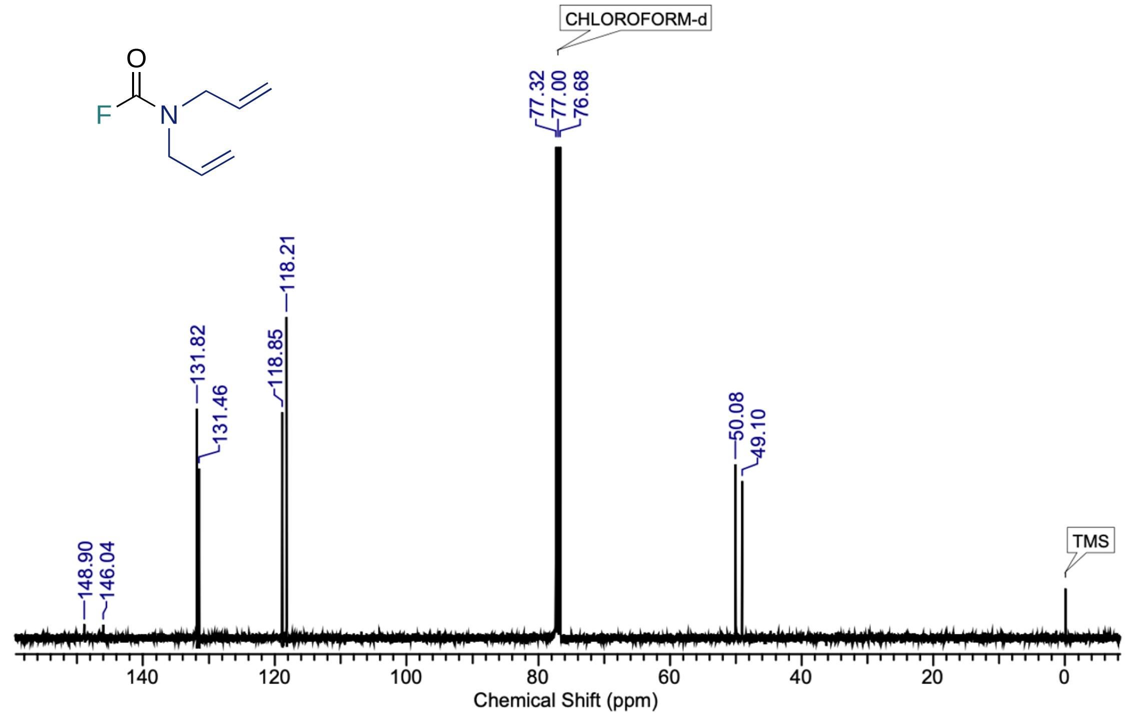

Figure S6-69. $^{13}C\{^{1}H\}$ NMR spectrum of **43** in $CDCl_3$ (101 MHz).

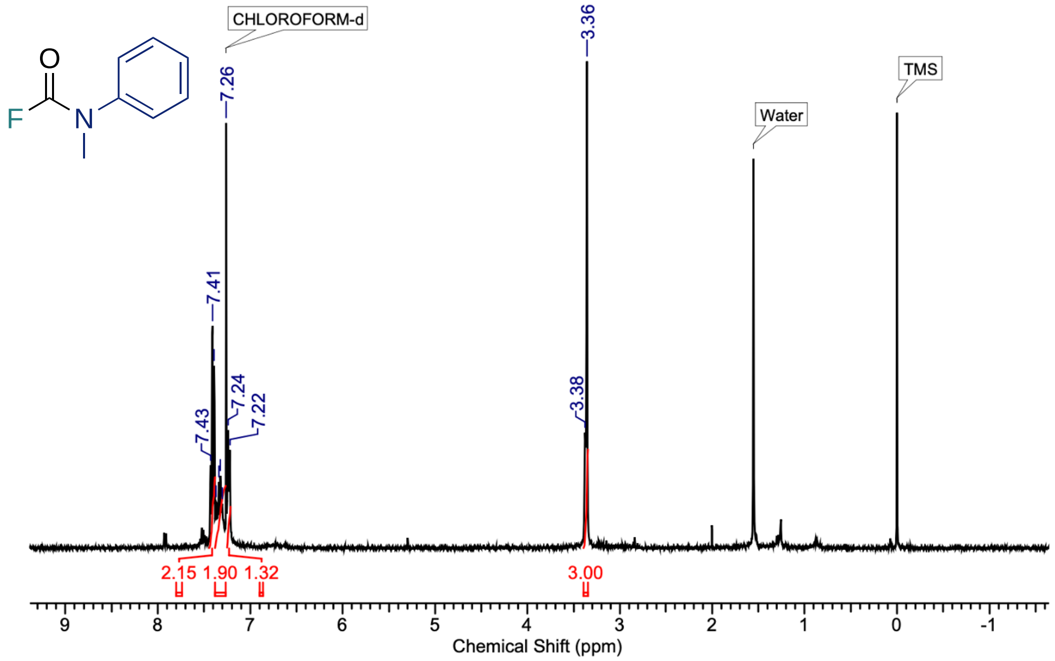

Figure S6-70. ^{1}H NMR spectrum of **44** in $CDCl_3$ (400 MHz).

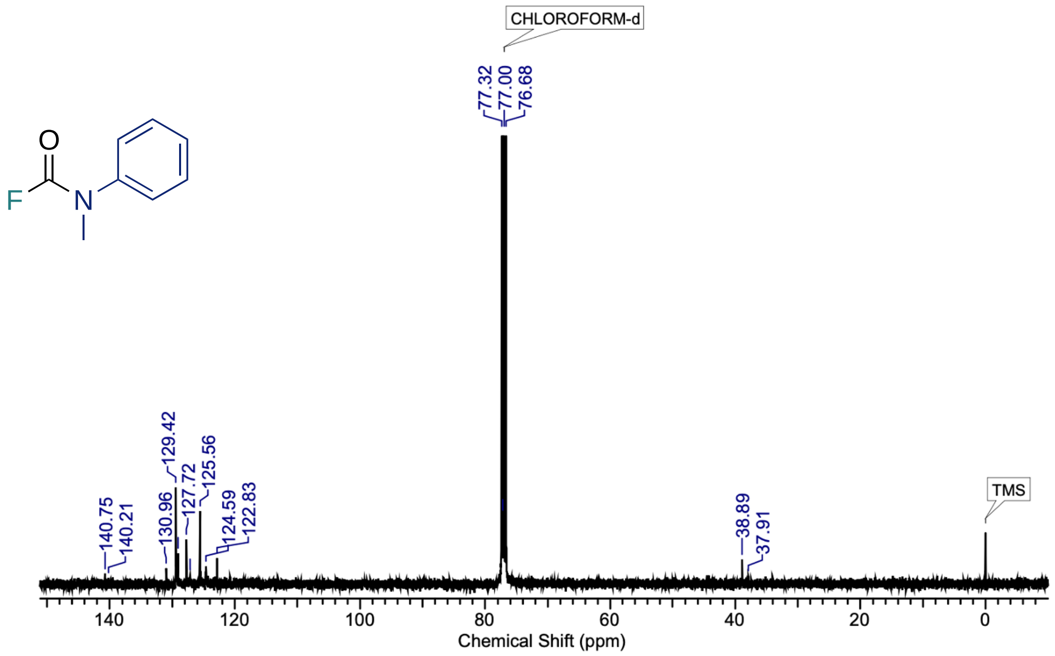

Figure S6-71. $^{13}C\{^{1}H\}$ NMR spectrum of **44** in $CDCl_3$ (101 MHz).

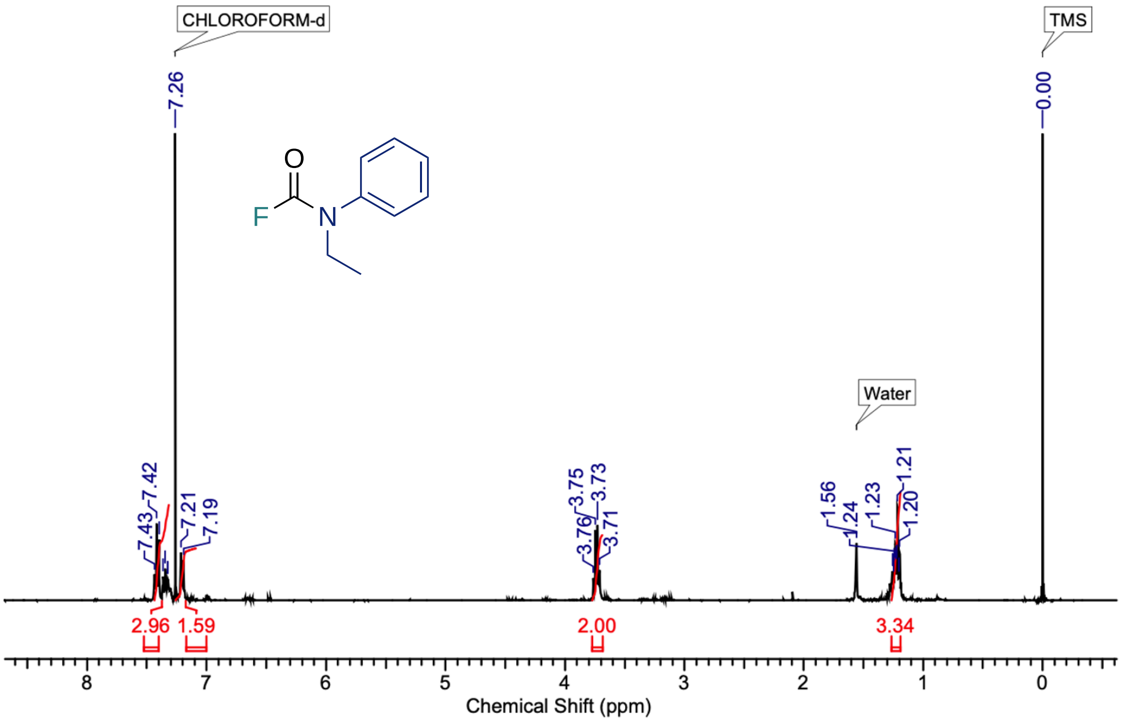

Figure S6-72. ^{1}H NMR spectrum of **45** in $CDCl_3$ (400 MHz).

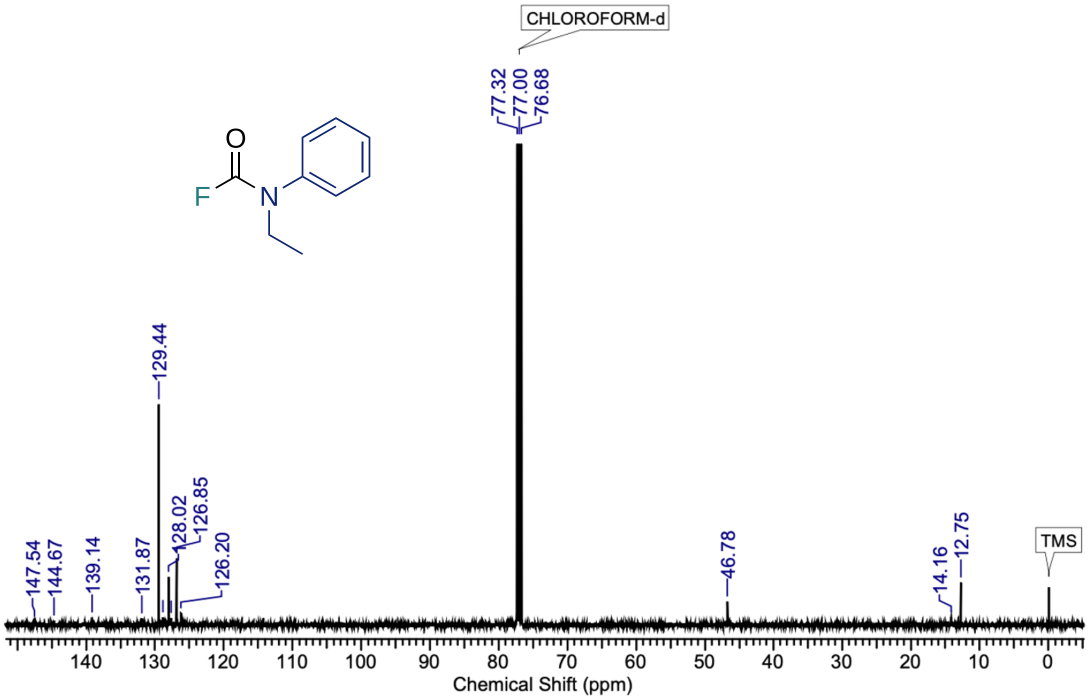

Figure S6-73. $^{13}C\{^{1}H\}$ NMR spectrum of **45** in $CDCl_3$ (101 MHz).

7. DFT Calculations

Cartesian coordinates of the optimized structures for the reaction of phosgene and nucleophiles. Geometry optimizations were performed at the B3LYP/6-31G* level of theory.

E(RB3LYP) = -1188.753494

Atom	X	Y	Z
C	1.198447	0.134972	0.513589
O	0.909774	0.008017	1.65307
C	-2.673915	-0.711315	-0.475609
H	-2.525761	-0.597929	-1.554257
H	-3.358822	-1.561561	-0.329197
C	-3.273302	0.560207	0.116741
H	-2.605591	1.412473	-0.045864
H	-4.24397	0.784247	-0.341551
H	-3.433468	0.454119	1.19764
Cl	0.918568	1.613724	-0.411636
Cl	2.01704	-1.098359	-0.4403
O	-1.382088	-1.01271	0.04917
H	-1.466592	-1.11818	1.009891

E(RB3LYP) = -1188.711501

Atom	X	Y	Z
C	-0.791993	-0.353999	0.617933
O	-1.303651	-0.341059	1.673034
C	1.777645	-0.517959	-0.286845
H	1.765375	-1.589053	-0.08382
H	1.437688	-0.329154	-1.307739
C	3.126047	0.112449	0.007162
H	3.424839	-0.062666	1.044284
H	3.876671	-0.335278	-0.653255
H	3.110373	1.190664	-0.181518
Cl	-1.116358	-1.437979	-0.717825
Cl	-0.929954	1.838443	-0.440627
O	0.795318	0.070631	0.641707
H	0.568841	1.038096	0.328297

E(RB3LYP) = -1188.782052

Atom	X	Y	Z
C	-0.437486	0.755836	-0.000216
O	0.551439	1.434144	-0.000319
C	-1.620465	-1.397149	-0.000138
H	-2.208367	-1.158091	0.889659
H	-2.208454	-1.158541	-0.890006
C	-1.14077	-2.834847	0.000201
H	-0.538747	-3.046042	0.888566
H	-2.008322	-3.503069	0.000397
H	-0.538798	-3.046488	-0.888092
Cl	-2.059034	1.52664	0.000253
Cl	3.435077	-0.043331	0.000144
O	-0.424168	-0.561161	-0.000469
H	2.284109	0.56906	-0.000054

E(RB3LYP) = -1247.503405

Atom	X	Y	Z
C	-0.803528	-0.629278	-0.028996
O	-0.867035	-1.79119	-0.315749
H	-0.053152	0.232197	-1.763369
N	0.447178	0.162931	-0.874318
C	1.596315	-0.782999	-1.131073
H	1.180078	-1.564103	-1.769371
H	2.332895	-0.220268	-1.715394
C	2.228801	-1.430087	0.097401
H	3.018838	-2.102901	-0.254457
H	2.680471	-0.710598	0.781184
H	1.493543	-2.023184	0.642392
C	0.786334	1.592977	-0.516152
H	1.004874	2.092402	-1.467169
H	-0.140087	2.018082	-0.127131
C	1.940309	1.816263	0.452289
H	2.897477	1.470823	0.050391
H	2.025913	2.896239	0.614672

H	1.760083	1.344771	1.419342
Cl	-2.298595	0.512773	-0.660932
Cl	-0.576951	-0.138991	1.778245

E(RB3LYP) = -1247.498771

Atom	X	Y	Z
C	-0.480087	-0.447594	0.636933
O	-0.508314	-1.518678	1.153279
H	-0.376022	-0.649739	-1.302811
N	0.378122	-0.296611	-0.663913
C	1.537147	-1.273146	-0.681523
H	1.077833	-2.261724	-0.727072
H	2.051881	-1.089196	-1.629243
C	2.493517	-1.199891	0.504523
H	3.265942	-1.962801	0.359736
H	2.99237	-0.233496	0.594883
H	1.976601	-1.42321	1.439874
C	0.642834	1.092554	-1.208504
H	0.782597	0.938938	-2.283328
H	-0.30029	1.624105	-1.079973
C	1.824867	1.854277	-0.625415
H	2.784438	1.400015	-0.887999
H	1.812648	2.861055	-1.056421
H	1.759951	1.957915	0.459678
Cl	-2.393256	-0.313791	-0.977748
Cl	-0.696036	1.073005	1.55291

E(RB3LYP) = -1247.544363

Atom	X	Y	Z
C	0.635115	0.561644	0.857272
O	0.782836	0.386882	2.036216
H	-1.953916	0.083248	0.046781
N	0.225586	-0.36756	-0.055459
C	0.073788	-1.743646	0.487292

H	-0.503565	-1.662442	1.411482
H	-0.533997	-2.293806	-0.23665
C	1.38753	-2.475696	0.767475
H	1.166804	-3.459006	1.197759
H	1.975096	-2.630868	-0.142088
H	1.991614	-1.919368	1.488891
C	0.274983	-0.183595	-1.522368
H	-0.429798	-0.908654	-1.940412
H	-0.116193	0.808241	-1.753337
C	1.65905	-0.36081	-2.151359
H	2.047395	-1.373153	-2.007098
H	1.592001	-0.177016	-3.229571
H	2.375325	0.351147	-1.730905
Cl	-3.236103	-0.002169	-0.141772
Cl	0.904612	2.229943	0.163169

E(RB3LYP) = -941.787809

Atom	X	Y	Z
C	-0.895624	0.584036	-0.900575
O	-0.482265	0.4053	-2.016006
N	-1.522959	-0.303218	-0.105798
C	-1.917816	-0.09799	1.297065
H	-2.037505	0.971312	1.463027
H	-2.907374	-0.554024	1.417138
C	-0.922873	-0.685681	2.302115
H	0.085842	-0.3043	2.115483
H	-1.224891	-0.413062	3.319824
H	-0.893121	-1.779208	2.247021
Cl	-0.703366	2.280311	-0.170535
C	2.874222	0.329745	-0.217174
H	3.621646	1.119518	-0.394538
H	2.256053	0.252001	-1.123991
C	3.574518	-0.989974	0.060587
H	4.218741	-1.268769	-0.780742
H	4.191861	-0.9133	0.961891

H	2.8412	-1.788711	0.21586
C	-1.719696	-1.646072	-0.700259
H	-2.487967	-2.137794	-0.096267
H	-2.125466	-1.504891	-1.706185
C	-0.454378	-2.503299	-0.767755
H	-0.028604	-2.675584	0.224998
H	-0.697053	-3.475897	-1.211208
H	0.300291	-2.022226	-1.394735
O	2.06823	0.649648	0.918393
H	1.586446	1.467998	0.718987

E(RB3LYP) = -941.753693

Atom	X	Y	Z
C	-0.040187	0.179428	0.695274
O	-0.367804	-0.21744	1.758171
N	1.090635	0.299794	0.01808
C	1.192724	0.828042	-1.353482
H	0.254139	1.326994	-1.58759
H	1.960928	1.609005	-1.336327
C	1.533401	-0.25416	-2.375665
H	0.745597	-1.012174	-2.406368
H	1.620953	0.195211	-3.371199
H	2.484957	-0.747847	-2.147117
Cl	-1.255494	2.361123	0.298106
C	-2.365395	-1.183506	-0.078127
H	-3.303251	-0.982996	-0.60718
H	-2.499973	-0.928966	0.979007
C	-1.921093	-2.622993	-0.254473
H	-2.686513	-3.299175	0.141557
H	-1.764808	-2.858199	-1.312207
H	-0.989635	-2.811843	0.288803
C	2.324675	0.132366	0.831937
H	3.136187	0.547472	0.229046
H	2.234227	0.750306	1.731574
C	2.612211	-1.321406	1.200139

H	2.731266	-1.941302	0.305711
H	3.538157	-1.375712	1.78369
H	1.805259	-1.737215	1.810262
O	-1.366391	-0.303014	-0.64575
H	-1.702984	0.645809	-0.589009

E(RB3LYP) = -941.809515

Atom	X	Y	Z
C	0.389338	0.533922	-0.564355
O	0.729617	1.408169	-1.343112
N	1.101252	-0.590808	-0.280883
C	0.733803	-1.530215	0.789103
H	-0.330636	-1.763852	0.712903
H	1.270941	-2.460777	0.580946
C	1.057516	-1.041719	2.204696
H	0.514043	-0.119158	2.428819
H	0.756833	-1.799544	2.937458
H	2.128246	-0.852207	2.330806
Cl	-3.086259	-1.56939	-0.498762
C	-1.539762	1.830397	0.015755
H	-2.56985	1.557382	0.257078
H	-1.485705	2.169833	-1.020032
C	-0.999959	2.878049	0.974842
H	-1.612959	3.784482	0.911518
H	-1.028511	2.51694	2.008298
H	0.028745	3.143348	0.715706
C	2.371298	-0.761314	-1.001925
H	2.565886	-1.837459	-1.060759
H	2.219435	-0.394897	-2.019768
C	3.559551	-0.032329	-0.368427
H	3.771204	-0.398572	0.64182
H	4.458538	-0.18419	-0.977179
H	3.359859	1.042021	-0.318079
O	-0.807139	0.578165	0.130168
H	-2.09897	-0.739479	-0.278979

E(RB3LYP) = -883.019342

Atom	X	Y	Z
C	0.779374	0.640352	0.779129
O	0.262192	1.292695	1.630229
C	1.194887	-1.442433	-0.433928
H	0.548277	-1.122544	-1.252774
H	2.237835	-1.223793	-0.676172
C	0.991337	-2.903263	-0.082374
H	-0.055924	-3.10321	0.162375
H	1.270818	-3.523226	-0.941007
H	1.612399	-3.193527	0.770646
Cl	1.698781	1.471522	-0.569572
O	0.819412	-0.67665	0.747586
O	-1.437055	0.438821	-1.121058
H	-1.140182	1.292789	-1.471356
C	-2.567232	0.675386	-0.272154
H	-3.395347	1.108653	-0.854173
H	-2.304907	1.384152	0.525609
C	-2.986838	-0.654717	0.3293
H	-3.865626	-0.523926	0.970177
H	-3.236584	-1.371849	-0.460189
H	-2.175591	-1.070272	0.935697

E(RB3LYP) = -882.988053

Atom	X	Y	Z
C	-0.034383	0.006641	0.843981
O	-0.740632	0.215338	1.761912
C	1.784418	-0.81533	-0.568118
H	1.174644	-1.423831	-1.23881
H	1.899761	0.192535	-0.969829
C	3.105817	-1.468206	-0.223222
H	2.959933	-2.452212	0.232894
H	3.686499	-1.596683	-1.142961
H	3.681662	-0.841387	0.463459
Cl	0.543295	2.200725	-0.252923

O	1.041343	-0.687414	0.693768
O	-0.934569	-0.116555	-0.722561
H	-0.603513	0.811677	-0.982876
C	-2.380794	-0.071547	-0.529907
H	-2.790624	0.326497	-1.462758
H	-2.596654	0.626823	0.283597
C	-2.89024	-1.467752	-0.235047
H	-3.980712	-1.444874	-0.135012
H	-2.631538	-2.159508	-1.04283
H	-2.473513	-1.845136	0.703738

E(RB3LYP) = -883.042781

Atom	X	Y	Z
C	-0.005405	-1.189536	0.58428
O	-0.537474	-2.047624	1.241526
C	1.929508	-0.143363	-0.45816
H	1.422526	-0.16678	-1.427144
H	1.81037	0.855724	-0.02758
C	3.394445	-0.518107	-0.578483
H	3.506498	-1.518798	-1.006733
H	3.905161	0.199536	-1.229431
H	3.880366	-0.503912	0.401717
Cl	-0.312549	3.022661	0.213293
O	1.31753	-1.11786	0.424991
O	-0.672826	-0.194146	-0.075425
H	-0.351792	1.72285	0.133689
C	-2.129114	-0.308063	-0.05637
H	-2.475407	0.709774	-0.248598
H	-2.432909	-0.615294	0.945732
C	-2.616421	-1.278972	-1.117227
H	-3.712146	-1.293362	-1.122939
H	-2.272288	-0.977867	-2.111997
H	-2.262954	-2.291832	-0.905672

Cartesian coordinates of the optimized structures for the dechlorination of CCl_4 by Co(I) species and the reaction of •CCl_3 with O_2. Geometry optimizations were performed at the TPSS-D3/D95**(Wachters-Hay for Co) level of theory.

Co(I) (closed-shell singlet)

E(RTPSS) = -2338.98139494

Co	0.038665	-0.000004	0.000106
C	-2.708669	0.663133	-0.374432
C	-3.652625	1.794921	0.067429
C	-2.817410	3.061907	-0.255394
H	-3.020467	3.905114	0.416710
C	-1.394919	2.543586	-0.118321
C	-0.218408	3.325310	0.020334
C	1.044567	2.765787	0.083726
C	2.312578	3.578417	0.290279
C	3.429737	2.565882	-0.032820
H	4.269230	2.598252	0.672281
C	2.693923	1.238584	0.010526
C	3.337514	-0.000500	0.000136
H	4.426885	-0.000662	0.000202
C	2.693548	-1.239383	-0.010360
C	3.428883	-2.566935	0.033071
C	2.311515	-3.578946	-0.290903
H	2.332378	-4.477327	0.336969
C	1.043735	-2.766068	-0.083980
C	-0.219390	-3.325237	-0.020592
C	-1.395665	-2.543194	0.118305
C	-2.818302	-3.061138	0.255220
C	-3.653136	-1.793873	-0.067474
H	-3.829874	-1.727452	-1.150377
C	-2.708907	-0.662398	0.374612
H	-2.817861	-0.459267	1.453311
N	-1.339636	1.214508	-0.148256
N	1.329435	1.399010	0.014193
N	1.329002	-1.399372	-0.014155
N	-1.339984	-1.214136	0.148608

H	2.368081	-3.904524	-1.340446
H	4.268824	-2.599468	-0.671479
H	3.836597	-2.731732	1.042333
H	2.333777	4.476291	-0.338314
H	2.369076	3.904853	1.339551
H	3.838207	2.730279	-1.041832
H	-2.997838	3.406860	-1.286228
H	-3.829458	1.728703	1.150328
H	-4.618751	1.769034	-0.449139
H	-3.021552	-3.904200	-0.417005
H	-4.619293	-1.767789	0.449026
H	-2.998896	-3.406173	1.285999
H	-2.817504	0.459997	-1.453143
H	-0.309979	-4.408140	-0.086062
H	-0.308670	4.408255	0.085560

RC (closed-shell singlet)

E(RTPSS) = -4217.86489082

Co	0.501389	0.303527	-0.981767
C	2.934951	-0.370330	0.323363
C	4.286576	0.307492	0.604396
C	3.847120	1.703515	1.120115
H	4.562511	2.503059	0.889810
C	2.515508	1.894392	0.412161
C	1.802318	3.112429	0.259458
C	0.554064	3.173555	-0.331311
C	-0.209512	4.468798	-0.553004
C	-1.633933	3.975256	-0.876222
H	-2.115190	4.523361	-1.695232
C	-1.404411	2.513452	-1.214621
C	-2.379399	1.692145	-1.785599
H	-3.333658	2.149538	-2.043797
C	-2.228361	0.327731	-2.041040
C	-3.305657	-0.530590	-2.678395
C	-2.783413	-1.958687	-2.423832

H	-2.901921	-2.634605	-3.278589
C	-1.324595	-1.728921	-2.066864
C	-0.388867	-2.743861	-1.991677
C	0.947803	-2.522071	-1.568059
C	2.051303	-3.563011	-1.478177
C	3.069581	-2.866390	-0.538196
H	2.828510	-3.097293	0.509232
C	2.802962	-1.375506	-0.812335
H	3.390885	-1.019317	-1.675029
N	2.034538	0.751541	-0.067032
N	-0.159280	2.080876	-0.833191
N	-1.088749	-0.385997	-1.766958
N	1.357507	-1.318606	-1.179677
C	-1.332323	-0.711812	2.383393
Cl	0.129346	0.222989	2.881129
Cl	-2.399300	0.333112	1.383613
Cl	-0.834400	-2.150515	1.427224
H	-3.295334	-2.414282	-1.562600
H	-4.295727	-0.341288	-2.246178
H	-3.366518	-0.313949	-3.756167
H	-0.156766	5.134379	0.316368
H	0.223277	5.004636	-1.411432
H	-2.290132	4.050064	0.004925
H	3.696604	1.695231	2.211777
H	4.850655	0.414363	-0.333121
H	4.900153	-0.246997	1.323521
H	1.686317	-4.523919	-1.094047
H	4.108642	-3.155812	-0.732038
H	2.485976	-3.745699	-2.474063
H	2.523515	-0.821943	1.241538
H	-0.699347	-3.754275	-2.251590
H	2.251208	4.036725	0.619048
Cl	-2.220914	-1.247788	3.861685

TS$_{SN2}$ (closed-shell singlet)

E(RTPSS) = -4217.83642503

Co	-0.193904	-0.033556	-0.991353
C	-2.855515	-0.666272	-0.183573
C	-3.867512	-1.798784	-0.425982
C	-2.991595	-3.065977	-0.239102
H	-3.309499	-3.918098	-0.852068
C	-1.612357	-2.558560	-0.622947
C	-0.466388	-3.354110	-0.899155
C	0.773773	-2.810668	-1.170153
C	1.995676	-3.644169	-1.516920
C	3.142032	-2.613273	-1.498681
H	3.814918	-2.688351	-2.360971
C	2.403058	-1.290085	-1.467992
C	3.034414	-0.056259	-1.635944
H	4.104009	-0.064053	-1.837833
C	2.404825	1.188683	-1.547755
C	3.128695	2.506323	-1.742289
C	2.102603	3.530398	-1.217182
H	2.010216	4.426910	-1.840098
C	0.813588	2.729558	-1.171813
C	-0.434188	3.292651	-1.000051
C	-1.623921	2.515487	-0.932088
C	-3.041455	3.050144	-0.821153
C	-3.823702	1.792692	-0.358624
H	-3.816063	1.731383	0.738590
C	-2.979767	0.647264	-0.944436
H	-3.271949	0.429104	-1.985286
N	-1.556942	-1.236574	-0.637447
N	1.067398	-1.446737	-1.210761
N	1.074327	1.362266	-1.286309
N	-1.591362	1.193499	-0.961882
C	0.497950	0.057062	2.053460
Cl	-0.104430	-1.554439	2.175986
Cl	2.138060	0.304118	1.592187
Cl	-0.584683	1.400033	2.139994

H	2.358522	3.853873	-0.197616
H	4.086898	2.529546	-1.210366
H	3.337140	2.657519	-2.811980
H	2.136408	-4.469619	-0.809759
H	1.867548	-4.081078	-2.517487
H	3.755071	-2.706792	-0.590141
H	-2.981117	-3.394976	0.812058
H	-4.239998	-1.748919	-1.458660
H	-4.720987	-1.759223	0.259404
H	-3.110501	3.893529	-0.123239
H	-4.862328	1.776350	-0.706133
H	-3.387385	3.401813	-1.805784
H	-2.758974	-0.443675	0.892518
H	-0.508033	4.375040	-0.918963
H	-0.567635	-4.437448	-0.901615
Cl	1.065400	0.186957	4.663659

IM$_{SN2}$ (closed-shell singlet)

E(RTPSS) = -4217.90228768

Co	0.335440	0.173250	-0.728455
C	2.931682	0.202537	0.484619
C	4.113448	1.178714	0.616799
C	3.405372	2.530263	0.899169
H	3.933701	3.403074	0.498974
C	2.048350	2.331118	0.253213
C	1.081139	3.345902	0.006627
C	-0.149309	3.091602	-0.560034
C	-1.206133	4.150121	-0.801224
C	-2.243824	3.425349	-1.681480
H	-2.230583	3.785033	-2.719331
C	-1.783400	1.984089	-1.641625
C	-2.494408	0.941110	-2.238874
H	-3.439173	1.191730	-2.715972
C	-2.094083	-0.395633	-2.274135
C	-2.902783	-1.490067	-2.935498

C	-2.205904	-2.772798	-2.439283
H	-2.064050	-3.532155	-3.214844
C	-0.888306	-2.256218	-1.900784
C	0.201814	-3.049496	-1.613958
C	1.439738	-2.510643	-1.165674
C	2.736109	-3.278473	-0.984075
C	3.573958	-2.303872	-0.114285
H	3.370857	-2.483896	0.949722
C	3.007248	-0.935084	-0.524538
H	3.492014	-0.562375	-1.441536
N	1.819896	1.060345	-0.012954
N	-0.587850	1.842258	-0.998924
N	-0.932277	-0.871712	-1.740498
N	1.582430	-1.235011	-0.863981
C	-0.507424	-0.200412	1.023310
Cl	-0.131896	1.062161	2.291158
Cl	-2.329289	-0.245580	0.922523
Cl	0.016833	-1.801998	1.720407
H	-2.768090	-3.230991	-1.613152
H	-3.961788	-1.425583	-2.663072
H	-2.829731	-1.385175	-4.027397
H	-1.635494	4.451548	0.163820
H	-0.780619	5.043119	-1.271476
H	-3.271930	3.517743	-1.312546
H	3.255498	2.691759	1.977795
H	4.658536	1.232282	-0.335020
H	4.810696	0.887577	1.408385
H	2.564452	-4.255187	-0.516812
H	4.650974	-2.384991	-0.291862
H	3.200350	-3.452316	-1.966638
H	2.618762	-0.195902	1.463528
H	0.125605	-4.120703	-1.782039
H	1.320611	4.369776	0.282855
Cl	-2.592859	-1.233520	5.045897

Co-C (closed-shell singlet)

E(RTPSS) = -3757.55085808

Co	-0.001513	0.005094	-0.424298
C	2.783261	0.671897	-0.287445
C	3.710315	1.786880	-0.801480
C	2.869556	3.067065	-0.552748
H	3.039695	3.862037	-1.287642
C	1.448615	2.540205	-0.592979
C	0.265154	3.328308	-0.656713
C	-1.001801	2.785460	-0.657655
C	-2.275098	3.605839	-0.688237
C	-3.371200	2.555118	-0.954950
H	-3.781682	2.632988	-1.970878
C	-2.632949	1.241844	-0.810193
C	-3.266486	-0.000015	-0.889399
H	-4.346359	-0.000440	-1.019252
C	-2.629612	-1.239951	-0.818931
C	-3.364224	-2.559176	-0.912585
C	-2.291390	-3.575921	-0.474637
H	-2.257009	-4.480782	-1.089639
C	-1.008238	-2.777144	-0.566834
C	0.255198	-3.327593	-0.546298
C	1.431074	-2.541416	-0.696678
C	2.845175	-3.068463	-0.855258
C	3.699425	-1.805623	-0.565646
H	3.897594	-1.727325	0.511553
C	2.758107	-0.671750	-1.002768
H	2.825925	-0.495327	-2.088499
N	1.415974	1.225637	-0.497712
N	-1.290113	1.420631	-0.644621
N	-1.283882	-1.414425	-0.676042
N	1.396131	-1.223743	-0.726743
C	-0.114644	-0.006882	1.548773
Cl	0.344314	1.593970	2.306976
Cl	-1.804123	-0.348279	2.157289
Cl	0.968755	-1.262678	2.314517

H	-2.441873	-3.880771	0.570742
H	-4.262395	-2.562669	-0.285698
H	-3.679777	-2.724806	-1.952576
H	-2.409494	4.091805	0.287629
H	-2.228162	4.391858	-1.449520
H	-4.211938	2.610991	-0.253772
H	3.055762	3.487107	0.447669
H	3.886227	1.656757	-1.877553
H	4.674536	1.801172	-0.284254
H	3.042495	-3.904378	-0.174159
H	4.651045	-1.795932	-1.106269
H	2.987592	-3.431654	-1.884320
H	2.902180	0.508451	0.796016
H	0.352777	-4.406897	-0.461146
H	0.363411	4.410247	-0.699869

TS_{SET} (open-shell singlet)

E(UTPSS) = -4217.85572411

Co	0.436668	0.242450	-1.006154
C	2.923623	-0.460634	0.191008
C	4.307843	0.181773	0.384732
C	3.938993	1.618344	0.842286
H	4.668224	2.380249	0.541003
C	2.582521	1.821251	0.189155
C	1.896284	3.057721	0.029401
C	0.615226	3.138050	-0.477921
C	-0.126985	4.446443	-0.690497
C	-1.573446	3.985634	-0.960161
H	-2.055801	4.509697	-1.793553
C	-1.404144	2.504503	-1.238462
C	-2.427461	1.695849	-1.738438
H	-3.377413	2.174418	-1.969887
C	-2.331962	0.318754	-1.951908
C	-3.466888	-0.520391	-2.506472
C	-2.967232	-1.959924	-2.270363

H	-3.125555	-2.629407	-3.123222
C	-1.493290	-1.766435	-1.961506
C	-0.584525	-2.803789	-1.889853
C	0.777255	-2.611653	-1.526067
C	1.847174	-3.687503	-1.444980
C	2.935533	-2.993581	-0.583642
H	2.741639	-3.178795	0.482071
C	2.706758	-1.504420	-0.898455
H	3.259978	-1.198813	-1.802317
N	2.050575	0.678112	-0.207428
N	-0.159324	2.052979	-0.894631
N	-1.209980	-0.424613	-1.708578
N	1.247588	-1.418549	-1.199922
C	-1.289346	-0.618684	2.472930
Cl	0.559911	0.410010	2.936432
Cl	-2.330032	0.496918	1.527605
Cl	-0.892246	-2.074938	1.503184
H	-3.458655	-2.409249	-1.395098
H	-4.418844	-0.298356	-2.010073
H	-3.593162	-0.307472	-3.578380
H	-0.030255	5.115989	0.171568
H	0.292530	4.964208	-1.565306
H	-2.209041	4.116654	-0.071686
H	3.827042	1.673460	1.936612
H	4.839910	0.218994	-0.576332
H	4.927533	-0.355985	1.110601
H	1.465345	-4.617475	-1.006332
H	3.952024	-3.326369	-0.820345
H	2.220038	-3.922125	-2.454361
H	2.528731	-0.850053	1.143475
H	-0.932488	-3.811748	-2.105923
H	2.394536	3.978176	0.326915
Cl	-2.067277	-1.069373	4.031362

IM$_{SET}$ (open-shell singlet)

E(UTPSS) = -4217.86200676

Co	0.150716	0.215256	-1.127544
C	2.825894	-0.520107	-0.432552
C	4.228515	0.102951	-0.536458
C	3.976191	1.556152	-0.051091
H	4.641175	2.301159	-0.503938
C	2.521022	1.770910	-0.423880
C	1.832968	3.019658	-0.459455
C	0.476739	3.120195	-0.682317
C	-0.280208	4.435289	-0.735409
C	-1.757383	3.992296	-0.722247
H	-2.379590	4.504756	-1.464825
C	-1.666804	2.504591	-0.995070
C	-2.783343	1.703831	-1.253042
H	-3.754116	2.194346	-1.281700
C	-2.756238	0.322760	-1.459436
C	-3.998448	-0.501170	-1.732037
C	-3.477027	-1.950302	-1.648050
H	-3.800870	-2.582695	-2.482076
C	-1.969090	-1.779721	-1.625954
C	-1.077783	-2.829611	-1.712582
C	0.333659	-2.658989	-1.626105
C	1.376571	-3.759050	-1.723025
C	2.631441	-3.071361	-1.121537
H	2.654259	-3.223210	-0.033799
C	2.377634	-1.584438	-1.428729
H	2.741734	-1.315730	-2.434381
N	1.914189	0.630868	-0.679927
N	-0.385295	2.046450	-0.912556
N	-1.624698	-0.440448	-1.456970
N	0.888628	-1.477142	-1.438127
C	-0.722793	-0.580422	2.763699
Cl	1.847651	0.431363	2.898192
Cl	-1.776185	0.670904	2.044276
Cl	-0.650928	-2.037246	1.733334

H	-3.796048	-2.436806	-0.715902
H	-4.793027	-0.278819	-1.010384
H	-4.383311	-0.261582	-2.733428
H	-0.010679	5.090739	0.099889
H	-0.030146	4.960392	-1.667948
H	-2.219038	4.148047	0.263419
H	4.060692	1.629553	1.043760
H	4.562308	0.107284	-1.583707
H	4.970585	-0.427190	0.069826
H	1.068733	-4.664448	-1.186741
H	3.570193	-3.437702	-1.550070
H	1.528171	-4.029576	-2.779376
H	2.622110	-0.860482	0.595703
H	-1.474341	-3.835004	-1.833947
H	2.397941	3.932252	-0.283405
Cl	-1.205536	-0.971257	4.439577

Co(II) (doublet)

E(UTPSS) = -2338.84966506

Co	0.044124	-0.000014	-0.000126
C	-2.726712	0.665145	-0.374767
C	-3.665384	1.800073	0.072953
C	-2.818087	3.069190	-0.213475
H	-3.010370	3.895642	0.480636
C	-1.398066	2.543094	-0.094506
C	-0.215894	3.327951	0.034037
C	1.049812	2.779414	0.070265
C	2.320328	3.595779	0.224346
C	3.440364	2.561076	-0.008222
H	4.227316	2.586707	0.754309
C	2.695548	1.241758	0.007390
C	3.336439	-0.000045	0.000048
H	4.424492	-0.000063	0.000126
C	2.695508	-1.241837	-0.007335
C	3.440266	-2.561175	0.008484

C	2.320241	-3.595824	-0.224322
H	2.345701	-4.434282	0.480455
C	1.049725	-2.779444	-0.070337
C	-0.215988	-3.327936	-0.034134
C	-1.398143	-2.543052	0.094456
C	-2.818165	-3.069133	0.213458
C	-3.665472	-1.799946	-0.072702
H	-3.861061	-1.716176	-1.150344
C	-2.726686	-0.665063	0.374907
H	-2.828263	-0.464436	1.454000
N	-1.362762	1.224822	-0.145344
N	1.341311	1.416666	0.003654
N	1.341274	-1.416678	-0.003806
N	-1.362801	-1.224786	0.145326
H	2.362821	-4.012726	-1.239821
H	4.227381	-2.586862	-0.753881
H	3.923025	-2.693320	0.987054
H	2.345891	4.434167	-0.480511
H	2.362762	4.012803	1.239797
H	3.923359	2.693216	-0.986676
H	-2.981726	3.444666	-1.235234
H	-3.860740	1.716369	1.150646
H	-4.621142	1.792189	-0.460897
H	-3.010482	-3.895396	-0.480877
H	-4.621114	-1.792058	0.461354
H	-2.981762	-3.444902	1.235107
H	-2.828419	0.464527	-1.453850
H	-0.313048	-4.409163	-0.100621
H	-0.312925	4.409178	0.100545

CCl_4 (closed-shell singlet)

E(RTPSS) = -1878.86867183

C	0.000000	0.000000	0.000000
Cl	1.038163	1.038163	1.038163
Cl	-1.038163	-1.038163	1.038163

Cl	1.038163	-1.038163	-1.038163
Cl	-1.038163	1.038163	-1.038163

•CCl_3, **I** (doublet)

E(UTPSS) = -1418.63613859

C	0.000000	0.000000	0.320072
Cl	0.000000	1.709313	-0.037656
Cl	1.480309	-0.854657	-0.037656
Cl	-1.480309	-0.854657	-0.037656

O_2 (triplet)

E(UTPSS) = -150.382863259

O	0.000000	0.000000	0.618228
O	0.000000	0.000000	-0.618228

•O_2CCl_3, **II** (doublet)

E(UTPSS) = -1569.05265753

O	-2.051862	-0.973493	0.000000
O	-1.466176	0.226729	0.000000
C	0.024716	0.113373	0.000000
Cl	0.548941	1.809881	0.000000
Cl	0.548941	-0.749239	1.469936
Cl	0.548941	-0.749239	-1.469936

$(CCl_3O_2)_2$, **III** (closed-shell singlet)

E(RTPSS) = -3138.14803964

O	-0.558496	1.221069	-1.070476
O	-1.401411	-0.298139	-0.917540
C	-2.443970	-0.109529	-0.083811
Cl	-3.219016	-1.750645	-0.094602
Cl	-1.955519	0.325917	1.621688
Cl	-3.626138	1.146268	-0.666242
O	0.416444	1.266834	-0.298671
O	1.772450	0.559316	-1.079332

C	2.514390	-0.039298	-0.115078
Cl	3.905956	-0.679077	-1.081687
Cl	3.107982	1.121158	1.153121
Cl	1.654121	-1.404778	0.721926

•$OCCl_3$, **IV** (doublet)

E(UTPSS) = -1493.87090415

O	-0.330108	-0.004708	1.578020
C	0.033697	0.000023	0.331213
Cl	0.918740	1.479507	-0.232070
Cl	0.935302	-1.469095	-0.233895
Cl	-1.710591	-0.008204	-0.393532

$O{=}CCl_2$, **V** (closed-shell singlet)

E(RTPSS) = -1033.75710828

C	-0.000336	0.500872	0.000758
O	-0.000699	1.700123	-0.000257
Cl	-1.462422	-0.488704	-0.000073
Cl	1.462870	-0.488132	-0.000073

8. References

(1) Shichijo, K.; Watanabe, M.; Hisaeda, Y.; Shimakoshi, H. Development of Visible Light-Driven Hybrid Catalysts Composed of Earth Abundant Metal Ion Modified TiO_2 and B_{12} Complex. Bull. Chem. Soc. Jpn. 2022, 95 (7), 1016–1024.

(2) Gaussian 16, Revision C.01, Frisch, M. J.; Trucks, G. W.; Schlegel, H. B.; Scuseria, G. E.; Robb, M. A.; Cheeseman, J. R.; Scalmani, G.; Barone, V.; Petersson, G. A.; Nakatsuji, H.; Li, X.; Caricato, M.; Marenich, A. V.; Bloino, J.; Janesko, B. G.; Gomperts, R.; Mennucci, B.; Hratchian, H. P.; Ortiz, J. V.; Izmaylov, A. F.; Sonnenberg, J. L.; Williams-Young, D.; Ding, F.; Lipparini, F.; Egidi, F.; Goings, J.; Peng, B.; Petrone, A.; Henderson, T.; Ranasinghe, D.; Zakrzewski, V. G.; Gao, J.; Rega, N.; Zheng, G.; Liang, W.; Hada, M.; Ehara, M.; Toyota, K.; Fukuda, R.; Hasegawa, J.; Ishida, M.; Nakajima, T.; Honda, Y.; Kitao, O.; Nakai, H.; Vreven, T.; Throssell, K.; Montgomery, J. A., Jr.; Peralta, J. E.; Ogliaro, F.; Bearpark, M. J.; Heyd, J. J.; Brothers, E. N.; Kudin, K. N.; Staroverov, V. N.; Keith, T. A.; Kobayashi, R.; Normand, J.; Raghavachari, K.; Rendell, A. P.; Burant, J. C.; Iyengar, S. S.; Tomasi, J.; Cossi, M.; Millam, J. M.; Klene, M.; Adamo, C.; Cammi, R.; Ochterski, J. W.; Martin, R. L.; Morokuma, K.; Farkas, O.; Foresman, J. B.; Fox, D. J. Gaussian, Inc., Wallingford CT, 2016.

(3) Tao, J. M.; Perdew, J. P.; Staroverov, V. N.; Scuseria, G. E. Climbing the Density Functional Ladder: Nonempirical Meta-Generalized Gradient Approximation Designed for Molecules and Solids. *Phys. Rev. Lett.* **2003**, *91*, 146401.

(4) (a) Wachters, A. J. H. Gaussian Basis Set for Molecular Wavefunctions Containing Third-Row Atoms. *J. Chem. Phys.* **1970**, *52*, 1033-1036. (b) Hay, P. J. Gaussian Basis Sets for Molecular Calculations: Representation of 3D Orbitals in Transition-Metal Atoms. *J. Chem. Phys.* **1977**, *66* 4377-4384. (c) Raghavachari, K.; Trucks, G. W. Highly Correlated Systems: Excitation Energies of First Row Transition Metals Sc–Cu. *J. Chem. Phys.* **1989**, *91*, 1062-1065.

(5) Dunning, T. H., Jr.; Hay, P. J. In Modern Theoretical Chemistry; Schaefer, H. F., III, Ed.; Plenum: New York, 1977; Vol. 3, pp 1−28.

(6) Grimme, S.; Antony, J.; Ehrlich, S.; Krieg, H. A Consistent and Accurate *Ab Initio* Parametrization of Density Functional Dispersion Correction (DFT-D) for the 94 Elements H–Pu. *J. Chem. Phys.,* **2010**, *132*, 154104.

(7) Tomasi, J.; Mennucci, B.; Cammi, R. Quantum Mechanical Continuum Solvation Models. *Chem. Rev.* **2005**, *105*, 2999-3094.

(8) (a) Becke, A. D. Density-Functional Exchange-Energy Approximation with Correct Asymptotic Behavior. *Phys. Rev. A Gen. Phys.*, **1988**, *38*, 3098-3100. (b) Lee, C.; Yang,

W.; Parr, R. G. Development of the Colle-Salvetti Correlation-Energy Formula into a Functional of the Electron Density. *Phys. Rev. B Condens. Matter*, **1988**, *37*, 785-789. (c) Becke, A. D. Density - Functional Thermochemistry. III. The Role of Exact Exchange. *J. Chem. Phys.*, **1993**, *98*, 5648-5652.

(9) (a) Ditchfield, R.; Hehre, W. J.; Pople, J. A. Self-Consistent Molecular-Orbital Methods. IX. An Extended Gaussian-Type Basis for Molecular-Orbital Studies of Organic Molecules. *J. Chem. Phys.* **1971**, *54*, 724–728. (b) Francl, M. M.; Pietro, W. J.; Hehre, W. J.; Binkley, J. S.; Gordon, M. S.; DeFrees, D. J.; Pople, J. A. Self-Consistent Molecular Orbital Methods. XXIII. A Polarization-Type Basis Set for Second-Row Elements. *J. Chem. Phys.* **1982**, *77*, 3654–3665. (c) Gordon, M. S.; Binkley, J. S.; Pople, J. A.; Pietro, W. J.; Hehre, W. J. Self-Consistent Molecular-Orbital Methods. 22. Small Split-Valence Basis Sets for Second-Row Elements. *J. Am. Chem. Soc.* **1982**, *104*, 2797–2803. (d) Hariharan, P. C.; Pople, J. A. The Influence of Polarization Functions on Molecular Orbital Hydrogenation Energies. *Theor. Chim. Acta* **1973**, *28*, 213–222. (e) Hehre, W. J.; Ditchfield, R.; Pople, J. A. Self-Consistent Molecular Orbital Methods. XII. Further Extensions of Gaussian-Type Basis Sets for Use in Molecular Orbital Studies of Organic Molecules. *J. Chem. Phys.* **1972**, *56*, 2257–2261.
